# A Novel Risk Defining System for Pediatric T-Cell Acute Lymphoblastic Leukemia From CCCG-ALL-2015 Group

**DOI:** 10.3389/fonc.2022.841179

**Published:** 2022-02-28

**Authors:** Xiaoming Liu, Yao Zou, Li Zhang, Ye Guo, Yumei Chen, Wenyu Yang, Xiaojuan Chen, Shuchun Wang, Yingchi Zhang, Min Ruan, Lixian Chang, Xiaoyan Zhang, Beibei Zhao, Ranran Zhang, Aoli Zhang, Lipeng Liu, Luyang Zhang, Meihui Yi, Xiaofan Zhu

**Affiliations:** State Key Laboratory of Experimental Hematology, National Clinical Research Center for Blood Diseases, Haihe Laboratory of Cell Ecosystem, Institute of Hematology & Blood Diseases Hospital, Chinese Academy of Medical Sciences & Peking Union Medical College, Tianjin, China

**Keywords:** children, T-cell acute lymphoblastic leukemia (T-ALL), risk, efficacy, CCCG-ALL-2015

## Abstract

**Objective:**

T-cell acute lymphoblastic leukemia (T-ALL) is a rare hematological malignancy with a poor prognosis. The present study aims to identify the precise risk grouping of children with T-ALL.

**Methods:**

We analyzed the outcomes for 105 consecutive patients treated using the Chinese Children’s Cancer Group ALL-2015 (CCCG-ALL-2015) protocol registered with the Chinese Clinical Trial Registry (ChiCTR-IPR-14005706) between 2015 and 2020 in our center. Nine out of 21 clinical and biological indicators were selected for the new scoring system based on the analysis in this study.

**Results:**

The 5-year overall survival (OS), event-free survival (EFS), and disease-free survival (DFS) rates for the 105 patients were 83.1 ± 4.8%, 72.4 ± 5.6%, and 78.4 ± 3.6%, respectively. Based on the new scoring system, 90 evaluable children were regrouped into low-risk (n=22), intermediate-risk (n=50), and high-risk (n=18) groups. The 5-year survival (OS, EFS, and RFS) rates for all patients in the low-risk group were 100%, significantly higher than the rates for those in the intermediate-risk group (91.2 ± 5.2%, 74.4 ± 8.6%, and 82.5 ± 6.2%, respectively) and high-risk group (59.0 ± 13.2%, 51.9 ± 12.4%, and 51.9 ± 12.4%, respectively) (all P values < 0.01).

**Conclusion:**

The CCCG-ALL-2015 program significantly improved the treatment outcomes for childhood T-ALL as compared with the CCCG-ALL-2008 protocol. Our new refined risk grouping system showed better stratification among pediatric T-ALL patients and better potential in evaluating therapeutic efficacy.

## Introduction

T-cell acute lymphoblastic leukemia (T-ALL) is an aggressive cancer caused by the malignant proliferation of precursor T cells. T-ALL accounts for 15% of all childhood acute lymphoblastic leukemia (ALL) and is characterized by a high white blood cell (WBC) count, central nervous system (CNS) infiltration, and mediastinal enlargement (1). At present, most T-ALL regimens still follow the combination chemotherapy regimen for B-ALL, which includes glucocorticoids (dexamethasone or prednisolone), asparaginase, vincristine, thiopurine, methotrexate, and other drugs. However, there are significant differences in the pathogenic mechanisms and treatment responses for T-ALL and B-ALL. T-ALL usually has a high degree of malignancy, slow clearance of tumor cells after treatment, and a high early recurrence rate (2). Despite decades of research, the 5-year overall survival (OS) is only about 80% even in advanced international treatment institutions (3, 4), and the 5-year OS for Chinese children with T-ALL has been hovering around 60-70% (5, 6).

In recent years, with the rapid development of detection methods, new treatment methods for T-ALL have been continuously explored (7–10). However, treatment for childhood T-ALL has not advanced much. Various combined treatments based on a multi-factor risk model are currently used in clinical practice to achieve better outcomes for childhood T-ALL. In January 2015, the Chinese Children’s Cancer Group (CCCG) proposed a multicenter study for the treatment of pediatric ALL using a standard protocol called CCCG-ALL-2015, which includes guidelines for patient risk grading, chemotherapy regimes in different phases, evaluations of therapeutic efficacy, etc. The National Clinical Research Center for Blood Diseases has participated in this study since 2015. Here, we report the treatment results for this clinical trial from our center.

## Methods and Materials

### Study Population

**Figure 1:**
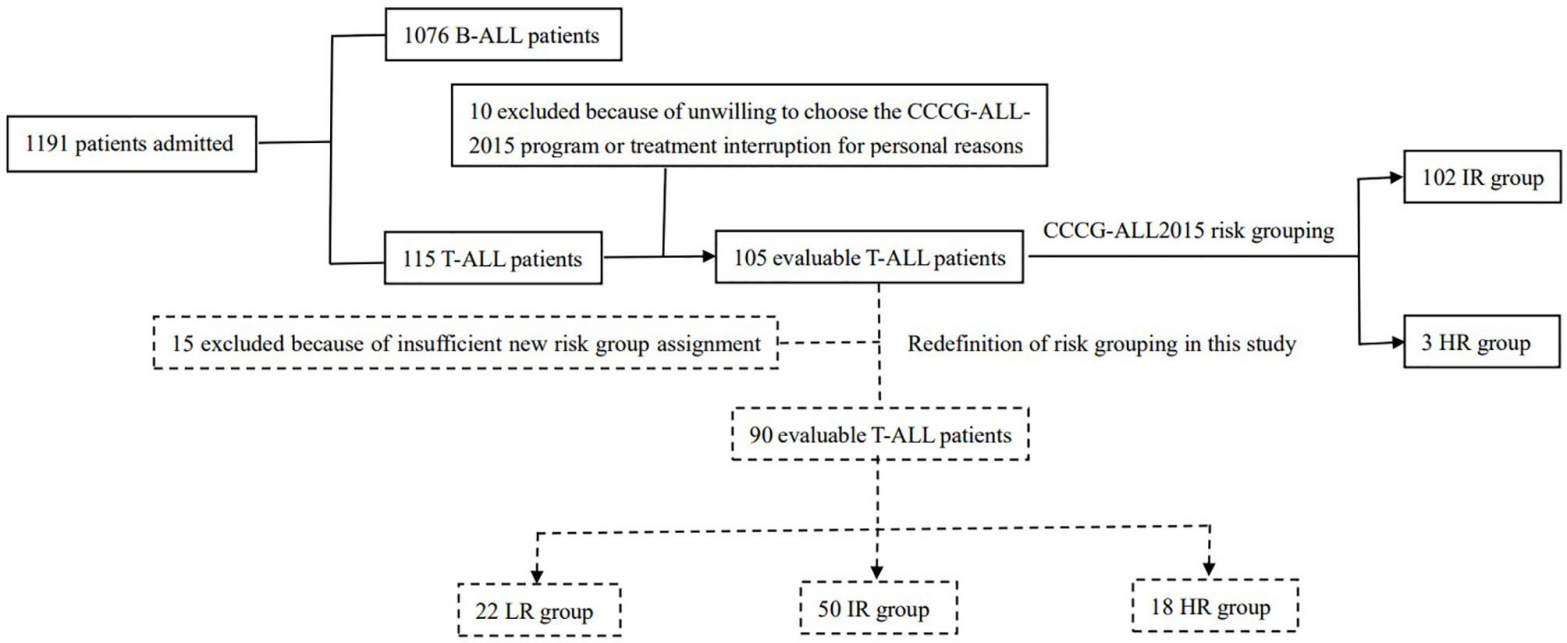
The composition and distribution of patients.

From May 1, 2015 to October 23, 2020, 115 children newly diagnosed with T-ALL were admitted to our center, accounting for 9.7% (115/1191) of children newly diagnosed with ALL admitted to our center during this period. Among these 115 children, 10 were excluded from this study because they were unwilling to participate in the CCCG-ALL-2015 program or the treatment was interrupted due to personal reasons (seven of these children survived, one died, and two were lost to follow-up). A total of 105 patients aged 1-15 years were enrolled in the CCCG-ALL-2015 study (ClinicalTrials.gov identifier: ChiCTRIPR-14005706) with a median follow-up time of 36 (2-72) months. The clinical study was approved by the Ethics Committee of Blood Diseases Hospital, Chinese Academy of Medical Sciences (No. IIT2015010-EC-1) and patients were only recruited when written informed consent was obtained from the parents or legal guardian. The composition and distribution of patients are shown in **Figure 1**.

### Entry Criteria and Related Testing Indicators

ALL was diagnosed if at least 25% of lymphoblasts were present in the bone marrow (BM). T-cell lineage features of leukemic cells were determined according to standard techniques (11, 12) (**Supplementary Table 1**). Enrollment in the CCCG-ALL-2015 classification/biology study was required for study entry (13, 14). Minimal residual disease (MRD) testing was performed at our hospital by flow cytometry using established methodologies (12) on days 19 and 46 of treatment (**Supplementary Table 2**). The sensitivity of MRD testing was 0.01%. Cytogenetic studies were tested by karyotyping (15), fluorescence *in situ* hybridization (FISH) (16) (including CDKN2A/CEP9, Myc, MLL rearrangement, P53), polymerase chain reaction (PCR) (17) (SIL-TAL1), fragment analysis (18) (WT1), and targeted next-generation sequencing (NGS) (19, 20), which included 112 genetic mutations selected based on known or suspected involvement in the pathogenesis of malignant hematologic disorder (**Supplementary Table 3**). Before systemic therapy, cerebrospinal fluid (CSF) was obtained for stratification according to the state of the CNS: CNS1, no leukemia blasts in the CSF; CNS2, WBC < 5/mL with leukemia blasts; and CNS3, WBC ≥ 5/mL with leukemia blasts or clinical symptoms of cranial nerve palsies, brain/eye involvement, or hypothalamic syndrome.

### Risk Assessment

#### Initial Risk Grouping in CCCG-ALL-2015 Protocol

In the CCCG-ALL-2015 study, all children with T-ALL were directly placed in the intermediate-risk (IR) group. Patients whose MRD was ≥ 1% at 46 days or who had leukemia blasts ≥ 5% without MRD markers or with mixed lineage leukemia (MLL) fusion genes, age <6 months, and WBC ≥ 300 × 10^9^/L were placed in the high-risk (HR) group.

#### Parameter Acquisition and Redefinition of the New Risk Score Matrix

Univariate and multivariate analyses were used to analyze the correlation between 21 clinical biological indicators and prognosis (DFS, EFS, or OS) in 105 children with T-ALL, including gender, age, initial WBC, initial hemoglobin (Hb), initial platelet count, initial percentage of blasts in BM, initial percentage of blasts in the peripheral blood (PB), CNS involvement, mediastinal mass, hepatomegaly, splenomegaly, immunophenotype, SIL-TAL1 translocation, Myc, MLL rearrangement, CDKN2A/CEP9, WT1, karyotype, dexamethasone response (DR), MRD on day 19, and MRD on day 46, were selected due to their statistical significance. The nine most relevant clinical indicators that showed significance in one or more analyses were selected and a new risk score matrix was developed based on their performances. For any variables (Kaplan-Meier method and Cox proportional hazards model) with a P value less than 0.05, the risk side score was 0.5 (initial percentage of blasts in BM, initial percentage of blasts in PB, and karyotype had a median level, defined as 0.3; each MRD had four levels, and the risk score increased by 0.5 for each level increase). The nine clinical indicators and scores are shown in **Supplementary Table 4**.

### Treatment

All treatments were divided into three stages: induction treatment, consolidation treatment, and continued treatment. Induction treatment consisted of pretreatment (dexamethasone 6 mg/m^2^, d1-4, 1 cycle), vincristine + daunorubicin + Peg-Asp + prednisone (VDLP; d5-28, 1 cycle), cyclophosphamide + cytarabine + 6-mercaptopurine (CAM; d29-35, 1 cycle) and vincristine + Peg-Asp + cyclophosphamide + cytarabine + 6-mercaptopurine (VLCAM; d50-57, 1 cycle). Consolidation treatment consisted of high-dose methotrexate + 6-mercaptopurine (HD-MTX; d1-14, 4 cycles). Continuing treatment consisted of interval treatment (6-mercaptopurine + VDLD; d1-21, 4 cycle and 6-mercaptopurine + VDLD; d1-28, 1 cycle) + re-induction treatment (vincristine + high-dose cytarabine + Peg-Asp + dexamethasone, VALD; d1-21, 1 cycle) + maintenance treatment (6-mercaptopurine + methotrexate + cyclophosphamide + cytarabine + vincristine + dexamethasone, MM+CAVD; d1-50, 5 cycles) + MM+CAVD/CA random (d1-56, 7 cycles). The total course of treatment was 2.5 years. MRD was routinely detected on “Day 19” (in the middle of VDLP induction therapy) and “day 46” (when VDLP+CAM chemotherapy had ended and the patient’s WBC, Hb, and platelet levels had recovered) during the induction treatment phase. If the MRD was not negative on day 46, the MRD was re-examined before the next stage of treatment until it became negative. All three patients in the classical HR group had indications for hematopoietic stem cell transplantation (HSCT). The transplantation was started from the end of HDMTX to the period before maintenance treatment. The MRD before transplantation should be negative if possible. Patients with positive MRD were given one to two courses of DAEL (Dexamethasone + Cytarabine + Etoposide + Peg asparaginase) (the number of DAELs was determined based on the MRD after the course of treatment). However, if there was no suitable donor or the parents of the patient refused the transplant, patients in the HR group continued chemotherapy (**Supplementary Table 5**).

### Definitions of Treatment Response

DR was defined as the absolute number of leukemia blasts in the PB after 4 days of dexamethasone treatment. Patients with less than 1000/µL remaining peripheral blasts were defined as dexamethasone good responders (DGRs); otherwise, they were defined as dexamethasone poor responders (DPRs). Complete remission (CR) was defined as the following conditions: 1) No clinical symptoms and signs caused by leukemia cell infiltration, or no extramedullary leukemia infiltration; 2) Blood routine: Hb ≥90 g/L, absolute value of neutrophils≥1.5×10^9^/L, absolute platelet value ≥100×10^9^/L, no leukemia cells were found in the PB; and 3) Normal BM cellularity with less than 5% leukemic cells on day 46 of treatment. Partial remission (PR) was defined as normal BM cellularity with less than or equal to 20% but more than 5% leukemic cells on day 46 of treatment, or one of the clinical and blood routines did not meet the criteria for CR. No remission (NR) was defined when the clinical, blood routine, and BM findings did not reach the standard for CR, and BM leukemia cells were more than 20%, including for patients who failed the treatment. Relapse was defined as recurrence with more than 25% leukemia blasts in the BM or local infiltration in other sites.

### MRD Classification Standard

MRD-1 level, MRD<10^-4^; MRD-2 level, 10^-4^≤MRD<10^-3^; MRD-3 level, 10^-3^≤MRD<10^-2^; MRD-4 level, MRD≥10^-2^.

### Statistical Analysis

Overall survival (OS) was defined as the time from the diagnostic date through the date of death due to any reason or the last follow-up examination. Event-free survival (EFS) was estimated from the date of diagnosis until the date of one of the following events was met: relapse, refractory disease, second malignancy, HSCT (MRD-positive but not relapsed or refractory), withdrawal due to economic reasons, or death from any reason. Relapse-free survival (RFS) was defined as the time from CR to the date of relapse. Induction failure or induction death was an event at time zero. Survival rates were estimated using the Kaplan-Meier method, and the differences between subgroups were evaluated using the log-rank test (21, 22). The Cox regression analysis was used for univariate and multivariate analyses (23). The Fisher’s exact test was used to compare the differences in categorical variables among the groups. The Mann-Whitney U-test was used to compare the differences in continuous variables among two or more groups, respectively. P < 0.05 was considered significant for all comparisons. All analyses were performed using the SPSS software version 23.0 (SPSS Inc., Chicago, Illinois) and GraphPad Prism (version 8.2.1 Windows version, GraphPad Software, San Diego).

## Results

### The Basic Characteristics of Patients and Their Relationships With Prognosis

A total of 105 patients with newly diagnosed T-ALL were enrolled in this study. Among them, 76 were boys, with a median age of 8.9 (1.0-15.0) years. Based on the original CCCG-ALL-2015 protocol, 102 patients were classified as the IR group while the other three were in the HR group. The clinical demographics of the 105 children are shown in **Table 1**. Besides age and gender, clinical features, such as initial WBC, initial leukemia blasts in the PB, and karyotype, showed possible influences on the prognosis of the children (**Table 1**).

**Table 1:** The clinical-biological characteristics of 105 children with T-ALL and the comparisons of their survival.

Characteristics	N (%)	5-year OS (SE)	P^*^ value	5-year EFS (SE)	*P^#^* value	5-year DFS (SE)	*P^&^* value
**Total**	105 (100.0)	83.1 (4.8)		72.4 (5.6)		78.3 (4.6)	
**Gender**			**0.019**		**0.015**		0.085
Male	76 (72.4)	87.2 (5.7)		77.5 (7.0)		82.0 (5.2)	
Female	29 (27.6)	71.0 (9.4)		57.7 (10.0)		68.0 (9.5)	
**Age (years)**			**0.036**		0.101		**0.046**
<3	9 (8.6)	64.8 (16.5)		53.3 (17.3)		53.3 (17.3)	
≥3	96 (91.4)	85.4 (4.8)		74.8 (5.7)		81.1 (4.6)	
Median (range)	8.9 (1.0-15.0)						
**Initial WBC (×10^9^/L)**			0.100		**0.045**		**0.013**
<50	38 (36.2)	86.2 (10.5)		86.2 (6.8)		91.2 (6.2)	
≥50	66 (62.9)	80.3 (5.5)		66.0 (7.5)		71.9 (6.1)	
Unknown	1 (0.9)						
Median (range)	153.4 (0.3-800.7)						
**Initial Hemoglobin (g/L)**			0.878		0.088		0.055
<100	51 (48.6)	83.6 (5.9)		63.3 (9.0)		71.4 (6.8)	
≥100	53 (50.5)	83.2 (7.4)		84.2 (5.9)		87.7 (5.6)	
Unknown	1 (0.9)						
Median (range)	98.6 (39.0-152.0)						
**Initial Platelet (×10^9^/L)**			0.146		0.103		0.084
<20	6 (5.7)	100.0 (0.0)		83.3 (15.2)		83.3 (15.2)	
≥20 and <100	70 (66.7)	77.1 (6.6)		66.3 (7.3)		72.4 (6.1)	
≥100	28 (26.7)	95.7 (4.3)		91.1 (6.2)		96.3 (3.6)	
Unknown	1 (0.9)						
Median (range)	87.2 (4.0-357.0)						
**Initial blasts in BM (%)**			0.180		0.314		0.117
<50	10 (9.5)	100.0 (0.0)		90.0 (9.5)		90.0 (9.5)	
≥50 and <80	22 (21.0)	94.7 (5.1)		85.1 (8.0)		94.1 (5.7)	
≥80	73 (69.5)	77.7 (6.5)		66.1 (7.6)		72.2 (6.0)	
Median (range)	80.5 (30.0-99.5)						
**Initial blasts in PB (%)**			**0.004**		**0.011**		**0.005**
<20	24 (22.9)	100.0 (0.0)		91.5 (5.8)		100.0 (0.0)	
≥20 and <80	37 (35.2)	86.2 (10.5)		73.6 (11.9)		81.0 (8.0)	
≥80	43 (41.0)	77.7 (6.5)		62.7 (7.7)		66.5 (7.7)	
Unknown	1 (0.9)						
Median (range)	57.6 (0.0-99.0)						
**CNS involvement**			0.601		0.286		0.801
CNS1	89 (84.8)	84.8 (4.4)		73.5 (6.1)		78.7 (4.9)	
CNS2/3	12 (11.4)	75.0 (21.7)		66.8 (11.2)		76.4 (15.5)	
Unknown	4 (3.8)						
**Mediastinal mass**			0.938		0.281		0.786
Present	51 (48.6)	86.4 (4.8)		80.4 (5.6)		82.0 (5.4)	
Absent	52 (49.5)	76.0 (10.2)		59.0 (12.7)		71.6 (8.5)	
Unknown	2 (1.9)						
**Hepatomegaly**			0.555		0.201		0.112
Absent	53 (50.5)	75.0 (5.2)		72.5 (7.2)		75.6 (7.2)	
Mild (less than 5cm under the ribs)	48 (45.7)	91.3 (4.2)		76.2 (7.7)		83.2 (6.0)	
Severe (more than or equal to 5cm under the ribs)	3 (2.9)	66.7 (27.2)		66.7 (27.2)		66.7 (27.2)	
Unknown	1 (0.9)						
**Splenomegaly**			0.879		0.251		0.643
Absent	45 (42.9)	84.7 (5.9)		84.7 (5.9)		84.7 (5.9)	
Mild (less than 5cm under the ribs)	19 (18.1)	70.0 (17.0)		49.1 (17.4)		61.2 (16.1)	
Severe (more than or equal to 5cm under the ribs)	40 (38.1)	86.3 (5.7)		73.6 (7.3)		78.9 (6.7)	
Unknown	1 (0.9)						
**Immunophenotype**			0.212		0.555		0.800
ETP-ALL	26 (24.8)	77.1 (10.8)		66.7 (9.8)		73.0 (9.7)	
Early non-ETP-ALL	33 (31.4)	92.6 (5.2)		69.9 (11.8)		78.8 (8.7)	
Cortex T-ALL	18 (17.1)	94.4 (5.4)		88.5 (7.6)		88.5 (7.6)	
Medullary T-ALL	26 (24.8)	75.6 (8.7)		76.0 (8.5)		80.0 (8.0)	
Unknown	2 (1.9)						
**SIL-TAL1 translocation**			0.536		0.565		0.537
Present	22 (21.0)	89.8 (6.9)		79.3 (9.3)		83.1 (8.9)	
Absent	83 (79.0)	80.5 (6.3)		68.5 (7.7)		76.6 (5.4)	
**Myc positive**			0.336		0.819		0.691
Present	4 (3.8)	75.0 (21.7)		75.0 (21.7)		75.0 (21.7)	
Absent	101 (96.2)	83.7 (4.8)		72.6 (5.7)		78.6 (4.6)	
**MLL rearrangement**			0.416		0.274		0.308
Present	4 (3.8)	100.0 (0.0)		100.0 (0.0)		100.0 (0.0)	
Absent	101 (96.2)	84.2 (5.0)		71.3 (5.7)		77.3 (4.8)	
**CDKN2A/CEP9**			0.607		0.643		0.631
Present	34 (32.4)	89.5 (5.8)		56.0 (23.3)		80.7 (8.3)	
Absent	71 (67.6)	81.2 (5.9)		73.0 (5.8)		77.1 (5.9)	
**WT1 positive**			0.934		0.170		0.402
Present	36 (21.0)	79.3 (9.4)		72.1 (12.5)		80.7 (7.2)	
Absent	69 (79.0)	86.8 (4.4)		72.0 (6.0)		78.8 (5.3)	
**karyotype**			0.498		**0.042**		**0.038**
Normal	60 (57.1)	87.8 (4.8)		78.2 (7.6)		85.4 (5.2)	
Numerical abnormal	11 (10.5)	88.9 (10.5)		71.6 (14.0)		78.8 (13.4)	
Structure abnormal	25 (23.8)	65.6 (19.6)		53.0 (14.2)		56.3 (14.7)	
Failure or Missing	9 (8.6)						

T-ALL, T-cell acute lymphoblastic leukemia; WBC, white blood cells; BM, bone marrow; PB, peripheral blood; CNS, central nervous system; ETP, early T-cell precursor; ^*^significant differences about 5-year OS; ^#^significant differences about 5-year EFS; ^&^significant differences about 5-year DFS; Kaplan-Meier method was used to analyze the survival of each group and the differences between subgroups were evaluated using the log-rank test. Bold values indicate statistical significance at p < 0.05.

Of the 105 children with T-ALL, the karyotype could be evaluated for 96. Among them, 60 children had normal karyotypes and 36 had abnormal karyotypes, mainly in chromosomes 1, 2, 4, 5, 6, 7, 8, 9, 10, 11, 12, 13, 14, 17, 19, 21, X, and Y. Chromosomes 9 and 12 had the highest frequency of abnormality detection, with nine cases each, followed by chromosome 11 (7 cases), chromosome 14 (6 cases), chromosome 7 (6 cases), chromosome 4 (5 cases), chromosome 8 (5 cases), chromosome 6 (4 cases), chromosome 17 (4 cases), and chromosome X (3 cases). The frequency of detection for other chromosomal abnormalities was less than three cases. Four cases were hyperdiploid and seven cases were hypodiploid (**Supplementary Table 6**).

### Treatment Response and Outcomes

#### Overall Efficacy

**Figure 2:**
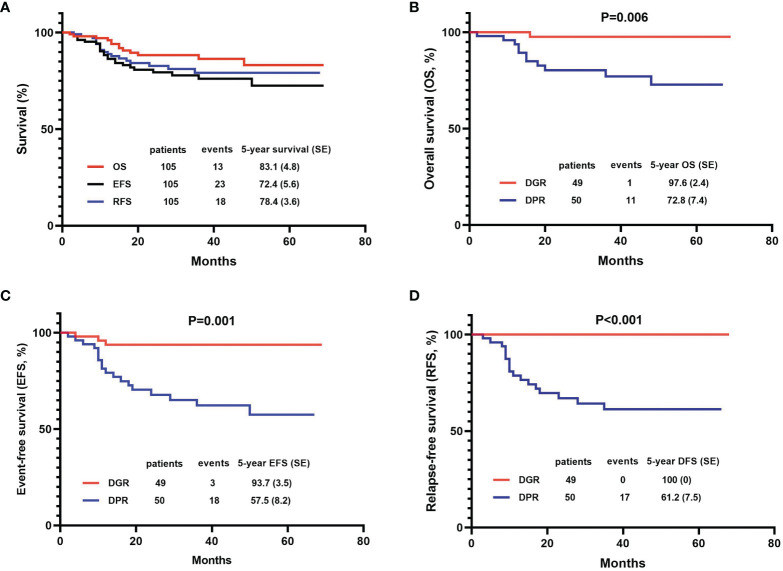
Efficacy of the 105 patients with T-cell acute lymphoblastic leukemia (T-ALL) using the Kaplan-Meier method. **(A)** OS, EFS, and DFS of total patients; **(B–D)** Comparisons between dexamethasone good responder (DGR) group and dexamethasone poor responder (DPR) group in OS, EFS, and DFS.

In this study, 103 (98.1%) patients achieved CR on day 33 and one patient exhibited induction resistance. The CR rate was 99.0% (104/105) (**Table 2**). Recurrence occurred in 18 out of 104 children (17.3%). The peak period of recurrence in children with T-ALL was within 2 years from the date of diagnosis, accounting for 88.9 (16/18) of all recurrence cases. Only two relapse events occurred in 2-3 years from the date of diagnosis, accounting for 11.1% (2/18) of all relapsed children. No recurrence was reported after 3 years from the date of diagnosis. The majority of recurrences were in the BM alone, accounting for 11 out of 18 (68.8%). The CNS alone was involved in four cases whereas both the BM and CNS were involved in the other three. Besides the BM and CNS, no recurrence was found in other parts of the body (**Table 2**). A total of 13 patients died, resulting in a mortality rate of 12.4% (13/105). Two years from the date of illness was the peak period of death in children with T-ALL. Leukemia progression in the refractory stage or relapse was the main cause of death (11/13, 84.6%), followed by infection (7.7%, 1/13) and secondary tumor (7.7%, 1/13). No deaths occurred due to the side effects of chemotherapy or bleeding from important organs (**Table 2**). Kaplan–Meier analysis demonstrated that the 5-year OS, EFS, and RFS rates for 105 patients were 83.1 ± 4.8%, 72.4 ± 5.6%, and 78.4 ± 3.6%, respectively, with a median follow-up of 36 (2-72) months (**Figure 2A**).

**Table 2:** Efficacy evaluation of 105 children with T-ALL.

Treatment outcome	All (%)	Risk group	
		IR (%)	HR (%)
Total	105 (100.0)	102 (100.0)	3 (100.0)
Remission	104 (99.0)	102 (100.0)	2 (66.7)
Induction remission	103 (98.1)	102 (100.0)	1 (33.3)
Continuous complete remission	86 (81.9)	86 (84.3)	0 (0.0)
Resistant	1 (0.9)	0 (0.0)	1 (33.3)
Relapse	18 (17.1)	17 (16.7)	1 (33.3)
Classification by time			
Within 1 year after diagnosis	10 (9.5)	10 (9.8)	1 (33.3)
Within 1-2 years after diagnosis	6 (5.7)	6 (5.9)	0 (0.0)
Within 2-3 years after diagnosis	2 (1.9)	2 (2.0)	0 (0.0)
More than 3 years after diagnosis	0 (0.0)	0 (0.0)	0 (0.0)
Classification by site			
BM	11 (10.5)	10 (9.8)	1 (33.3)
CNS	4 (3.8)	4 (3.9)	0 (0.0)
TEST	0 (0.0)	0 (0.0)	0 (0.0)
BM+CNS	3 (2.9)	3 (2.9)	0 (0.0)
BM+TEST or other extramedullary	0 (0.0)	0 (0.0)	0 (0.0)
HSCT (not because of refractory disease or relapse)	2 (1.9)	0 (0.0)	2 (66.7)
Withdrawal due to economic reasons	1 (0.9)	1 (0.9)	0 (0.0)
Death	13 (12.4)	12 (11.8)	1 (33.3)
Classification by time			
Within 1 year after diagnosis	3 (2.9)	2 (2.0)	1 (33.3)
Within 1-2 years after diagnosis	8 (7.6)	8 (7.8)	0 (0.0)
Within 2-3 years after diagnosis	0 (0.0)	0 (0.0)	0 (0.0)
More than 3 years after diagnosis	2 (1.9)	2 (2.0)	0 (0.0)
Classification by cause			
Infection	1 (0.9)	1 (1.0)	0 (0.0)
Bleeding	0 (0.0)	0 (0.0)	0 (0.0)
Chemotoxicity	0 (0.0)	0 (0.0)	0 (0.0)
Leukemia progression	11 (10.5)	10 (9.8)	1 (33.3)
Second tumor	1 (0.9)	1 (1.0)	0 (0.0)

T-ALL, T-cell acute lymphoblastic leukemia; IR, intermediate-risk; HR, high-risk; BM, bone marrow; CNS, central nervous system.

#### Comparison of the Efficacy in Children With Different Levels of Dexamethasone Sensitivity

Dexamethasone sensitivity was evaluated in 99 patients, of whom 49 (49.5%) were DGRs and 50 (50.5%) were DPRs. The 5-year OS, EFS, and RFS of patients in the DGR group were significantly higher than those in the DPR group (OS: 97.6 ± 2.4% *vs.* 72.8 ± 7.4%, P=0.006; EFS: 93.7 ± 3.5% *vs.* 57.5 ± 8.2%, P=0.001; RFS: 100.00% *vs.* 61.2 ± 7.5%, P<0.001) (**Figures 2B–D**).

#### Comparison of the Efficacy in Children With Different Levels of MRD

**Figure 3:**
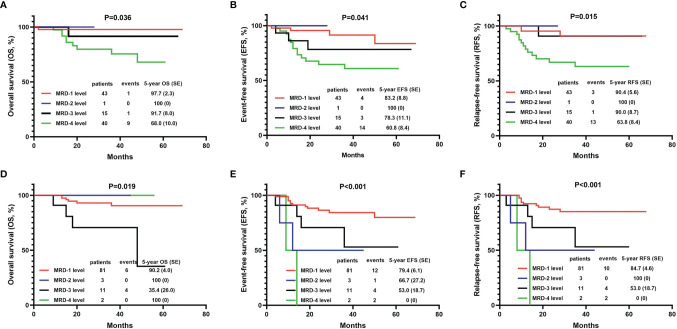
The 5-year OS, EFS, and DFS of T-ALL children with different grades of minimal residual disease (MRD). **(A)** Comparison of 5-year OS with different MRD grades on the 19th day of induction therapy. **(B)** Comparison of 5-year EFS with different MRD grades on the 19th day of induction therapy. **(C)** Comparison of 5-year DFS with different MRD grades on the 19th day of induction therapy. **(D)** Comparison of 5-year OS with different MRD grades on the 46th day of induction therapy. **(E)** Comparison of 5-year EFS with different MRD grades on the 46th day of induction therapy. **(F)** Comparison of 5-year DFS with different MRD grades on the 46th day of induction therapy. MRD-1 level, MRD<10^-4^; MRD-2 level, 10^-4^≤MRD<10^-3^; MRD-3 level, 10^-3^≤MRD<10^-2^; MRD-4 level, MRD≥10^-2^.

The BM MRD was first evaluated on the 19th day of induction therapy. Among the 99 evaluable children, 43 (43.4%) patients were at the MRD-1 level, one (1%) was at the MRD-2 level, 15 (15.2%) were at the MRD-3 level, and 40 (40.4%) were at the MRD-4 level. The 5-year OS, EFS, and DFS of children in the MRD-4 level were significantly lower than the rates for children in other MRD levels (**Figures 3A–C**).

On the 46th day of induction therapy (end of induction therapy), the BM MRD of 97 children was evaluated again. The number of MRD-1 patients among them had increased to 81 (83.5%), three (3.1%) children were at the MRD-2 level, 11 (11.3%) were at the MRD-3 level, and only two (2.1%) remained at the MRD-4 level. The 5-year EFS and DFS of children in the “MRD-4 level” group were significantly lower than the rates for those in other MRD groups (**Figures 3E, F**). However, the 5-year OS rate of the “MRD-4 level” group was not the lowest. The rate for the “MRD-3 level” group was significantly lower than those for the other three groups (OS: 100% in “MRD-4 level” group, 35.4 ± 26.0% in “MRD-3 level” group, 100% in “MRD-2 level” group, and 90.2 ± 4.0% in “MRD-1 level” group, P=0.019) (**Figure 3D**).

#### Univariate Analysis of OS, EFS, and RFS in Children With T-ALL

Cox regression analysis showed that gender, age, initial WBC, initial blasts in BM, initial blasts in PB, karyotype, DR, MRD on day 19 and MRD on day 46 affected the prognosis (OS, EFS, or RFS) of children with T-ALL (P values < 0.05, **Table 3**).

**Table 3:** Univariate analysis of the relationship between various clinical indicators and survival of children with T-ALL.

Variables	Overall survival (OS)			Event-free survival (EFS)			Relapse-free survival (RFS)		
	OR	95%CI (OR)	*p*-value	OR	95%CI (OR)	*p*-value	OR	95%CI (OR)	p-value
Gender	3.378	1.134-10.066	**0.029**	2.692	1.186-6.110	**0.018**	1.901	0.735-4.914	0.185
Age	0.236	0.064-0.879	**0.031**	0.362	0.122-1.075	0.067	0.273	0.089-0.836	**0.023**
Initial WBC	1.003	1.000-1.005	**0.029**	1.002	1.000-1.004	0.052	1.003	1.001-1.005	**0.015**
Initial hemoglobin	1.001	0.979-1.024	0.924	0.986	0.969-1.003	0.106	0.987	0.968-1.005	0.161
Initial platelet	0.995	0.985-1.005	0.334	0.995	0.988-1.003	0.211	0.997	0.989-1.004	0.367
Initial blasts in BM	1.085	1.002-1.174	**0.045**	1.023	0.992-1.055	0.151	1.031	0.992-1.072	0.120
Initial blasts in PB	1.047	1.009-1.086	**0.015**	1.022	1.005-1.040	**0.013**	1.035	1.010-1.060	**0.006**
CNS involvement	1.104	0.529-2.306	0.792	1.015	0.553-1.862	0.963	1.050	0.543-2.029	0.885
Mediastinal mass	0.346	0.077-1.562	0.168	0.826	0.339-2.011	0.673	0.726	0.258-2.041	0.544
Hepatomegaly	0.774	0.385-1.553	0.471	0.886	0.534-1.469	0.638	0.857	0.481-1.525	0.600
Splenomegaly	1.020	0.564-1.846	0.947	1.460	0.917-2.325	0.110	1.201	0.726-1.988	0.475
Immunophenotyping	1.272	0.802-2.017	0.307	0.991	0.695-1.414	0.961	0.940	0.631-1.402	0.763
karyotype	1.408	0.877-2.261	0.157	1.424	1.000-2.027	**0.049**	1.463	0.984-2.176	0.060
MLL rearrangement	0.046	0.000-3440.858	0.591	0.046	0.000-204.132	0.472	0.046	0.000-489.014	0.515
CDKN2A/CEP9	0.704	0.193-2.566	0.595	0.795	0.313-2.021	0.630	0.840	0.299-2.357	0.740
Myc positive	2.609	0.333-20.464	0.362	1.250	0.167-9.330	0.828	1.593	0.211-12.046	0.652
WT1 positive	0.939	0.306-2.880	0.912	0.528	0.207-1.345	0.181	0.719	0.268-1.925	0.511
SIL-TAL1 translocation	0.651	1.144-2.954	0.578	0.725	0.245-2.143	0.560	0.725	0.210-2.507	0.611
Dexamethasone response	2.883	1.216-6.834	**0.016**	2.745	1.434-5.254	**0.002**	3.335	1.634-6.806	**0.001**
MRD on day 19	2.151	1.231-3.760	**0.007**	1.549	1.120-2.143	**0.008**	1.659	1.124-2.448	**0.011**
MRD on day 46	1.653	1.196-2.284	**0.002**	1.573	1.228-2.015	**0.000**	1.516	1.139-2.017	**0.004**

T-ALL, T-cell acute lymphoblastic leukemia; WBC, white blood cells; BM, bone marrow; PB, peripheral blood; CNS, central nervous system; MRD, minimal residual disease. Cox regression analysis was used to assess the relationship between various factors and prognosis. Bold values indicate statistical significance at p < 0.05.

#### Multivariate Analysis of OS, EFS, and DFS in Children With T-ALL

In order to prove that the variables were still independent of the prognosis for the MRD status, we used MRD and each additional feature to fit a multivariate Cox model. The results showed that variables which were statistically significant in univariate analysis, such as gender, initial WBC, initial blasts in BM, initial blasts in PB, karyotype, DR, MRD on day 19 and MRD on day 46, were still statistically significant (P values < 0.05), with the exception of age (P=0.096). The variables that did not show statistical differences in univariate analysis failed to show any significance in multivariate analysis as well (**Supplementary Table 7**).

### Risk Study

#### The Risk Group Defined in CCCG-ALL-2015 Protocol

According to the CCCG-ALL-2015 risk group classification standard, 102 (97.1%) children were in the IR group, and three (2.9%) children were in the HR group. Kaplan-Meier analysis demonstrated that the EFS and RFS rates of children in the IR group were significantly higher than the rates for those in the HR group (EFS: 74.7 ± 5.7% *vs.* 0%, P<0.001; RFS: 80.8 ± 4.5% *vs.* 0%, P<0.001). However, due to the sample size, there was no significant difference in the OS rate between the two groups (83.6 ± 4.9% ***vs***. 66.7 ± 27.2%, P=0.192).

#### Redefinition of Risk Group Based on This Study

**Figure 4:**
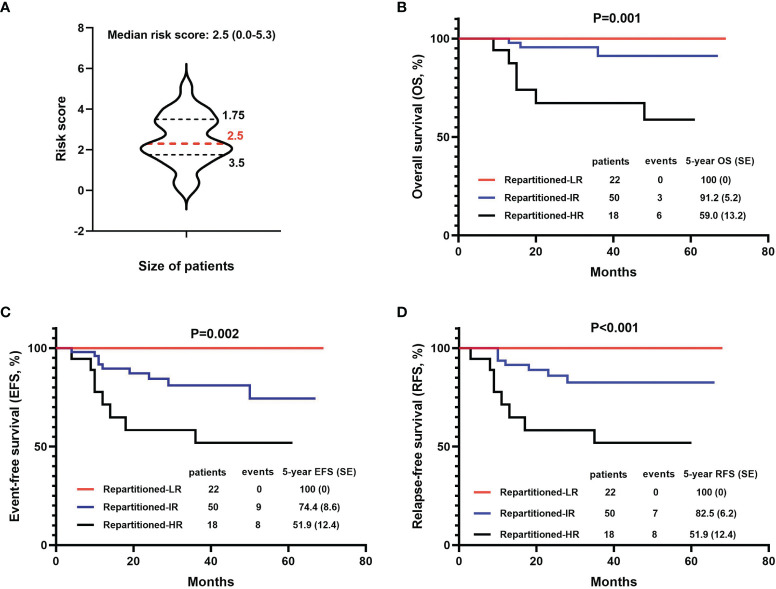
Comparison of survival (OS, EFS, and DFS) based on the new risk stratification systems. **(A)** The distribution of new risk scores in the patient cohort. **(B–D)** 5-year OS, EFS, and DFS based on 3 new levels of risk groups.

To better assess the therapeutic efficacy, we designed a new T-ALL risk group score calculation matrix which included nine clinical indicators significantly correlated to the outcome for children with T-ALL (OS, EFS, or DFS) in our analysis above (**Supplementary Table 4**). Based on this scoring system, 90 patients who had complete clinical data were reassessed. Among the risk scores for the 90 children, the minimum value was 0.0, the maximum value was 5.3, the upper quartile was 1.75, the median was 2.5, and the lower quartile was 3.5 (**Figure 4A**). Both univariate and multivariate analysis (Cox proportional hazards model) showed that the risk score had significant value for the prognosis (5-year OS, EFS, and RFS) of children with T-ALL (P values were all less than 0.05, **Supplementary Table 8**). According to the quartile of the risk score, the 90 children with T-ALL were divided into a low-risk group (22 children, risk score of 0-1.7), intermediate risk group (50 children, risk score of 1.8-3.5), and high-risk group (18 children, risk score of 3.6-5.3). The 5-year survival (OS, EFS, and RFS) rates for all patients in the low-risk group were 100%, significantly higher than the rates for those in the intermediate-risk group (91.2 ± 5.2%, 74.4 ± 8.6%, and 82.5 ± 6.2%, respectively), and high-risk group (59.0 ± 13.2%, 51.9 ± 12.4%, and 51.9 ± 12.4%, respectively) (all P values < 0.01) (**Figures 4B–D**).

### Targeted-NGS Results Analysis

**Figure 5:**
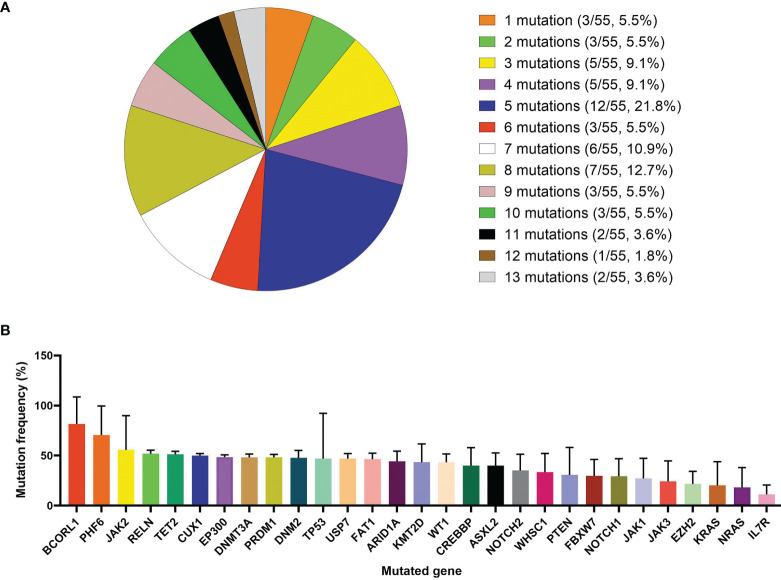
Detection of 112 types of hematological tumor-related gene mutations in 55 T-ALL children. **(A)** Detection of gene mutations in 55 T-ALL children with targeted next-generation sequencing (NGS) method. **(B)** Distribution of mutation frequency of genes with mutations detected three times or more.

In this study, 55 children randomly and voluntarily underwent targeted-NGS for 112 gene mutations that might be associated with blood malignant tumors and were named the “Targeted-NGS group”. These 55 patients harbored at least one gene mutation with a median of 5 (1–13) mutations per sample. Forty-nine patients (89.1%) had more than three mutations (**Figure 5A**). Total 345 mutations were found in 88 distinct genes with mutation loads ranging from 0.3% to 100%. The mutation frequencies of genes with more than three mutations are summarized in Fig. 5B. The most common mutated gene was *NOTCH1*, with a mutation rate of 63.7% (n = 37, the median mutation load was 29.32 (1.5-52.8) %), followed by *FBXW7*, *KMT2D*, *WT1*, *FAT1*, *CREBBP*, *RELN*, *PHF6*, *PTEN*, *JAK3*, and *DNM2* (mutated in >10% of the cases) [the specific mutation load of each gene is shown in (**Figure 5B**)]. Spearman’s correlation analysis showed that *EP300, PRDM1*, and *JAK1* mutations were correlated with the 19-day MRD level, and *EP300, JAK1*, and *DNMT3A* mutations were correlated with the 46-day MRD level (All P < 0.05, **Supplementary Table 9**). Kaplan-Meier analysis showed that children with *CREBBP*, *RELN*, *TP53*, *EP300*, *PRDM1*, or *JAK1* mutations had significantly lower 2-year OS, EFS, or RFS rates than those without mutations (All P < 0.05, **Supplementary Table 10**). Univariate analysis by Cox regression analysis showed that *CREBBP, RELN, TP53, EP300, PRDM1*, and *JAK1* mutations were related to the prognosis (5-year OS, EFS or RFS) of children with T-ALL (All P < 0.05, **Supplementary Table 11**). In order to confirm that the variables were still independent of the prognosis for the MRD status, we used MRD and each gene to fit a multivariate Cox model. The results showed that *FBXW7*, *CREBBP*, *RELN*, *PTEN*, *TP53*, *EP300*, *PRDM1*, and *JAK1* mutations were related to the prognosis (5-year OS, EFS, or RFS) of children with T-ALL (P values less than 0.05, **Supplementary Table 12**). The remaining 50 patients who did not undergo genetic examination were grouped into the “Non-Targeted-NGS group”. There was no statistical difference in the basic clinical demographics between these two groups (**Supplementary Table 13**).

In order to further explore the value of gene mutations in the prognosis of T-ALL, we divided the 88 mutant genes detected with NGS into 10 groups: 1) NOTCH signaling pathway, 2) Ras/Protein phosphatase/MARK/PI3K signaling pathway, 3) Transcription factor/regulation, 4) Epigenetic modulators, 5) Jak-Stat Signaling Pathway, 6) Splicing and mRNA processing regulation, 7) NF-KB pathway, 8) Wnt/β-Catenin pathway, 9) Receptor/Nonreceptor tyrosine kinase signaling pathway, and 10) Cyclins and Cell Cycle Regulation (19, 24–26). The genes included in each group are shown in **Supplementary Table 14**. Patients with “Ras/Protein phosphatase/MARK/PI3K signaling Pathway” abnormalities tended to have higher 2-year OS while those with “Transcription factor/regulation” abnormalities tended to have lower 2-year OS, EFS, and RFS (P values less than 0.05, **Supplementary Table 15**). Cox univariate analysis showed that abnormalities in “Transcription factor/regulation” could affect the EFS and RFS of patients (P values less than 0.05, **Supplementary Table 16**). Cox multivariate analysis showed that the effect of abnormal “Transcription factor/regulation” on RFS was not influenced by MRD. The “Jak-Stat Signaling Pathway” also affected the OS, EFS, and RFS of patients independent of MRD (P values less than 0.05, **Supplementary Table 17**). Further analysis of the effect of synergy between the 10 types of signaling pathways on the prognosis of T-ALL patients showed that Group 1 (combination of “NOTCH signaling pathway” and “Ras/Protein phosphatase/MARK/PI3K signaling pathway”), Group 2 (combination of “NOTCH signaling pathway” and “Transcription factor/regulation”), Group 3 (combination of “NOTCH signaling pathway” and “Epigenetic modulators”); and Group 18 (combination of “Transcription factor/regulation” and “Epigenetic modulators”) had an impact on patient outcomes (OS, EFS, or RFS) (P values less than 0.05, **Supplementary Tables 18 and 19**). Furthermore, the effects of Group 2 and Group 3 on patient outcomes were independent of MRD (P < 0.05 for both, **Supplementary Table 20**). We conducted an in-depth analysis of the four associated signaling pathway systems (NOTCH signaling pathway, Transcription factor/regulation, Epigenetic modulators, and Ras/Protein phosphatase/MARK/PI3K signaling pathway) that had an impact on the prognosis. We found that the NOTCH signaling pathway combined with CREBBP (**Supplementary Tables 21–23**), RELN (**Supplementary Tables 24–26**), or KMT2D (**Supplementary Table 26**), as well as a combination of CREBBP and KMT2D (**Supplementary Table 32**) could affect patient outcomes (OS, EFS, or RFS). However, other gene combinations in these four signaling pathways did not show statistical value (**Supplementary Tables 27**–**31**).

## Discussion

To our knowledge, this is the first report on the detailed treatment efficacy of this program on T-ALL (14). Due to the lack of an independent T-ALL treatment program in China, the disease shared a set of risk groups and treatment programs with B-ALL for several decades, including the current study program (CCCG-ALL-2015 program). However, due to the complex genetic background and poor prognosis of T-ALL, T-ALL patients could only be classified into an intermediate-risk group in many previous programs, and the efficacy of treatment was unsatisfactory. FC-MRD has been included in the risk classification standard since the 2015 program. The introduction of FC-MRD further refined the risk classification of T-ALL, and the treatment efficacy also improved to a certain extent. This study showed that the efficacy of the 2015 regimen for T-ALL was significantly higher than the results of previous domestic trials (6, 12), and slightly lower than the international advanced level (AALL0434 trial) (3). However, it was obvious that the CCCG-ALL-2015 program could not effectively distinguish children with T-ALL at different risk levels (0 cases at standard risk, 102 cases at intermediate risk, and 3 cases at high risk). Most children with T-ALL (102/105) remained in the intermediate-risk group, receiving the same treatment but with different outcomes. Additional clinical indicators, including molecular biology, may have profound value for the risk grouping of children with T-ALL but have been neglected. Therefore, a refined T-ALL risk scoring system (including multiple clinical indicators other than FC-MRD) was established in this study for more precise risk stratification. In addition to the analysis of traditional clinical indicators, 112 genes in 55 children with T-ALL were also analyzed in this study using targeted NGS, and the influence of complex genetic backgrounds on prognosis was explored in children with T-ALL.

Glucocorticoids (prednisone or dexamethasone) are important drugs in the treatment of ALL. Compared to prednisone, dexamethasone has a longer half-life and can better penetrate the CSF. However, long-term use of dexamethasone has a higher risk of infection and osteoporosis. Consequently, opinions on the use of prednisone or dexamethasone have been inconsistent for several years (27–29). The CCCG-ALL-2015 program proposed 4 days of dexamethasone as a pre-treatment strategy, which minimized potential side effects and was considered an improvement over the CCLG-ALL-2008 program. In our current CCCG-ALL-2020 project, we performed a randomized trial of dexamethasone and prednisone in the induction treatment phase for intermediate-risk patients to further evaluate the therapeutic efficacy as well as the side effects.

The improvement of the efficacy of the CCCG-ALL-2015 program for children with T-ALL was mainly due to the important role of MRD monitoring in the adjustment of risk groups and the adjustment of MRD monitoring points. In the CCLG-ALL-2008 program, we monitored some children with MRD and set three MRD monitoring points: days 15, 33, and 90 during induction therapy (before intensive treatment). After reviewing the CCLG-ALL-2008 program, we found that adjusting the risk group and the treatment of children with T-ALL according to the MRD level may improve the therapeutic efficacy. In previous conventional regimes, e.g., CCGL-ALL-2008, the time point of MRD monitoring at 90 days showed the most predictive value for the prognosis of children (12). However, in the CCCG-ALL-2015 plan, we focused on the principle of non-inferiority to reduce the intensity of chemotherapy as much as possible, introduced intensive treatment (CAM) on the 29th day of VDLP induction therapy, and reduced the two-CAM course (28 days in total) to one 7-day CAM. These improvements shortened the MRD detection time (after induction therapy + early intensive therapy) from 3 months to 46 days. MRD recording was also adjusted from the previous three to two times (on the 19^th^ and 46^th^ days in the induction treatment phase, which was the middle and end points of the induction treatment phase, respectively). Based on the MRD levels at these two time points, we performed strict risk group assessment and modified the follow-up treatments accordingly. The outcome of this study showed that this adjustment made the MRD more realistically reflect the residual status of leukemia cells in children with T-ALL, which is an important factor in the risk assessment of children with T-ALL.

The precise risk stratification and the subsequent treatment based on this stratification may be the key to break through the bottleneck of the curative effect for T-ALL in children. However, the risk classification system in the CCCG-ALL-2015 program requires improvement as it was designed for both B-ALL and T-ALL in children, rather than specifically for T-ALL. The risk grouping criteria do not fully reflect the clinical situation and thus are less applicable to children with T-ALL. Therefore, in this study, we proposed 22 biological indicators that may affect the prognosis of children with T-ALL based on our clinical observations, and nine of them were demonstrated to be useful. By grading according to these nine indicators, a new risk classification standard was developed. The 90 evaluable children were divided into LR, IR, and HR groups according to the new risk classification standard. Based on the new risk groups, we will be able to further refine our treatment strategy: for children in the LR group, we can try to reduce the intensity of chemotherapy as appropriate to further reduce the associated side effects and improve the children’s quality of life. For children in the HR group, we can adopt more aggressive treatment methods such as HSCT at earlier periods before any recurrence of the disease. For children in the IR group, we can extensively examine their genotypes and clinical features, and introduced potential targeted interventions to improve the outcomes.

Due to recent advances in NGS technology, genetic examinations have been extensively applied in both pediatric and adult ALL (30, 31). In this study, 112 hematological tumor-related gene mutations were explored using NGS in 55 children. Through analysis of the relationship between the mutant genes and the prognosis of children, it was found that three gene mutations (*EP300, PRDM1, and JAK1*) affected the MRD and six gene mutations (*CREBBP, RELN, TP53, EP300, PRDM1, and JAK1*) had an impact on the 2-year survival, indicating that MRD-associated genetic mutations do not always coincide with those that affect the OS and *vice versa*. Our results suggest that both gene mutation detection and MRD monitoring are important independent indicators for the prognosis of children with T-ALL and cannot be substituted for each other.

Although the karyotypes of many T-ALL-related abnormalities are unclear for technical reasons, the important value of G-banding cannot be denied, especially in hospitals where gene sequencing is not available. However, due to the complex genetic background of T-ALL, the number of cases for each chromosomal abnormality was less than 10. Our aim was to identify a strategy to reasonably introduce G-banding into the risk scoring system. In previous research (6) as well as in this study, the EFS and RFS of the “normal karyotype” were higher than those of the “numerical abnormal karyotype”, which were higher than those of the “structure abnormal karyotype” (EFS: 78.2 ± 7.6%, 71.6 ± 14.0%, 53.0 ± 14.2%, P = 0.042; RFS: 85.4 ± 5.2%, 78.8 ± 13.4% and 56.3 ± 14.7%, P = 0.038). We defined the “normal karyotype” as a score of 0, “numerical abnormality” as 0.3, and “structural abnormality” as 0.5. However, it is worth noting that in this scoring system, the “normal karyotype” does not indicate a favorable genotype, and structural changes do not necessarily mean “poor prognostic markers”. The introduction of G-banding was only a transitional stage of risk scoring, and its real impact also depends on the oncogene or tumor suppressor genes involved. The risk scoring system for the introduction of gene sequencing warrants further study. In addition, the two genes identified in this study, MYC and KMT2A (MLL), were only present in very few cases. We therefore simply presented the clinical features associated with the genes, but were unable to clarify their value in childhood T-ALL or include them in the scoring system. It is necessary to expand the number of cases for in-depth analysis.

Several limitations of this study should also be noted. Firstly, due to the low incidence of T-ALL and the limited number of cases in a single center, a verification group was not included in the present study. Additionally, because the scoring system was released recently, it has not been widely used in clinical practice. Secondly, although late recurrence of T-ALL is very rare, and our previous study found that all childhood T-ALL recurrences occurred within 36 months of diagnosis (unpublished), long-term follow-up may still be necessary in order to optimize the risk scoring model. Thirdly, although the initial exploration of the clinical value of numerous genetic mutations involved in T-ALL is one of the highlights of this study, it should be noted that T-ALL has a complex genetic background characterized by concomitant lesions that contribute to leukemogenesis. A large number of cases and more in-depth analyses such as PTEN deletions are required to assess their clinical impact. Therefore, a risk scoring system with the genetic background of T-ALL will be the research focus and challenge in the future. Heterogeneous treatment modalities based on precise risk stratification covering genetic backgrounds may be the future direction to further improve the efficacy of children with T-ALL.

## Data Availability Statement

The original contributions presented in the study are included in the article/**Supplementary Material**. Further inquiries can be directed to the corresponding author.

## Ethics Statement

The studies involving human participants were reviewed and approved by Ethics Committee of Blood Diseases Hospital, Chinese Academy of Medical Sciences; State Key Laboratory of Experimental Hematology, National Clinical Research Center for Blood Diseases, Haihe Laboratory of Cell Ecosystem, Institute of Hematology & Blood Diseases Hospital, Chinese Academy of Medical Sciences & Peking Union Medical College. Written informed consent to participate in this study was provided by the participants’ legal guardian/next of kin.

## Author Contributions

XZ and XL contributed to the design of the study. XL, YZ, LZ, YG, YC, WY, XC, SW, MR, LC, XYZ, BZ, and RZ enrolled and treated all patients. XL, AZ, LL, LYZ, and MY collected clinical data. XL and YCZ analyzed the data. XL wrote the manuscript. All authors contributed to the article and approved the submitted version.

## Conflict of Interest

The authors declare that the research was conducted in the absence of any commercial or financial relationships that could be construed as a potential conflict of interest.

## Publisher’s Note

All claims expressed in this article are solely those of the authors and do not necessarily represent those of their affiliated organizations, or those of the publisher, the editors and the reviewers. Any product that may be evaluated in this article, or claim that may be made by its manufacturer, is not guaranteed or endorsed by the publisher.

## Funding

This work was supported by awards from the Natural Science Foundation Project: 81870131 and 81770175.
