# Supplementary material for: A Novel Risk Defining System for Pediatric T-Cell Acute Lymphoblastic Leukemia From CCCG-ALL-2015 Group
Source: Front Oncol. 2022 Feb 28;12:841179. doi: 10.3389/fonc.2022.841179 (PMC8920043; doi:10.3389/fonc.2022.841179)
Supplement: Supplementary file 1 [file Table_1.docx]

Supplementary Table 1: Antibody combination panel for T-cell acute lymphoblastic leukemia (T-ALL) detection.

|  | FITC | PE | PC5.5 | PC7 | APC | APC-H7 | V450 | V500 |
| --- | --- | --- | --- | --- | --- | --- | --- | --- |
| 1 | CD15 | CD117 | CD34 | CD56 | CD33 | HLA-DR | CD38 | CD45 |
| 2 | CD64 | CD7 | CD34 | CD123 | CD13 | HLA-DR | CD11B | CD45 |
| 3 | CD36 | CD10 | CD5 | CD20+CD2 |  | CD14 | CD19 | CD45 |
| 4 | TDT | MPO |  | cCD3 | cCD79a |  |  | CD45 |
| 5 | CD8 | CD1a | CD3 |  | CD4 | CD2 |  | CD45 |
