# Supplementary material for: A Novel Risk Defining System for Pediatric T-Cell Acute Lymphoblastic Leukemia From CCCG-ALL-2015 Group
Source: Front Oncol. 2022 Feb 28;12:841179. doi: 10.3389/fonc.2022.841179 (PMC8920043; doi:10.3389/fonc.2022.841179)
Supplement: Supplementary file 2 [file Table_2.docx]

Supplementary Table 2: Antibody combination panel for minimal residual disease (MRD) detection.

|  | FITC | PE | PC5.5 | PC7 | APC | APC-H7 | V450 | V500 |
| --- | --- | --- | --- | --- | --- | --- | --- | --- |
| **tube1** | TDT | CD2 | CD34 | CD117+CD33 | CD7 | CD10 | cCD3 | CD45 |
| **tube1** | CD4 | CD99 | CD5 | CD3 | CD7 | CD8 | CD16+CD56 | CD45 |
