# Supplementary material for: A Novel Risk Defining System for Pediatric T-Cell Acute Lymphoblastic Leukemia From CCCG-ALL-2015 Group
Source: Front Oncol. 2022 Feb 28;12:841179. doi: 10.3389/fonc.2022.841179 (PMC8920043; doi:10.3389/fonc.2022.841179)
Supplement: Supplementary file 3 [file Table_3.docx]

Supplementary Table 3: 112 genes covered by A custom targeted next-generation sequencing (NGS) gene panel

| ABL1 | AKT1 | ALAS2 | ASXL1 | ARID1A | ATM | ABCB1 | ADAMTS13 | BIRC3 | BCL2 | BCL6 | BRAF |
| --- | --- | --- | --- | --- | --- | --- | --- | --- | --- | --- | --- |
| CALR | CCND1 | CBL | CCND3 | CDKN1A | CEBPA | CREBBP | CSF3R | CUX1 | CYLD | CRLF2 | DDX3X |
| DNM2 | DNMT3A | DIS3 | EGFR | EP300 | EPHA7 | ETV6 | EZH2 | EED | ECT2L | FANCA | FANCC |
| FANCG | FAT1 | FGFR3 | FLT3 | FBXW7 | FAM46C | GATA2 | GATA3 | IDH1 | IDH2 | IL7R | ITK |
| IKZF1 | JAK1 | JAK2 | JAK3 | KMT2D | KIT | KRAS | LYST | MUM1 | MAF | MPL | MYC |
| MYD88 | MYH11 | MAPK1 | MAFB | NOTCH1 | NOTCH2 | NPM1 | NRAS | NF1 | PRDM1 | PDGFRB | PIK3CA |
| PRF1 | PTEN | PTPN11 | PAX5 | PRMT5 | PRPF40B | PHF6 | RUNX1 | RELN | RAB27A | RB1 | SH2D1A |
| SF1 | SH2B3 | SF3A1 | STX11 | SF3B1 | SUZ12 | SMC1A | SMC3 | SRSF2 | STXBP2 | SF1 | SAMHD1 |
| SETBP1 | TET2 | TAL1 | TNFAIP3 | TP53 | TRAF3 | U2AF1 | U2AF2 | UNC13D | WAS | WHSC1 | WT1 |
| XIAP | XPO1 | ZRSR2 | ZMYM3 |  |  |  |  |  |  |  |  |
