# Supplementary material for: A Novel Risk Defining System for Pediatric T-Cell Acute Lymphoblastic Leukemia From CCCG-ALL-2015 Group
Source: Front Oncol. 2022 Feb 28;12:841179. doi: 10.3389/fonc.2022.841179 (PMC8920043; doi:10.3389/fonc.2022.841179)
Supplement: Supplementary file 4 [file Table_4.docx]

Supplementary Table 4. The risk score includes 9 weighted indicators

| Characteristics | Score |
| --- | --- |
| Gender |  |
| Male | 0 |
| Female | 0.5 |
| Age (years) |  |
| <3 | 0.5 |
| ≥3 | 0 |
| Initial WBC (×10^9^/L) |  |
| <50 | 0 |
| ≥50 | 0.5 |
| Initial blasts in BM (%) |  |
| <50 | 0 |
| ≥50 and <80 | 0.3 |
| ≥80 | 0.5 |
| Initial blasts in PB (%) |  |
| <20 | 0 |
| ≥20 and <80 | 0.3 |
| ≥80 | 0.5 |
| karyotype |  |
| Normal | 0 |
| Numerical abnormal | 0.3 |
| Structure abnormal | 0.5 |
| Dexamethasone response |  |
| DGR | 0 |
| DPR | 0.5 |
| MRD at day 19 |  |
| MRD<0.0001 | 0 |
| 0.0001≤MRD<0.001 | 0.5 |
| 0.001≤MRD<0.01 | 1 |
| MRD≥0.01 | 1.5 |
| MRD at day 46 |  |
| MRD<0.0001 | 0 |
| 0.0001≤MRD<0.001 | 0.5 |
| 0.001≤MRD<0.01 | 1 |
| MRD≥0.01 | 1.5 |

WBC, white blood cells; PB, peripheral blood; DGR, dexamethasone good responder; DPR, dexamethasone poor responder; MRD, minimal residual disease.

Selection and scoring rules: for any variables (Kaplan-Meier method or Cox proportional hazards model) with a P value less than 0.05, the risk side score was 0.5 (each MRD has four levels, and the risk score increases by 0.5 for each level increase.
