# Supplementary material for: A Novel Risk Defining System for Pediatric T-Cell Acute Lymphoblastic Leukemia From CCCG-ALL-2015 Group
Source: Front Oncol. 2022 Feb 28;12:841179. doi: 10.3389/fonc.2022.841179 (PMC8920043; doi:10.3389/fonc.2022.841179)
Supplement: Supplementary file 5 [file Table_5.docx]

Supplementary Table 5. CCCG-ALL-2015 Treatment Protocol in T-ALL.

| Drugs and route of administration | Dosage and frequency of administration | | Days of Administration |
| --- | --- | --- | --- |
| **Induction treatment (IR/HR)**  Protocol VDLP |  | |  |
| Dexamethasone (PO/IV) | 6 mg/m^2^.d bid | | 1-4 |
| Prednisone (PO) | 60 mg/m^2^.d tid | | 5-28, then taper over 9 days |
| Vincristine (IV) | 1.5 mg/m^2^ qw (max. 2 mg) | | 5, 12, 19, 26 |
| Daunorubicin (IV gtt) | 25 mg/m^2^ qw | | 5, 12 |
| Peg-Asp (IM) | 2000 U/m^2^ | | 6, 26 |
| IT, Methotrexate + Cytarabine + steroid | Doses according to age | | 5, 8, 12, 15, 19 |
| Protocol CAM^a^ |  | |  |
| Cyclophosphamide (IV gtt) | 1000 mg/m^2^ | | 29 |
| Cytarabine (IH) | 50 mg/m^2^ q12h | | 29-35 |
| 6-mercaptopurine (PO) | 60 mg/m^2^.d qn | | 29-35 |
| IT, Methotrexate + Cytarabine + steroid | Doses according to age | | 29 |
| Protocol VLCAM |  | |  |
| Vincristine (IV) | 1.5 mg/m^2^ qw (max. 2 mg) | | 50, 57 |
| Peg-Asp (IM) | 2000 U/m^2^ | | 50 |
| Cyclophosphamide (IV gtt) | 1000 mg/m^2^ | | 50 |
| Cytarabine (IH) | 50 mg/m^2^ q12h | | 50-56 |
| 6-mercaptopurine (PO) | 60 mg/m^2^.d qn | | 50-56 |
| IT, Methotrexate + Cytarabine + steroid | Doses according to age | | 50 |
| **Consolidation treatment (IR/HR)** |  | |  |
| Protocol HD-MTX (four rounds every 2 weeks) |  | |  |
| 6-mercaptopurine (PO) | 25 mg/m^2^.d | | 1-56 |
| Methotrexate (IV gtt) ^f^ | 5 g/m^2^ | | 1, 15, 29, 43 |
| IT, Methotrexate + Cytarabine + steroid | Doses according to age | | 1, 15, 29, 43 |
| **Continue treatment (IR/HR)** | |  |  |
| Interval treatment | |  |  |
| Protocol VDLD+6-mp (21 days a cycle for 4 cycles and 28 days a cycle for 1 cycle) |  | |  |
| Vincristine (IV) | 1.5 mg/m^2^ qw (max. 2 mg) | | 1 |
| Doxorubicin (IV gtt) | 25 mg/m^2^ | | 1 |
| Peg-Asp (IM) | 2000 U/m^2^ | | 1 |
| Dexamethasone (PO/IV) | 12 mg/m^2^.d bid | | 1-5 |
| 6-mercaptopurine (PO)  IT, Methotrexate + Cytarabine + steroid | 25 mg/m^2^.d qn  Doses according to age | | 1-21/28  1 |
| Re-induction treatment | | | |
| Protocol VALD |  | |  |
| Vincristine (IV) | 1.5 mg/m^2^ qw (max. 2 mg) | | 1, 8, 15 |
| Cytarabine (IV gtt) | 2 g/m^2^ q12h | | 1-2 |
| Peg-Asp (IM) | 2000 U/m^2^ | | 3 |
| Dexamethasone (PO/IV) | 8 mg/m^2^.d bid | | 1-7, 15-21 |
| IT, Methotrexate + Cytarabine + steroid | Doses according to age | | 1 |
| Maintenance treatment |  | |  |
| Protocol MM+CAVD (5 cycles) |  | |  |
| Methotrexate (PO) | 25 mg/m^2^.d qw | | 1,8 |
| 6-Mercaptopurine (PO) | 50 mg/m^2^.d qn | | 1-14 |
| Cyclophosphamide (IV gtt) | 300 mg/m^2^ | | 15 |
| Cytarabine (IV gtt) | 300 mg/m^2^ | | 15 |
| Vincristine (IV) | 1.5 mg/m^2^ (max. 2 mg) | | 15 |
| Dexamethasone (PO/IV) | 8 mg/m^2^.d | | 15-21 |
| IT, Methotrexate + Cytarabine + steroid | Doses according to age | | 15 |
| Protocol MM+CAVD/CA (random, 7 cycles) |  | |  |
| Methotrexate | 25 mg/m^2^.d qw | | 1, 8, 15, 21, 28, 35 |
| 6-Mercaptopurine | 50 mg/m^2^.d qn | | 1-42 |
| Cyclophosphamide (IV gtt) | 300 mg/m^2^ | | 43 |
| Cytarabine (IV gtt) | 300 mg/m^2^ | | 43 |
| Vincristine (IV) | 1.5 mg/m^2^ (max. 2 mg) | | 43 |
| Dexamethasone (PO/IV) | 6 mg/m^2^.d | | 43-50 |
| Protocol MM (2 cycles) |  | |  |
| Methotrexate | 25 mg/m^2^.d qw | | 1, 8, 15, 21, 28, 35, 42, 49 |
| 6-Mercaptopurine | 50 mg/m^2^.d qn | | 1-56 |
| **Pre-transplant treatment (HR)^b^** |  | |  |
| Protocol DAEL |  | |  |
| Dexamethasone (PO/IV) | 20 mg/m^2^.d bid | | 1-6 |
| Cytarabine (IV gtt) | 2 g/m^2^ q12h | | 1-2 |
| VP-16 (IV gtt) | 100 mg/m^2^ q12h | | 3-5 |
| Peg-Asp (IM) | 2500 U/m^2^ | | 6 |
| IT, Methotrexate + Cytarabine + steroid | Doses according to age | | 5 |

^a^ If WBC>4.0×10^9^/L and ANC>1.0×10^9^/L, CAM can be given in advance to d27. If WBC<2.0×10^9^/L or ANC<0.8×10^9^/L, CAM can be given after d33. If d33, WBC and ANC are still lower than this value, 6-MP and Ara-C can be given by half.

^b^ The number of DAELs is determined by the MRD after the course of treatment.

Abbreviations: IR, intermediate-risk; HR, high-risk; PO, by mouth; IV, intravenous push; IV gtt, intravenous drip; IT, intrathecal; bid, twice a day; tid, three times a day; qn, once a night; qw, once a week; q12h, once every 12 hours.
