# Supplementary material for: A Novel Risk Defining System for Pediatric T-Cell Acute Lymphoblastic Leukemia From CCCG-ALL-2015 Group
Source: Front Oncol. 2022 Feb 28;12:841179. doi: 10.3389/fonc.2022.841179 (PMC8920043; doi:10.3389/fonc.2022.841179)
Supplement: Supplementary file 6 [file Table_6.docx]

**Supplementary Table 6. The specific content of 36 cases of abnormal karyotype**

| **Patient No.** | **karyotype*** |
| --- | --- |
| **1** | 46, X,-Y,?del(9)(p13),der(11)t(11;14)(p14;q21),?dup(17)(q21q25),+21,+2mar[5] |
| **2** | 46, XY,?t(11;14)(p15;q11)[5]/46,XY[3] |
| **3** | 46, XY,t(8;9)(p22;p21)[18]/46,XY[2] |
| **4** | 46,XY,?del(9)(p21)[1]/46,XY[3] |
| **5** | 46, XY,del(4)(p14),t(5;7)(q32;q36)[5]/46,XY[3] |
| **6** | 46, XY,-4,+der(12)t(4;12)(q12;p13),?der(17)[1]/45,XY,-4,-4,der(12)t(4;12)(q12;p13)+mar[1]/46,XY[3] |
| **7** | 46, XY,del(11)(q23)[10]/46,XY[10] |
| **8** | 46, XY,del(9)(p13p22)[1]/46,XY[3] |
| **9** | 46, XX，der(6)t(1;6)(q31;q22),?add(12)(24)[5]/46,XX[15] |
| **10** | 46, XX,der(11)t(11;14)(p13;q11.2),-14,+21[3]/46，XX[17] |
| **11** | 46, XY,del(7)(?q11)[1]/46,XY[19] |
| **12** | 46, XX,t(1;14;12)(q12;q11.2;p11.2)[18]/46,XX[2] |
| **13** | 46, XY,del(6)(q21)[20] |
| **14** | 46, XY,der(5),-6,+mar[20] |
| **15** | 46, XY,add(9)(p13),add(9)(p13)[10]/46,XY[10] |
| **16** | 46, XX,t(6;11)(q27;q23)[1]/46,XX[19] |
| **17** | 46, X,del(X)(q11)[2]/48,idem,add(1)(p36.1),del(1)(p32),+del(1)(q42),+del(6)(q13q23),-11,+12,inc[3]/46 |
| **18** | 46, XY,-1,-13,-14,-19,+4mar,inc[2]/46,XY[4] |
| **19** | 46, XX,t(7;12)(p15;q13)[11]/46,XX[9] |
| **20** | 46, XX,t(7;12)(p15;q13)[13]/46,XX[9] |
| **21** | 46, XY,-2，-16,+2mar,inc[18]/46,XY[2] |
| **22** | 46, XY,?t(6:8)(p21:q24),der(10)[cp7]/46,XY[13] |
| **23** | 46, X,t(X;17)(q13;p11.2)[16] |
| **24** | 46, XY,add(12)(p11.2)[1]/46,XY[8] |
| **25** | 46, XY,9qh+[20] |
| **26** | 92, XXYY,-8,-8,add(12)(p12),add(12)(p12),+21,+21,+3mar,inc[cp5]/46,xy,del(9)(q22),der(16)[1]/46,xy,de |
| **27** | 47, XY,?+,19[3]/46,XY[1] |
| **28** | 44, XY,-7,-16[1] |
| **29** | 45, XY,?der(9;15)(q13;q10)[2]/46,XY[11] |
| **30** | 45, XY,-17[1] |
| **31** | 45, XX，-4，？del（9）（p21）-10，-11，-14，+3mar，inc[9]/46，XX[4] |
| **32** | 45, XX,-?4,?del(4)(q31),inc[6]/46,xx[14] |
| **33** | 47, XX,+?8[10]/46,XX[10] |
| **34** | 44, XY,?der(7),-8,-9,-10,der(12),+mar,inc[cp3]/46,XY,t(9;11)(p22;q23)[2]/46,XY[15] |
| **35** | 47, XX,+8[5]/46，XX[15] |
| **36** | 45, XY,del(5)(q31),add(12)(p13),-?14[7] |
