# Supplementary material for: A Novel Risk Defining System for Pediatric T-Cell Acute Lymphoblastic Leukemia From CCCG-ALL-2015 Group
Source: Front Oncol. 2022 Feb 28;12:841179. doi: 10.3389/fonc.2022.841179 (PMC8920043; doi:10.3389/fonc.2022.841179)
Supplement: Supplementary file 7 [file Table_7.docx]

**Supplementary Table 7. Multivariate analysis of the relationship between various clinical indicators and survival of children with T-ALL.**

| Variables | Overall survival (OS) | | | |  | Event-free survival (EFS) | | |  | Relapse-free survival (RFS) | | |
| --- | --- | --- | --- | --- | --- | --- | --- | --- | --- | --- | --- | --- |
|  | OR | 95%CI (OR) | | *p*-value |  | OR | 95%CI (OR) | *p*-value |  | OR | 95%CI (OR) | p-value |
| Gender | 2.624 | | 0.874-7.879 | 0.085 |  | 2.275 | 0.994-5.209 | 0.052 |  | 1.532 | 0.587-3.998 | 0.384 |
| MRD at day 19 | 2.063 | | 1.167-3.647 | **0.013** |  | 1.488 | 1.072-2.066 | **0.018** |  | 1.624 | 1.096-2.407 | **0.016** |
| Gender | 3.335 | | 1.113-9.996 | **0.032** |  | 2.625 | 1.154-5.972 | **0.021** |  | 1.802 | 0.694-4.678 | 0.226 |
| MRD at day 46 | 1.624 | | 1.190-2.271 | **0.003** |  | 1.559 | 1.219-1.994 | **0.000** |  | 1.501 | 1.128-1.997 | **0.005** |
| Age | 0.370 | | 0.098-1.404 | 0.144 |  | 0.485 | 0.161-1.465 | 0.200 |  | 0.380 | 0.122-1.188 | 0.096 |
| MRD at day 19 | 2.026 | | 1.168-3.512 | **0.012** |  | 1.503 | 1.085-2.083 | **0.014** |  | 1.577 | 1.069-2.326 | **0.022** |
| Age | 0.400 | | 0.101-1.578 | 0.191 |  | 0.600 | 0.193-1.865 | 0.378 |  | 0.423 | 0.130-1.382 | 0.154 |
| MRD at day 46 | 1.567 | | 1.112-2.209 | **0.010** |  | 1.528 | 1.178-1.983 | **0.001** |  | 1.429 | 1.051 -1.943 | **0.023** |
| Initial WBC | 1.003 | | 1.000-1.005 | **0.020** |  | 1.002 | 1.000-1.004 | **0.045** |  | 1.002 | 1.001-1.004 | **0.014** |
| MRD at day 19 | 2.322 | | 1.244-4.334 | **0.008** |  | 1.552 | 1.106-2.180 | **0.011** |  | 1.704 | 1.130-2.570 | **0.011** |
| Initial WBC | 1.003 | | 1.001-1.005 | **0.004** |  | 1.002 | 1.001-1.004 | **0.009** |  | 1.003 | 1.001-1.005 | **0.002** |
| MRD at day 46 | 1.881 | | 1.320-2.680 | **0.000** |  | 1.659 | 1.263-2.180 | **0.000** |  | 1.691 | 1.246 -2.294 | **0.001** |
| Initial blasts in BM | 1.064 | | 0.993-1.141 | 0.079 |  | 1.020 | 0.992-1.049 | 0.161 |  | 1.026 | 0.922-1.061 | 0.137 |
| MRD at day 19 | 2.291 | | 1.176-4.463 | **0.015** |  | 1.546 | 1.097-2.178 | **0.013** |  | 1.698 | 1.120-2.573 | **0.013** |
| Initial blasts in BM | 1.092 | | 1.003-1.189 | **0.044** |  | 1.031 | 1.001-1.062 | **0.040** |  | 1.038 | 1.001-1.077 | **0.044** |
| MRD at day 46 | 1.915 | | 1.363-2.691 | **0.000** |  | 1.725 | 1.319-2.255 | **0.000** |  | 1.744 | 1.296 -2.347 | **0.000** |
| Initial blasts in PB | 2.532 | | 1.203-5.329 | **0.014** |  | 1.021 | 1.003-1.039 | **0.019** |  | 1.035 | 1.009-1.062 | **0.008** |
| MRD at day 19 | 1.053 | | 1.009-1.098 | **0.016** |  | 1.517 | 1.073-2.145 | **0.018** |  | 1.577 | 1.103-2.623 | **0.016** |
| Initial blasts in PB | 1.067 | | 1.018-1.118 | **0.007** |  | 1.029 | 1.009-1.049 | **0.004** |  | 1.047 | 1.017-1.078 | **0.002** |
| MRD at day 46 | 2.124 | | 1.443-3.127 | **0.000** |  | 1.733 | 1.315-2.284 | **0.000** |  | 1.898 | 1.375 -2.621 | **0.000** |
| karyotype | 2.661 | | 0.197-35.911 | 0.461 |  | 7.867 | 1.224-50.563 | **0.030** |  | 7.185 | 0.874-59.070 | 0.067 |
| MRD at day 19 | 2.173 | | 1.130-4.178 | **0.020** |  | 1.534 | 1.068-2.205 | **0.021** |  | 1.622 | 1.049-2.509 | **0.030** |
| karyotype | 2.689 | | 0.186-38.792 | 0.468 |  | 10.480 | 1.563-70.279 | **0.016** |  | 8.409 | 1.002-70.588 | 0.050 |
| MRD at day 46 | 1.728 | | 1.173-2.547 | **0.006** |  | 1.831 | 1.362-2.461 | **0.000** |  | 1.643 | 1.163 -2.321 | **0.005** |
| Dexamethasone response | 1.351 | | 0.496-3.680 | 0.557 |  | 1.952 | 0.908-4.196 | 0.087 |  | 2.404 | 1.007-5.740 | **0.048** |
| MRD at day 19 | 1.916 | | 1.007-3.647 | **0.048** |  | 1.294 | 0.901-1.859 | 0.162 |  | 1.276 | 0.832-1.957 | 0.263 |
| Dexamethasone response | 1.407 | | 0.427-4.637 | 0.575 |  | 1.603 | 0.658-3.902 | 0.299 |  | 2.950 | 0.982 -8.864 | 0.054 |
| MRD at day 46 | 1.474 | | 0.889-2.445 | 0.133 |  | 1.347 | 0.926-1.959 | 0.120 |  | 1.064 | 0.689-1.644 | 0.779 |
| Initial Hemoglobin | 2.268 | | 1.286-3.999 | 0.343 |  | 0.992 | 0.976-1.009 | 0.373 |  | 0.994 | 0.976-1.013 | 0.545 |
| MRD at day 19 | 1.010 | | 0.989-1.032 | **0.005** |  | 1.472 | 1.049-2.065 | **0.025** |  | 1.616 | 1.083 -2.412 | **0.019** |
| Initial Hemoglobin | 1.007 | | 0.985-1.028 | 0.540 |  | 0.990 | 0.974-1.007 | 0.243 |  | 0.991 | 0.973-1.009 | 0.335 |
| MRD at day 46 | 1.710 | | 1.230-2.376 | **0.001** |  | 1.490 | 1.147-1.936 | **0.003** |  | 1.490 | 1.114 -1.993 | **0.007** |
| Initial Platelet | 0.996 | | 0.988-1.005 | 0.405 |  | 0.996 | 0.989-1.003 | 0.238 |  | 0.991 | 0.990-1.004 | 0.335 |
| MRD at day 19 | 2.134 | | 1.226-3.716 | **0.007** |  | 1.518 | 1.094-2.107 | **0.013** |  | 1.661 | 1.127 -2.449 | **0.010** |
| Initial Platelet | 0.996 | | 0.988-1.005 | 0.389 |  | 0.996 | 0.990-1.002 | 0.214 |  | 0.997 | 0.990-1.003 | 0.332 |
| MRD at day 46 | 1.654 | | 1.199-2.281 | **0.002** |  | 1.527 | 1.185-1.968 | **0.001** |  | 1.536 | 1.159 -2.034 | **0.003** |
| CNS involvement | 0.691 | | 0.333-1.433 | 0.321 |  | 0.762 | 0.418-1.389 | 0.375 |  | 0.753 | 0.390-1.455 | 0.398 |
| MRD at day 19 | 2.497 | | 1.266-4.928 | **0.008** |  | 1.642 | 1.151-2.343 | **0.006** |  | 1.781 | 1.155 -2.746 | **0.009** |
| CNS involvement | 0.613 | | 0.296-1.267 | 0.187 |  | 0.577 | 0.319-1.046 | 0.070 |  | 0.624 | 0.321-1.213 | 0.165 |
| MRD at day 46 | 1.930 | | 1.330-2.801 | **0.001** |  | 1.864 | 1.405-2.747 | **0.000** |  | 1.759 | 1.265 -2.445 | **0.001** |
| Mediastinal mass | 0.283 | | 0.061-1.301 | 0.105 |  | 0.795 | 0.326-1.940 | 0.614 |  | 0.661 | 0.234-1.866 | 0.434 |
| MRD at day 19 | 2.380 | | 1.261-4.489 | **0.007** |  | 1.559 | 1.122-2.168 | **0.008** |  | 1.689 | 1.132 -2.522 | **0.010** |
| Mediastinal mass | 0.237 | | 0.050-1.118 | 0.069 |  | 0.634 | 0.253-1.590 | 0.331 |  | 0.557 | 0.191-1.621 | 0.283 |
| MRD at day 46 | 1.827 | | 1.288-2.591 | **0.001** |  | 1.628 | 1.255-2.111 | **0.000** |  | 1.585 | 1.173 -2.142 | **0.003** |
| hepatomegaly | 0.739 | | 0.297-1.837 | 0.515 |  | 1.166 | 0.604-2.251 | 0.646 |  | 0.838 | 0.393-1.788 | 0.648 |
| MRD at day 19 | 2.178 | | 1.233-3.847 | **0.007** |  | 1.545 | 1.118-2.135 | **0.008** |  | 1.668 | 1.127 -2.468 | **0.011** |
| hepatomegaly | 0.682 | | 0.264-1.764 | 0.430 |  | 1.128 | 0.567-2.245 | 0.731 |  | 0.789 | 0.360-1.731 | 0.555 |
| MRD at day 46 | 1.681 | | 1.209-2.336 | **0.002** |  | 1.566 | 1.223-2.007 | **0.000** |  | 1.535 | 1.149 -2.050 | **0.004** |
| Splenomegaly | 0.932 | | 0.529-1.643 | 0.808 |  | 1.362 | 0.866-2.142 | 0.181 |  | 1.111 | 0.682-1.808 | 0.673 |
| MRD at day 19 | 2.159 | | 1.236-3.773 | **0.007** |  | 1.523 | 1.098-2.112 | **0.012** |  | 1.649 | 1.115 -2.439 | **0.012** |
| Splenomegaly | 0.950 | | 0.534-1.689 | 0.860 |  | 1.434 | 0.912-2.255 | 0.119 |  | 1.175 | 0.723-1.911 | 0.514 |
| MRD at day 46 | 1.658 | | 1.197-2.296 | **0.002** |  | 1.563 | 1.223-1.999 | **0.000** |  | 1.511 | 1.135 -2.010 | **0.005** |
| Immunophenotype | 1.316 | | 0.854-2.029 | 0.214 |  | 1.046 | 0.746-1.467 | 0.795 |  | 0.992 | 0.680-1.448 | 0.968 |
| MRD at day 19 | 2.133 | | 1.251-3.638 | **0.005** |  | 1.553 | 1.124-2.146 | **0.008** |  | 1.659 | 1.123 -2.449 | **0.011** |
| Immunophenotype | 1.176 | | 0.757-1.827 | 0.470 |  | 0.924 | 0.657-1.300 | 0.651 |  | 0.871 | 0.593-1.279 | 0.481 |
| MRD at day 46 | 1.627 | | 1.173-2.256 | **0.004** |  | 1.588 | 1.236-2.040 | **0.000** |  | 1.547 | 1.157 -2.070 | **0.003** |
| SIL-TAL1 translocation | 1.355 | | 0.275-6.672 | 0.708 |  | 1.054 | 0.343-3.235 | 0.927 |  | 1.114 | 0.308-4.032 | 0.870 |
| MRD at day 19 | 2.225 | | 1.226-4.037 | **0.009** |  | 1.556 | 1.110-2.180 | **0.010** |  | 1.674 | 1.116 -2.510 | **0.013** |
| SIL-TAL1 translocation | 1.019 | | 0.209-4.964 | 0.981 |  | 1.052 | 0.339-3.272 | 0.930 |  | 1.006 | 0.277-3.657 | 0.992 |
| MRD at day 46 | 1.655 | | 1.179-2.323 | **0.004** |  | 1.578 | 1.218-2.045 | **0.001** |  | 1.517 | 1.127 -2.042 | **0.006** |
| Myc positive | 7.220 | | 0.883-62.603 | 0.073 |  | 2.062 | 0.267-15.902 | 0.488 |  | 2.967 | 0.373-23.586 | 0.304 |
| MRD at day 19 | 2.296 | | 1.302-4.049 | **0.004** |  | 1.572 | 1.132-2.183 | **0.007** |  | 1.705 | 1.149 -2.532 | **0.008** |
| Myc positive | 4.529 | | 0.536-38.260 | 0.165 |  | 1.886 | 0.245-14.517 | 0.542 |  | 2.316 | 0.295-18.164 | 0.424 |
| MRD at day 46 | 1.710 | | 1.226-2.386 | **0.002** |  | 1.589 | 1.237-2.042 | **0.000** |  | 1.540 | 1.152 -2.058 | **0.004** |
| MLL rearrangement | 0.000 | | 0.000- | 0.988 |  | 0.000 | 0.000- | 0.984 |  | 0.000 | 0.000- | 0.986 |
| MRD at day 19 | 2.163 | | 1.253-3.734 | **0.006** |  | 1.574 | 1.141-2.170 | **0.006** |  | 1.684 | 1.147 -2.472 | **0.008** |
| MLL rearrangement | 0.000 | | 0.000- | 0.985 |  | 0.000 | 0.000- | 0.979 |  | 0.000 | 0.000- | 0.335 |
| MRD at day 46 | 1.637 | | 1.187-2.257 | **0.003** |  | 1.560 | 1.221-1.994 | **0.000** |  | 1.504 | 1.133 -1.996 | **0.005** |
| WT1 positive | 0.410 | | 0.128-1.314 | 0.134 |  | 0.424 | 0.145-1.242 | 0.118 |  | 0.362 | 0.129-1.019 | 0.054 |
| MRD at day 19 | 2.449 | | 1.369-4.379 | **0.003** |  | 1.944 | 1.220-3.098 | **0.005** |  | 1.906 | 1.266 -2.870 | **0.002** |
| WT1 positive | 0.705 | | 0.223-2.227 | 0.551 |  | 0.366 | 0.139-0.965 | 0.042 |  | 0.541 | 0.195-1.497 | 0.237 |
| MRD at day 46 | 1.689 | | 1.213-2.353 | **0.002** |  | 1.694 | 1.313-2.184 | **0.000** |  | 1.584 | 1.182 -2.122 | **0.002** |
| CDKN2A/CEP9 | 0.947 | | 0.258-3.476 | 0.934 |  | 0.983 | 0.382-2.531 | 0.972 |  | 1.057 | 0.373-2.995 | 0.917 |
| MRD at day 19 | 2.146 | | 1.224-3.763 | **0.008** |  | 1.548 | 1.115-2.149 | **0.009** |  | 1.664 | 1.123 -2.465 | **0.011** |
| CDKN2A/CEP9 | 0.840 | | 0.228-3.091 | 0.793 |  | 0.905 | 0.354-2.313 | 0.836 |  | 0.949 | 0.336-2.675 | 0.920 |
| MRD at day 46 | 1.645 | | 1.187-2.279 | **0.003** |  | 1.569 | 1.224-2.012 | **0.000** |  | 1.514 | 1.136-2.018 | **0.005** |

T-ALL, T-cell acute lymphoblastic leukemia; WBC, white blood cells; BM, bone marrow; PB, peripheral blood; MRD, minimal residual disease. Cox regression analysis was used for multivariate analysis; Bold values indicate statistical significance at p<0.05.
