# Supplementary material for: A Novel Risk Defining System for Pediatric T-Cell Acute Lymphoblastic Leukemia From CCCG-ALL-2015 Group
Source: Front Oncol. 2022 Feb 28;12:841179. doi: 10.3389/fonc.2022.841179 (PMC8920043; doi:10.3389/fonc.2022.841179)
Supplement: Supplementary file 8 [file Table_8.docx]

**Supplementary Table 8. Evaluation of the new risk score for survival of children with T-ALL using Cox hazards model.**

| Variables | Overall survival (OS) | | | |  | Event-free survival (EFS) | | |  | Relapse-free survival (RFS) | | |
| --- | --- | --- | --- | --- | --- | --- | --- | --- | --- | --- | --- | --- |
|  | OR | 95%CI (OR) | | *p*-value |  | OR | 95%CI (OR) | *p*-value |  | OR | 95%CI (OR) | p-value |
| New risk score^*^ | 3.548 | | 1.788-7.041 | **0.000** |  | 2.745 | 1.651-4.565 | **0.000** |  | 3.385 | 1.842-6.224 | **0.000** |
| New risk score^#^ | 4.408 | | 1.313-14.803 | **0.016** |  | 2.365 | 1.233-4.534 | **0.010** |  | 2.676 | 1.222-5.860 | **0.014** |
| Gender^#^ | 4.331 | | 0.792-23.678 | 0.091 |  | 2.484 | 0.795-7.758 | 0.117 |  | 1.403 | 0.354-5.567 | 0.630 |
| Age^#^ | 1.045 | | 0.818-1.334 | 0.725 |  | 0.941 | 0.822-1.077 | 0.378 |  | 0.938 | 0.803-1.095 | 0.415 |
| Initial WBC^#^ | 1.002 | | 0.997-1.007 | 0.422 |  | 1.002 | 0.998-1.005 | 0.322 |  | 1.000 | 0.996-1.004 | 0.841 |
| Initial blasts in BM^#^ | 1.039 | | 0.910-1.186 | 0.574 |  | 1.030 | 0.966-1.098 | 0.368 |  | 1.055 | 0.963-1.157 | 0.251 |
| Initial blasts in PB^#^ | 1.019 | | 0.952-1.091 | 0.590 |  | 0.992 | 0.964-1.022 | 0.610 |  | 1.012 | 0.973-1.052 | 0.558 |
| karyotype^#^ | 1.114 | | 0.410-3.028 | 0.833 |  | 1.211 | 0.646-2.267 | 0.550 |  | 1.059 | 0.538-2.084 | 0.860 |
| New risk score^&^ | 17.821 | | 1.673-189.845 | **0.017** |  | 6.372 | 1.672-24.287 | **0.007** |  | 7.008 | 1.422-34.535 | **0.017** |
| Dexamethasone response response^&^ | 0.195 | | 0.010-3.918 | 0.286 |  | 0.546 | 0.100-2.984 | 0.485 |  | 1.828 | 0.170-19.599 | 0.618 |
| MRD at day 19^&^ | 0.918 | | 0.135-6.218 | 0.930 |  | 0.551 | 0.249-1.219 | 0.141 |  | 0.477 | 0.183-1.241 | 0.129 |
| MRD at day 46^&^ | 0.322 | | 0.096-1.079 | 0.066 |  | 0.716 | 0.332-1.546 | 0.395 |  | 0.692 | 0.288-1.664 | 0.411 |

T-ALL, T-cell acute lymphoblastic leukemia; WBC, white blood cells; BM, bone marrow; PB, peripheral blood; MRD, minimal residual disease. Cox hazards model was used for univariate and multivariate analyses; ^*^, univariate analysis; ^#^, and ^&^, multivariate analysis; Bold values indicate statistical significance at p<0.05.
