# Supplementary material for: A Novel Risk Defining System for Pediatric T-Cell Acute Lymphoblastic Leukemia From CCCG-ALL-2015 Group
Source: Front Oncol. 2022 Feb 28;12:841179. doi: 10.3389/fonc.2022.841179 (PMC8920043; doi:10.3389/fonc.2022.841179)
Supplement: Supplementary file 9 [file Table_9.docx]

Supplementary Table 9. The targeted gene sequencing of 55 children with T-ALL and the correlations with MRD.

| Targeted gene sequencing | N (%) | MRD-D19 (N, %) | | | | | *P^*^* value | *R* | MRD-D46 (N, %) | | | | | *P^#^* value | *R* |
| --- | --- | --- | --- | --- | --- | --- | --- | --- | --- | --- | --- | --- | --- | --- | --- |
|  |  | MRD-1 level | MRD-2 level | MRD-3 level | MRD-4 level | Unkno-wn |  |  | MRD-1 level | MRD-2 level | MRD-3 level | MRD-4 level | Unkno-wn |  |  |
| **Total** | 55 (100.0) | 29 (52.7) | 1 (1.8) | 8 (14.5) | 15 (27.3) | 2 (3.6) |  |  | 44 (80.0) | 3 (5.5) | 4 (7.3) | 1 (1.8) | 3 (5.5) |  |  |
| **NOTCH1** |  |  |  |  |  |  | 0.829 | - |  |  |  |  |  | 0.358 | - |
| Normal | 18 (32.7) | 9 (50.0) | 1 (5.6) | 2 (11.1) | 5 (27.8) | 1 (5.6) |  |  | 16 (88.9) | 1 (5.6) | 0 (0.0) | 0 (0.0) | 1 (5.6) |  |  |
| Abnormal | 37 (67.3) | 20 (54.1) | 0 (0.0) | 6 (16.2) | 10 (27.0) | 1 (2.7) |  |  | 28 (75.7) | 2 (5.4) | 4 (10.8) | 1 (2.7) | 2 (5.4) |  |  |
| **NOTCH2** |  |  |  |  |  |  | 0.629 | - |  |  |  |  |  | 0.822 | - |
| Normal | 52 (94.5) | 28 (53.8) | 1 (1.9) | 7 (13.5) | 14 (26.9) | 2 (3.8) |  |  | 42 (80.8) | 2 (3.8) | 4 (7.7) | 1 (1.9) | 3 (5.8) |  |  |
| Abnormal | 3 (5.5) | 1 (33.3) | 0 (0.0) | 1 (33.3) | 1 (33.3) | 0 (0.0) |  |  | 2 (66.7) | 1 (33.3) | 0 (0.0) | 0 (0.0) | 0 (0.0) |  |  |
| **FBXW7** |  |  |  |  |  |  | 0.818 | - |  |  |  |  |  | 0.793 | - |
| Normal | 36 (65.5) | 18 (50.0) | 1 (2.8) | 6 (16.7) | 10 (27.8) | 1 (2.8) |  |  | 29 (80.6) | 3 (8.3) | 1 (2.8) | 1 (2.8) | 2 (5.6) |  |  |
| Abnormal | 19 (34.5) | 11 (57.9) | 0 (0.0) | 2 (10.5) | 5 (26.3) | 1 (5.3) |  |  | 15 (78.9) | 3 (15.8) | 0 (0.0) | 0 (0.0) | 1 (5.3) |  |  |
| **NOTCH1 and FBXW7** |  |  |  |  |  |  | 0.742 | - |  |  |  |  |  | 0.992 | - |
| Normal | 38 (69.1) | 19 (50.0) | 1 (2.6) | 6 (15.8) | 11 (28.9) | 1 (2.6) |  |  | 30 (78.9) | 3 (7.9) | 2 (5.3) | 1 (2.6) | 2 (5.3) |  |  |
| Abnormal | 17 (30.9) | 10 (58.8) | 0 (0.0) | 2 (11.8) | 4 (23.5) | 1 (5.9) |  |  | 14 (82.4) | 0 (0.0) | 2 (11.8) | 0 (0.0) | 1 (5.9) |  |  |
| **KMT2D** |  |  |  |  |  |  | 0.243 | - |  |  |  |  |  | 0.807 | - |
| Normal | 44 (80.0) | 22 (50.0) | 0 (0.0) | 6 (13.6) | 15 (34.1) | 1 (2.3) |  |  | 35 (79.5) | 3 (6.8) | 3 (6.8) | 1 (2.3) | 2 (4.5) |  |  |
| Abnormal | 11 (20.0) | 7 (63.6) | 1 (9.1) | 2 (18.2) | 0 (0.0) | 1 (9.1) |  |  | 9 (81.8) | 0 (0.0) | 1 (9.1) | 0 (0.0) | 1 (9.1) |  |  |
| **WT1** |  |  |  |  |  |  | 0.817 | - |  |  |  |  |  | 0.325 | - |
| Normal | 44 (80.0) | 24 (54.5) | 0 (0.0) | 7 (15.9) | 11 (25.0) | 2 (4.5) |  |  | 34 (77.3) | 3 (6.8) | 3 (6.8) | 1 (2.3) | 3 (6.8) |  |  |
| Abnormal | 11 (20.0) | 5 (45.5) | 1 (9.1) | 1 (9.1) | 4 (36.4) | 0 (0.0) |  |  | 10 (90.9) | 0 (0.0) | 1 (9.1) | 0 (0.0) | 0 (0.0) |  |  |
| **FAT1** |  |  |  |  |  |  | 0.861 | - |  |  |  |  |  | 0.470 | - |
| Normal | 45 (81.8) | 24 (53.3) | 0 (0.0) | 7 (15.6) | 12 (26.7) | 2 (4.4) |  |  | 37 (82.2.5) | 3 (6.7) | 2 (4.4) | 0 (0.0) | 3 (6.7) |  |  |
| Abnormal | 10 (18.2) | 5 (50.0) | 1 (10.0) | 1 (10.0) | 3 (30.0) | 0 (0.0) |  |  | 7 (70.0) | 0 (0.0) | 2 (20.0) | 1 (10.0) | 0 (0.0) |  |  |
| **CREBBP** |  |  |  |  |  |  | 0.051 | - |  |  |  |  |  | 0.190 | - |
| Normal | 46 (83.6) | 27 (58.7) | 0 (0.0) | 6 (13.0) | 13 (28.3) | 0 (0.0) |  |  | 38 (82.6) | 3 (6.5) | 3 (6.5) | 1 (2.2) | 1 (2.2) |  |  |
| Abnormal | 9 (16.4) | 2 (22.2) | 1 (11.1) | 2 (22.2) | 2 (22.2) | 2 (22.2) |  |  | 6 (66.7) | 0 (0.0) | 1 (11.1) | 0 (0.0) | 2 (22.2) |  |  |
| **RELN** |  |  |  |  |  |  | 0.152 | - |  |  |  |  |  | 0.082 | - |
| Normal | 48 (87.3) | 27 (56.3) | 1 (2.1) | 6 (12.5) | 13 (27.1) | 1 (2.1) |  |  | 40 (83.3) | 2 (4.2) | 4 (8.3) | 1 (2.1) | 1 (2.1) |  |  |
| Abnormal | 7 (12.7) | 2 (28.6) | 0 (0.0) | 2 (28.6) | 2 (28.6) | 1 (14.3) |  |  | 4 (57.1) | 1 (14.3) | 0 (0.0) | 0 (0.0) | 2 (28.6) |  |  |
| **PHF6** |  |  |  |  |  |  | 0.800 | - |  |  |  |  |  | 0.221 | - |
| Normal | 48 (87.3) | 25 (52.1) | 1 (2.1) | 7 (14.6) | 13 (27.1) | 2 (4.2) |  |  | 37 (77.1) | 3 (6.3) | 4 (8.3) | 1 (2.1) | 3 (6.3) |  |  |
| Abnormal | 7 (12.7) | 4 (57.1) | 0 (0.0) | 1 (14.3) | 2 (28.6) | 0 (0.0) |  |  | 7 (100.0) | 0 (0.0) | 0 (0.0) | 0 (0.0) | 0 (0.0) |  |  |
| **PTEN** |  |  |  |  |  |  | 0.315 | - |  |  |  |  |  | 0.263 | - |
| Normal | 49 (89.1) | 27 (55.1) | 1 (2.0) | 7 (14.3) | 12 (24.5) | 2 (4.1) |  |  | 38 (77.6) | 3 (6.1) | 4 (8.2) | 1 (2.0) | 3 (6.1) |  |  |
| Abnormal | 6 (10.9) | 2 (33.3) | 0 (0.0) | 1 (16.7) | 3 (50.0) | 0 (0.0) |  |  | 6 (100.0) | 0 (0.0) | 0 (0.0) | 0 (0.0) | 0 (0.0) |  |  |
| **JAK3** |  |  |  |  |  |  | 0.374 | - |  |  |  |  |  | 0.057 | - |
| Normal | 49 (89.1) | 27 (55.1) | 1 (2.0) | 8 (16.3) | 12 (24.5) | 1 (2.0) |  |  | 41 (83.7) | 2 (4.1) | 3 (6.1) | 1 (2.0) | 2 (4.1) |  |  |
| Abnormal | 6 (10.9) | 2 (33.3) | 0 (0.0) | 0 (0.0) | 3 (50.0) | 1 (16.7) |  |  | 3 (50.0) | 1 (16.7) | 1 (16.7) | 0 (0.0) | 1 (16.7) |  |  |
| **DNM2** |  |  |  |  |  |  | 0.106 | - |  |  |  |  |  | 0.394 | - |
| Normal | 49 (89.1) | 27 (55.1) | 1 (2.0) | 8 (16.3) | 11 (22.4) | 2 (4.1) |  |  | 40 (81.6) | 3 (6.1) | 3 (6.1) | 0 (0.0) | 3 (6.1) |  |  |
| Abnormal | 6 (10.9) | 2 (33.3) | 0 (0.0) | 0 (0.0) | 4 (66.7) | 0 (0.0) |  |  | 4 (66.7) | 1 (16.7) | 0 (0.0) | 1 (16.7) | 0 (0.0) |  |  |
| **KRAS** |  |  |  |  |  |  | 0.444 | - |  |  |  |  |  | 0.312 | - |
| Normal | 50 (90.9) | 26 (52.0) | 1 (2.0) | 6 (12.0) | 15 (30.0) | 2 (4.0) |  |  | 39 (78.0) | 3 (6.0) | 4 (8.0) | 1 (2.0) | 3 (6.0) |  |  |
| Abnormal | 5 (9.1) | 3 (60.0) | 0 (0.0) | 2 (40.0) | 0 (0.0) | 0 (0.0) |  |  | 5 (100.0) | 0 (0.0) | 0 (0.0) | 0 (0.0) | 0 (0.0) |  |  |
| **ARID1A** |  |  |  |  |  |  | 0.454 | - |  |  |  |  |  | 0.371 | - |
| Normal | 51 (92.7) | 26 (51.0) | 1 (2.0) | 8 (15.7) | 14 (27.5) | 2 (3.9) |  |  | 40 (78.4) | 3 (5.9) | 4 (7.8) | 1 (2.0) | 3 (5.9) |  |  |
| Abnormal | 4 (7.3) | 3 (75.0) | 0 (0.0) | 0 (0.0) | 1 (25.0) | 0 (0.0) |  |  | 4 (100.0) | 0 (0.0) | 0 (0.0) | 0 (0.0) | 0 (0.0) |  |  |
| **JAK2** |  |  |  |  |  |  | 0.697 | - |  |  |  |  |  | 0.090 | - |
| Normal | 51 (92.7) | 26 (51.0) | 1 (2.0) | 8 (15.7) | 15 (29.4) | 1 (2.0) |  |  | 42 (82.4) | 3 (5.9) | 3 (5.9) | 1 (2.0) | 2 (3.9) |  |  |
| Abnormal | 4 (7.3) | 3 (75.0) | 0 (0.0) | 0 (0.0) | 0 (0.0) | 1 (25.0) |  |  | 2 (50.0) | 0 (0.0) | 1 (25.0) | 0 (0.0) | 1 (25.0) |  |  |
| **TP53** |  |  |  |  |  |  | 0.159 | - |  |  |  |  |  | 0.674 | - |
| Normal | 51 (92.7) | 28 (54.9) | 1 (2.0) | 8 (15.7) | 12 (23.5) | 2 (3.9) |  |  | 41 (80.4) | 2 (3.9) | 4 (7.8) | 1 (2.0) | 3 (5.9) |  |  |
| Abnormal | 4 (7.3) | 1 (25.0) | 0 (0.0) | 0 (0.0) | 3 (75.0) | 0 (0.0) |  |  | 3 (75.0) | 1 (25.0) | 0 (0.0) | 0 (0.0) | 0 (0.0) |  |  |
| **EP300** |  |  |  |  |  |  | **0.038** | **0.281** |  |  |  |  |  | **0.002** | **0.415** |
| Normal | 51 (92.7) | 28 (56.0) | 1 (2.0) | 7 (14.0) | 14 (28.0) | 0 (0.0) |  |  | 42 (84.0) | 3 (6.0) | 3 (6.0) | 1 (2.0) | 1 (2.0) |  |  |
| Abnormal | 4 (7.3) | 1 (20.0) | 0 (0.0) | 1 (20.0) | 1 (20.0) | 2 (40.0) |  |  | 2 (40.0) | 0 (0.0) | 1 (20.0) | 0 (0.0) | 2 (40.0) |  |  |
| **EZH2** |  |  |  |  |  |  | 0.159 | - |  |  |  |  |  | 0.959 | - |
| Normal | 51 (92.7) | 28 (54.9) | 1 (2.0) | 8 (15.7) | 12 (23.5) | 2 (3.9) |  |  | 41 (80.4) | 3 (5.9) | 3 (5.9) | 1 (2.0) | 3 (5.9) |  |  |
| Abnormal | 4 (7.3) | 1 (25.0) | 0 (0.0) | 0 (0.0) | 3 (75.0) | 0 (0.0) |  |  | 3 (75.0) | 0 (0.0) | 1 (25.0) | 0 (0.0) | 0 (0.0) |  |  |
| **PRDM1** |  |  |  |  |  |  | **0.027** | **0.299** |  |  |  |  |  | 0.601 | - |
| Normal | 51 (92.7) | 29 (56.9) | 1 (2.0) | 7 (13.7) | 12 (23.5) | 2 (3.9) |  |  | 42 (82.4) | 2 (3.9) | 3 (5.9) | 1 (2.0) | 3 (5.9) |  |  |
| Abnormal | 4 (7.3) | 0 (0.0) | 0 (0.0) | 1 (25.0) | 3 (75.0) | 0 (0.0) |  |  | 2 (50.0) | 1 (25.0) | 1 (25.0) | 0 (0.0) | 0 (0.0) |  |  |
| **JAK1** |  |  |  |  |  |  | **0.014** | **0.314** |  |  |  |  |  | **0.004** | **0.379** |
| Normal | 51 (92.7) | 29 (56.9) | 1 (2.0) | 7 (13.7) | 13 (25.5) | 1 (2.0) |  |  | 43 (84.3) | 2 (3.9) | 3 (5.9) | 1 (2.0) | 2 (3.9) |  |  |
| Abnormal | 4 (7.3) | 0 (0.0) | 0 (0.0) | 1 (25.0) | 2 (50.0) | 1 (25.0) |  |  | 1 (25.0) | 1 (25.0) | 1 (25.0) | 0 (0.0) | 1 (25.0) |  |  |
| **USP7** |  |  |  |  |  |  | 0.494 | **-** |  |  |  |  |  | 0.601 | **-** |
| Normal | 51 (92.7) | 28 (54.9) | 1 (2.0) | 6 (11.8) | 14 (27.5) | 2 (3.9) |  |  | 41 (80.4) | 3 (5.9) | 4 (7.8) | 0 (0.0) | 3 (5.9) |  |  |
| Abnormal | 4 (7.3) | 1 (25.0) | 0 (0.0) | 2 (50.0) | 1 (25.0) | 0 (0.0) |  |  | 3 (75.0) | 0 (0.0) | 0 (0.0) | 1 (0.0) | 0 (0.0) |  |  |
| **DNMT3A** |  |  |  |  |  |  | 0.629 | - |  |  |  |  |  | **0.040** | **0.278** |
| Normal | 52 (94.5) | 28 (53.8) | 1 (1.9) | 7 (13.5) | 14 (26.9) | 2 (3.8) |  |  | 43 (82.7) | 2 (3.8) | 4 (7.7) | 1 (1.9) | 2 (3.8) |  |  |
| Abnormal | 3 (5.5) | 1 (33.3) | 0 (0.0) | 1 (33.3) | 1 (33.3) | 0 (0.0) |  |  | 1 (33.3) | 1 (33.3) | 0 (0.0) | 0 (0.0) | 1 (33.3) |  |  |
| **NRAS** |  |  |  |  |  |  | 0.629 | - |  |  |  |  |  | 0.443 | - |
| Normal | 52 (94.5) | 28 (53.8) | 1 (1.9) | 7 (13.5) | 14 (26.9) | 2 (3.8) |  |  | 41 (78.8) | 3 (5.8) | 4 (7.7) | 1 (1.9) | 3 (5.8) |  |  |
| Abnormal | 3 (5.5) | 1 (33.3) | 0 (0.0) | 1 (33.3) | 1 (33.3) | 0 (0.0) |  |  | 3 (100.0) | 0 (0.0) | 0 (0.0) | 0 (0.0) | 0 (0.0) |  |  |
| **CUX1** |  |  |  |  |  |  | 0.738 | - |  |  |  |  |  | 0.443 | - |
| Normal | 52 (94.5) | 27 (51.9) | 1 (1.9) | 8 (15.4) | 14 (26.9) | 2 (3.8) |  |  | 41 (78.8) | 3 (5.8) | 4 (7.7) | 1 (1.9) | 3 (5.8) |  |  |
| Abnormal | 3 (5.5) | 2 (66.7) | 0 (0.0) | 0 (0.0) | 1 (33.3) | 0 (0.0) |  |  | 3 (100.0) | 0 (0.0) | 0 (0.0) | 0 (0.0) | 0 (0.0) |  |  |
| **WHSC1** |  |  |  |  |  |  | 0.738 | - |  |  |  |  |  | 0.443 | - |
| Normal | 52 (94.5) | 27 (51.9) | 1 (1.9) | 8 (15.4) | 14 (26.9) | 2 (3.8) |  |  | 41 (78.8) | 3 (5.8) | 4 (7.7) | 1 (1.9) | 3 (5.8) |  |  |
| Abnormal | 3 (5.5) | 2 (66.7) | 0 (0.0) | 0 (0.0) | 1 (33.3) | 0 (0.0) |  |  | 3 (100.0) | 0 (0.0) | 0 (0.0) | 0 (0.0) | 0 (0.0) |  |  |
| **ASXL2** |  |  |  |  |  |  | 0.296 | - |  |  |  |  |  | 0.959 | - |
| Normal | 52 (94.5) | 28 (54.9) | 1 (2.0) | 7 (13.7) | 13 (25.5) | 2 (3.9) |  |  | 41 (80.4) | 3 (5.9) | 3 (5.9) | 1 (2.0) | 3 (5.9) |  |  |
| Abnormal | 3 (5.5) | 1 (25.0) | 0 (0.0) | 1 (25.0) | 2 (50.0) | 0 (0.0) |  |  | 3 (75.0) | 0 (0.0) | 1 (0.0) | 1 (25.0) | 0 (0.0) |  |  |
| **IL-7R** |  |  |  |  |  |  | 0.738 | - |  |  |  |  |  | 0.443 | - |
| Normal | 52 (94.5) | 27 (51.9) | 1 (1.9) | 8 (15.4) | 14 (26.9) | 2 (3.8) |  |  | 41 (78.8) | 3 (5.8) | 4 (7.7) | 1 (1.9) | 3 (5.8) |  |  |
| Abnormal | 3 (5.5) | 2 (66.7) | 0 (0.0) | 0 (0.0) | 1 (33.3) | 0 (0.0) |  |  | 3 (100.0) | 0 (0.0) | 0 (0.0) | 0 (0.0) | 0 (0.0) |  |  |
| **TET2** |  |  |  |  |  |  | 0.629 | - |  |  |  |  |  | 0.443 | - |
| Normal | 52 (94.5) | 28 (53.8) | 1 (1.9) | 7 (13.5) | 14 (26.9) | 2 (3.8) |  |  | 41 (78.8) | 3 (5.8) | 4 (7.7) | 1 (1.9) | 3 (5.8) |  |  |
| Abnormal | 3 (5.5) | 1 (33.3) | 0 (0.0) | 1 (33.3) | 1 (33.3) | 0 (0.0) |  |  | 3 (100.0) | 0 (0.0) | 0 (0.0) | 0 (0.0) | 0 (0.0) |  |  |
| **BCORL1** |  |  |  |  |  |  | 0.629 | - |  |  |  |  |  | 0.392 | - |
| Normal | 52 (94.5) | 28 (53.8) | 1 (1.9) | 7 (13.5) | 14 (26.9) | 2 (3.8) |  |  | 42 (80.8) | 3 (5.8) | 4 (7.7) | 0 (0.0) | 3 (5.8) |  |  |
| Abnormal | 3 (5.5) | 1 (33.3) | 0 (0.0) | 1 (33.3) | 1 (33.3) | 0 (0.0) |  |  | 2 (66.7) | 0 (0.0) | 0 (0.0) | 1 (33.3) | 0 (0.0) |  |  |

T-ALL, T-cell acute lymphoblastic leukemia; MRD, minimal residual disease; ^*^, significance of correlation analysis between gene mutation and MRD-D19 level 1-4; ^#^, significance of correlation analysis between gene mutation and MRD-D46 level 1-4; Spearman's correlation was used for correlation analysis; R, Spearman's correlation coefficient; Bold values indicate statistical significance at p<0.05.
