# Supplementary material for: A Novel Risk Defining System for Pediatric T-Cell Acute Lymphoblastic Leukemia From CCCG-ALL-2015 Group
Source: Front Oncol. 2022 Feb 28;12:841179. doi: 10.3389/fonc.2022.841179 (PMC8920043; doi:10.3389/fonc.2022.841179)
Supplement: Supplementary file 10 [file Table_10.docx]

Supplementary Table 10. The targeted gene sequencing of 55 children with T-ALL and the comparisons of their survival.

| Targeted gene sequencing | N (%) | 2-year OS (SE) | *P^*^* value | 2-year EFS (SE) | *P^#^* value | 2-year DFS (SE) | *P^&^* value |
| --- | --- | --- | --- | --- | --- | --- | --- |
| **Total** | 55 (100.0) | 83.4 (5.4) |  | 66.9 (7.7) |  | 64.0 (7.9) |  |
| **NOTCH1** |  |  | 0.824 |  | 0.442 |  | 0.431 |
| Normal | 18 (32.7) | 82.4 (9.2) |  | 71.3 (10.9) |  | 71.3 (10.9) |  |
| Abnormal | 37 (67.3) | 84.1 (6.7) |  | 63.5 (11.0) |  | 57.7 (11.7) |  |
| **NOTCH2** |  |  | 0.522 |  | 0.577 |  | 0.582 |
| Normal | 52 (94.5) | 82.6 (5.6) |  | 67.8 (7.7) |  | 64.8 (8.0) |  |
| Abnormal | 3 (5.5) | 100.0 (0.0) |  | 50.0 (35.4) |  | 50.0 (35.4) |  |
| **FBXW7** |  |  | 0.108 |  | 0.625 |  | 0.682 |
| Normal | 36 (65.5) | 87.7 (5.8) |  | 70.0 (8.0) |  | 67.0 (8.3) |  |
| Abnormal | 19 (34.5) | 72.3 (12.3) |  | 50.3 (21.8) |  | 46.5 (21.0) |  |
| **NOTCH1 and FBXW7** |  |  | 0.280 |  | 0.846 |  | 0.887 |
| Normal | 38 (69.1) | 85.8 (5.9) |  | 69.0 (7.9) |  | 66.2 (8.0) |  |
| Abnormal | 17 (30.9) | 74.1 (13.4) |  | 39.0 (28.2) |  | 35.3 (26.1) |  |
| **KMT2D** |  |  | 0.471 |  | 0.984 |  | 0.903 |
| Normal | 44 (80.0) | 81.0 (6.5) |  | 63.6 (9.0) |  | 62.5 (9.2) |  |
| Abnormal | 11 (20.0) | 90.9 (8.7) |  | 79.5 (13.1) |  | 70.1 (14.7) |  |
| **WT1** |  |  | 0.536 |  | 0.716 |  | 0.763 |
| Normal | 44 (80.0) | 84.4 (6.0) |  | 69.1 (7.7) |  | 65.1 (8.2) |  |
| Abnormal | 11 (20.0) | 80.0 (12.2) |  | 69.3 (15.0) |  | 69.3 (15.0) |  |
| **FAT1** |  |  | 0.160 |  | 0.409 |  | 0.406 |
| Normal | 45 (81.8) | 80.0 (6.4) |  | 63.1 (8.5) |  | 59.3 (8.8) |  |
| Abnormal | 10 (18.2) | 100.0 (0.0) |  | 85.7 (13.2) |  | 85.7 (13.2) |  |
| **CREBBP** |  |  | **0.023** |  | **0.005** |  | **0.002** |
| Normal | 46 (83.6) | 90.2 (4.7) |  | 71.0 (8.8) |  | 70.9 (8.8) |  |
| Abnormal | 9 (16.4) | 55.6 (16.6) |  | 44.4 (16.6) |  | 33.3 (15.7) |  |
| **RELN** |  |  | 0.052 |  | **0.002** |  | **0.012** |
| Normal | 48 (87.3) | 84.7 (5.8) |  | 79.6 (6.5) |  | 73.5 (7.0) |  |
| Abnormal | 7 (12.7) | 71.4 (17.1) |  | 31.7 (16.8) |  | 32.8 (17.0) |  |
| **PHF6** |  |  | 0.813 |  | 0.957 |  | 0.881 |
| Normal | 48 (87.3) | 80.9 (6.2) |  | 69.6 (7.2) |  | 66.5 (7.5) |  |
| Abnormal | 7 (12.7) | 100.0 (0.0) |  | 42.9 (31.0) |  | 42.9 (31.0) |  |
| **PTEN** |  |  | 0.889 |  | 0.187 |  | 0.227 |
| Normal | 49 (89.1) | 83.6 (5.7) |  | 69.3 (8.1) |  | 66.0 (8.5) |  |
| Abnormal | 6 (10.9) | 80.0 (17.9) |  | 40.0 (21.9) |  | 40.0 (21.9) |  |
| **JAK3** |  |  | 0.600 |  | 0.462 |  | 0.487 |
| Normal | 49 (89.1) | 83.8 (5.6) |  | 67.2 (8.1) |  | 64.1 (8.4) |  |
| Abnormal | 6 (10.9) | 88.3 (15.2) |  | 66.7 (19.2) |  | 66.7 (19.2) |  |
| **DNM2** |  |  | 0.335 |  | 0.534 |  | 0.556 |
| Normal | 49 (89.1) | 81.6 (5.9) |  | 68.6 (7.8) |  | 65.4 (8.2) |  |
| Abnormal | 6 (10.9) | 100.0 (0.0) |  | 53.3 (28.0) |  | 53.3 (24.8) |  |
| **KRAS** |  |  | 0.313 |  | 0.167 |  | 0.147 |
| Normal | 50 (90.9) | 81.4 (6.0) |  | 63.6 (8.2) |  | 60.3 (8.4) |  |
| Abnormal | 5 (9.1) | 100.0 (0.0) |  | 100.0 (0.0) |  | 100.0 (0.0) |  |
| **ARID1A** |  |  | 0.422 |  | 0.270 |  | 0.556 |
| Normal | 51 (92.7) | 82.1 (5.8) |  | 65.0 (7.9) |  | 61.9 (8.1) |  |
| Abnormal | 4 (7.3) | 100.0 (0.0) |  | 100.0 (0.0) |  | 100.0 (0.0) |  |
| **JAK2** |  |  | 0.608 |  | 0.898 |  | 0.826 |
| Normal | 51 (92.7) | 84.1 (5.6) |  | 66.5 (7.9) |  | 63.2 (8.2) |  |
| Abnormal | 4 (7.3) | 75.0 (21.2) |  | 75.0 (21.2) |  | 75.0 (21.2) |  |
| **TP53** |  |  | 0.414 |  | **0.001** |  | **0.001** |
| Normal | 51 (92.7) | 84.8 (5.3) |  | 70.6 (7.8) |  | 67.5 (8.1) |  |
| Abnormal | 4 (7.3) | 50.0 (35.4) |  | 25.0 (21.7) |  | 25.0 (21.7) |  |
| **EP300** |  |  | **0.000** |  | **0.001** |  | **0.005** |
| Normal | 51 (92.7) | 88.8 (4.7) |  | 70.4 (7.8) |  | 68.0 (8.0) |  |
| Abnormal | 4 (7.3) | 25.0 (21.7) |  | 25.0 (21.7) |  | 25.0 (21.7) |  |
| **EZH2** |  |  | 0.550 |  | 0.961 |  | 0.947 |
| Normal | 51 (92.7) | 84.5 (5.4) |  | 66.9 (8.0) |  | 63.8 (8.3) |  |
| Abnormal | 4 (7.3) | 66.7 (27.2) |  | 66.7 (27.2) |  | 66.7 (27.2) |  |
| **PRDM1** |  |  | 0.059 |  | **0.000** |  | **0.000** |
| Normal | 51 (92.7) | 86.5 (5.2) |  | 72.5 (7.8) |  | 70.0 (8.0) |  |
| Abnormal | 4 (7.3) | 50.0 (25.0) |  | 0.0 (0.0) |  | 0.0 (0.0) |  |
| **JAK1** |  |  | **0.001** |  | **0.000** |  | **0.000** |
| Normal | 51 (92.7) | 88.7 (4.8) |  | 72.3 (7.8) |  | 69.8 (8.0) |  |
| Abnormal | 4 (7.3) | 33.3 (27.2) |  | 0.0 (0.0) |  | 0.0 (0.0) |  |
| **USP7** |  |  | 0.415 |  | 0.903 |  | 0.869 |
| Normal | 51 (92.7) | 82.1 (5.8) |  | 72.0 (6.7) |  | 64.1 (8.1) |  |
| Abnormal | 4 (7.3) | 100.0 (0.0) |  | 66.7 (27.2) |  | 66.7 (27.2) |  |
| **DNMT3A** |  |  | 0.319 |  | 0.608 |  | 0.703 |
| Normal | 52 (94.5) | 84.4 (5.5) |  | 66.9 (8.0) |  | 63.8 (8.3) |  |
| Abnormal | 3 (5.5) | 88.3 (15.2) |  | 66.7 (27.2) |  | 66.7 (27.2) |  |
| **NRAS** |  |  | 0.506 |  | 0.212 |  | 0.722 |
| Normal | 52 (94.5) | 82.5 (5.7) |  | 67.2 (7.8) |  | 64.2 (8.1) |  |
| Abnormal | 3 (5.5) | 100.0 (0.0) |  | 50.0 (35.4) |  | 50.0 (35.4) |  |
| **CUX1** |  |  | 0.750 |  | 0.583 |  | 0.722 |
| Normal | 52 (94.5) | 82.4 (5.1) |  | 69.8 (6.9) |  | 66.8 (7.2) |  |
| Abnormal | 3 (5.5) | 100.0 (0.0) |  | 50.0 (35.4) |  | 50.0 (35.4) |  |
| **WHSC1** |  |  | 0.436 |  | 0.619 |  | 0.244 |
| Normal | 52 (94.5) | 82.5 (5.7) |  | 64.6 (8.2) |  | 63.8 (8.3) |  |
| Abnormal | 3 (5.5) | 100.0 (0.0) |  | 50.0 (35.4) |  | 50.0 (35.4) |  |
| **ASXL2** |  |  | 0.471 |  | 0.667 |  | 0.727 |
| Normal | 52 (94.5) | 82.2 (5.8) |  | 64.2 (8.2) |  | 60.9 (8.5) |  |
| Abnormal | 3 (5.5) | 100.0 (0.0) |  | 80.0 (35.9) |  | 80.0 (35.9) |  |
| **IL-7R** |  |  | 0.484 |  | 0.310 |  | 0.290 |
| Normal | 52 (94.5) | 82.4 (5.7) |  | 65.1 (8.0) |  | 61.9 (8.3) |  |
| Abnormal | 3 (5.5) | 100.0 (0.0) |  | 100.0 (0.0) |  | 100.0 (0.0) |  |
| **TET2** |  |  | 0.507 |  | 0.827 |  | 0.871 |
| Normal | 52 (94.5) | 82.5 (5.7) |  | 68.3 (7.6) |  | 65.3 (7.9) |  |
| Abnormal | 3 (5.5) | 100.0 (0.0) |  | 100.0 (0.0) |  | 33.3 (27.2) |  |
| **BCORL1** |  |  | 0.484 |  | 0.852 |  | 0.895 |
| Normal | 52 (94.5) | 82.4 (5.7) |  | 72.5 (6.6) |  | 64.6 (8.1) |  |
| Abnormal | 3 (5.5) | 100.0 (0.0) |  | 50.0 (35.4) |  | 50.0 (35.4) |  |

T-ALL, T-cell acute lymphoblastic leukemia; ^*^significant differences about 2-year OS; ^#^ significant differences about 2-year EFS; ^&^ significant differences about 2-year DFS; Kaplan-Meier method was used to analyze the survival of each group and the differences between subgroups were evaluated using the log-rank test. Bold values indicate statistical significance at p<0.05.
