# Supplementary material for: A Novel Risk Defining System for Pediatric T-Cell Acute Lymphoblastic Leukemia From CCCG-ALL-2015 Group
Source: Front Oncol. 2022 Feb 28;12:841179. doi: 10.3389/fonc.2022.841179 (PMC8920043; doi:10.3389/fonc.2022.841179)
Supplement: Supplementary file 11 [file Table_11.docx]

**Supplementary Table 11. Univariate analysis of the relationship between various gene mutations and survival of 55 children with T-ALL**

| Variables | Overall survival (OS) | | | |  | Event-free survival (EFS) | | |  | Relapse-free survival (RFS) | | |
| --- | --- | --- | --- | --- | --- | --- | --- | --- | --- | --- | --- | --- |
|  | OR | 95%CI (OR) | | *p*-value |  | OR | 95%CI (OR) | *p*-value |  | OR | 95%CI (OR) | p-value |
| NOTCH1 | 1.170 | | 0.292-4.690 | 0.825 |  | 1.500 | 0.525-4.287 | 0.449 |  | 1.518 | 0.530-4.354 | 0.437 |
| NOTCH2 | 0.046 | | 0.000-79438.703 | 0.674 |  | 1.763 | 0.229-13.606 | 0.586 |  | 1.752 | 0.227-13.512 | 0.591 |
| FBXW7 | 2.812 | | 0.752-10.516 | 0.124 |  | 1.280 | 0.469-3.498 | 0.630 |  | 1.231 | 0.451-3.357 | 0.685 |
| NOTCH1 and FBXW7 | 2.035 | | 0.544-7.604 | 0.291 |  | 1.109 | 0.387-3.183 | 0.847 |  | 1.079 | 0.376-3.094 | 0.888 |
| KMT2D | 0.477 | | 0.060-3.827 | 0.486 |  | 0.989 | 0.320-3.054 | 0.984 |  | 1.072 | 0.348-3.296 | 0.904 |
| WT1 | 1.550 | | 0.381-6.310 | 0.540 |  | 0.811 | 0.259-2.540 | 0.719 |  | 0.840 | 0.269-2.623 | 0.765 |
| FAT1 | 0.037 | | 0.000-58.236 | 0.379 |  | 0.546 | 0.124-2.392 | 0.422 |  | 0.548 | 0.125-2.401 | 0.425 |
| CREBBP | 4.064 | | 1.090-15.153 | **0.037** |  | 3.631 | 1.371-9.613 | **0.009** |  | 4.158 | 1.580-10.942 | **0.004** |
| RELN | 3.631 | | 0.905-14.563 | 0.069 |  | 4.092 | 1.393-12.025 | **0.010** |  | 3.582 | 1.239-10.357 | **0.019** |
| PHF6 | 0.783 | | 0.098-6.268 | 0.818 |  | 0.973 | 0.219-4.318 | 0.972 |  | 0.903 | 0.205-3.977 | 0.893 |
| PTEN | 1.168 | | 0.144-9.494 | 0.885 |  | 2.288 | 0.647-8.088 | 0.199 |  | 2.134 | 0.609-7.479 | 0.236 |
| JAK3 | 1.753 | | 0.213-14.434 | 0.602 |  | 1.749 | 0.392-7.792 | 0.464 |  | 1.694 | 0.383-7.498 | 0.488 |
| DNM2 | 0.042 | | 0.000-916.252 | 0.534 |  | 1.593 | 0.355-7.153 | 0.543 |  | 1.555 | 0.348-6.939 | 0.563 |
| KRAS | 0.041 | | 0.000-633.063 | 0.517 |  | 0.041 | 0.000-50.144 | 0.379 |  | 0.041 | 0.000-35.738 | 0.355 |
| ARID1A | 0.044 | | 0.000-5236.807 | 0.601 |  | 0.044 | 0.000-244.693 | 0.478 |  | 0.044 | 0.000-192.011 | 0.465 |
| JAK2 | 1.717 | | 0.211-13.967 | 0.613 |  | 0.877 | 0.115-6.713 | 0.899 |  | 0.798 | 0.105-6.086 | 0.828 |
| TP53 | 2.337 | | 0.285-19.188 | 0.429 |  | 6.581 | 1.702-25.442 | **0.006** |  | 7.761 | 1.940-31.041 | **0.004** |
| EP300 | 19.140 | | 4.540-80.684 | **0.000** |  | 10.022 | 2.835-35.436 | **0.000** |  | 7.120 | 2.204-23.007 | **0.001** |
| EZH2 | 1.881 | | 0.231-15.306 | 0.555 |  | 0.962 | 0.126-7.329 | 0.970 |  | 0.942 | 0.124-7.151 | 0.954 |
| PRDM1 | 4.169 | | 0.841-20.673 | 0.081 |  | 10.537 | 2.999-37.028 | **0.000** |  | 7.268 | 2.255-23.432 | **0.001** |
| JAK1 | 15.574 | | 3.548-68.354 | **0.000** |  | 27.889 | 6.581-118.188 | **0.000** |  | 10.537 | 2.999-37.028 | **0.000** |
| USP7 | 0.044 | | 0.000-4487.000 | 0.596 |  | 0.882 | 0.115-6.760 | 0.904 |  | 0.844 | 0.111-6.443 | 0.870 |
| DNMT3A | 2.853 | | 0.333-24.418 | 0.339 |  | 1.706 | 0.219-13.287 | 0.610 |  | 1.491 | 0.192-11.590 | 0.703 |
| NRAS | 0.046 | | 0.000-48964.790 | 0.664 |  | 1.504 | 0.196-11.554 | 0.695 |  | 1.453 | 0.190-11.100 | 0.719 |
| CUX1 | 1.424 | | 0.163-12.454 | 0.749 |  | 1.527 | 0.339-6.874 | 0.581 |  | 1.700 | 0.384-7.515 | 0.484 |
| WHSC1 | 0.044 | | 0.000-7610.073 | 0.612 |  | 1.476 | 0.321-6.783 | 0.617 |  | 2.372 | 0.533-10.554 | 0.257 |
| ASXL2 | 1.408 | | 0.175-11.353 | 0.748 |  | 0.044 | 0.000-244.693 | 0.478 |  | 0.044 | 0.000-192.011 | 0.465 |
| IL-7R | 0.045 | | 0.000-24922.940 | 0.647 |  | 0.045 | 0.000-461.293 | 0.511 |  | 0.045 | 0.000-308.852 | 0.491 |
| TET2 | 0.048 | | 0.000-6.604E+10 | 0.832 |  | 1.250 | 0.163-9.566 | 0.830 |  | 1.181 | 0.155-8.994 | 0.873 |
| BCORL1 | 0.045 | | 0.000-24922.940 | 0.647 |  | 1.210 | 0.158-9.265 | 0.854 |  | 1.144 | 0.150-8.719 | 0.896 |

T-ALL, T-cell acute lymphoblastic leukemia; WBC, white blood cells; BM, bone marrow; PB, peripheral blood; CNS, central nervous system; Cox regression analysis was used to assess the relationship between various gene mutations and survival; Bold values indicate statistical significance at p<0.05.
