# Supplementary material for: A Novel Risk Defining System for Pediatric T-Cell Acute Lymphoblastic Leukemia From CCCG-ALL-2015 Group
Source: Front Oncol. 2022 Feb 28;12:841179. doi: 10.3389/fonc.2022.841179 (PMC8920043; doi:10.3389/fonc.2022.841179)
Supplement: Supplementary file 12 [file Table_12.docx]

**Supplementary Table 12. Multivariate analysis of the relationship between various gene mutations and survival of 55 children with T-ALL**

| Variables | Overall survival (OS) | | | |  | Event-free survival (EFS) | | |  | Relapse-free survival (RFS) | | |
| --- | --- | --- | --- | --- | --- | --- | --- | --- | --- | --- | --- | --- |
|  | OR | 95%CI (OR) | | *p*-value |  | OR | 95%CI (OR) | *p*-value |  | OR | 95%CI (OR) | p-value |
| NOTCH1 | 1.261 | | 0.310-5.134 | 0.746 |  | 1.635 | 0.558-4.788 | 0.370 |  | 1.547 | 0.529-4.520 | 0.426 |
| MRD at day 19 | 3.396 | | 1.510-7.638 | **0.003** |  | 2.613 | 1.597-4.277 | **0.000** |  | 2.358 | 1.505-3.693 | **0.000** |
| NOTCH1 | 1.048 | | 0.247-4.451 | 0.949 |  | 1.500 | 0.514-4.380 | 0.458 |  | 1.441 | 0.492-4.222 | 0.505 |
| MRD at day 46 | 2.803 | | 1.746-4.498 | **0.000** |  | 1.600 | 1.219-3.287 | **0.000** |  | 2.309 | 1.611-3.309 | **0.000** |
| NOTCH2 | 0.000 | | 0.000- | 0.991 |  | 1.369 | 0.175-10.709 | 0.765 |  | 1.346 | 0.172-10.514 | 0.777 |
| MRD at day 19 | 3.424 | | 1.545-7.591 | **0.000** |  | 2.567 | 1.587-4.154 | **0.000** |  | 2.337 | 1.501-3.639 | **0.000** |
| NOTCH2 | 0.000 | | 0.000- | 0.992 |  | 2.362 | 0.295-18.878 | 0.418 |  | 2.308 | 0.290-18.394 | 0.430 |
| MRD at day 46 | 2.757 | | 1.742-4.408 | **0.000** |  | 2.307 | 1.611-3.303 | **0.000** |  | 2.338 | 1.628-3.358 | **0.000** |
| FBXW7 | 3.077 | | 0.780-12.133 | 0.108 |  | 1.514 | 0.552-4.152 | 0.421 |  | 1.461 | 0.533-4.005 | 0.461 |
| MRD at day 19 | 3.321 | | 1.503-7.338 | **0.003** |  | 2.580 | 1.601-4.159 | **0.000** |  | 1.501 | 1.517-3.665 | **0.000** |
| FBXW7 | 4.568 | | 1.070-19.653 | **0.040** |  | 1.568 | 0.558-4.402 | 0.393 |  | 1.661 | 0.538-4.738 | 0.342 |
| MRD at day 46 | 3.245 | | 1.881-5.599 | **0.000** |  | 2.332 | 1.619-3.361 | **0.000** |  | 2.400 | 1.648-3.495 | **0.000** |
| NOTCH1 and FBXW7 | 2.050 | | 0.514-8.165 | 0.309 |  | 1.222 | 0.426-3.505 | 0.710 |  | 1.191 | 0.415-3.418 | 0.745 |
| MRD at day 19 | 3.301 | | 1.495-7.287 | **0.003** |  | 2.570 | 1.592-4.148 | **0.000** |  | 2.344 | 1.507-3.646 | **0.000** |
| NOTCH1 and FBXW7 | 2.993 | | 0.730-12.247 | 0.127 |  | 1.323 | 0.452-3.875 | 0.609 |  | 1.419 | 0.477-4.221 | 0.529 |
| MRD at day 46 | 3.036 | | 1.820-5.064 | **0.000** |  | 2.300 | 1.605-3.295 | **0.000** |  | 2.357 | 1.630-3.410 | **0.000** |
| KMT2D | 1.541 | | 0.171-13.848 | 0.700 |  | 2.795 | 0.776-10.073 | 0.116 |  | 3.118 | 0.842-11.541 | 0.089 |
| MRD at day 19 | 3.480 | | 1.548-7.823 | **0.003** |  | 2.975 | 1.732-5.110 | **0.000** |  | 2.773 | 1.641-4.689 | **0.000** |
| KMT2D | 0.497 | | 0.061-4.071 | 0.515 |  | 1.108 | 0.353-3.478 | 0.861 |  | 1.209 | 0.384-3.804 | 0.746 |
| MRD at day 46 | 2.777 | | 1.741-4.432 | **0.000** |  | 2.272 | 1.596-3.234 | **0.000** |  | 2.311 | 1.617-3.303 | **0.000** |
| WT1 | 1.084 | | 0.246-4.772 | 0.915 |  | 0.607 | 0.189-1.949 | 0.401 |  | 0.645 | 0.202-2.055 | 0.458 |
| MRD at day 19 | 3.358 | | 1.500-7.516 | **0.003** |  | 2.625 | 1.620-4.252 | **0.000** |  | 2.389 | 1.527-3.738 | **0.000** |
| WT1 | 3.130 | | 0.644-15.219 | 0.157 |  | 1.097 | 0.334-3.598 | 0.879 |  | 1.196 | 0.356-3.917 | 0.767 |
| MRD at day 46 | 3.123 | | 1.845-5.287 | **0.000** |  | 2.279 | 1.591-3.266 | **0.000** |  | 2.328 | 1.614-3.357 | **0.000** |
| FAT1 | 0.000 | | 0.000- | 0.973 |  | 0.580 | 0.131-2.571 | 0.473 |  | 0.581 | 0.131-2.571 | 0.475 |
| MRD at day 19 | 3.184 | | 1.457-6.956 | **0.004** |  | 2.542 | 1.580-4.089 | **0.000** |  | 2.321 | 1.496-3.600 | **0.000** |
| FAT1 | 0.000 | | 0.000- | 0.969 |  | 0.304 | 0.067-1.391 | 0.125 |  | 0.268 | 0.057-1.255 | 0.095 |
| MRD at day 46 | 3.657 | | 1.959-6.835 | **0.000** |  | 2.428 | 1.703-3.461 | **0.000** |  | 2.545 | 1.755-3.691 | **0.000** |
| CREBBP | 2.451 | | 0.591-9.860 | 0.219 |  | 2.637 | 0.972-7.150 | **0.057** |  | 3.072 | 1.137-8.301 | **0.027** |
| MRD at day 19 | 3.065 | | 1.436-6.541 | **0.004** |  | 2.440 | 1.526-3.902 | **0.000** |  | 2.254 | 1.443-3.522 | **0.000** |
| CREBBP | 2.166 | | 0.490-9.585 | 0.308 |  | 2.214 | 0.761-6.441 | 0.145 |  | 2.848 | 1.001-8.103 | **0.050** |
| MRD at day 46 | 2.600 | | 1.576-4.290 | **0.000** |  | 2.047 | 1.409-2.974 | **0.000** |  | 2.072 | 1.433-2.997 | **0.000** |
| RELN | 1.358 | | 0.303-6.078 | 0.689 |  | 2.042 | 0.680-6.129 | 0.203 |  | 1.878 | 0.634 -5.565 | 0.255 |
| MRD at day 19 | 3.269 | | 1.455-7.395 | **0.004** |  | 2.409 | 1.505-4.049 | **0.000** |  | 2.252 | 1.428-3.552 | **0.000** |
| RELN | 3.644 | | 0.776-17.121 | 0.101 |  | 5.041 | 1.562-16.268 | **0.007** |  | 4.277 | 1.367-13.384 | **0.013** |
| MRD at day 46 | 2.863 | | 1.729-4.740 | **0.000** |  | 2.397 | 1.641-3.501 | **0.000** |  | 2.411 | 1.651-3.520 | **0.000** |
| PHF6 | 0.349 | | 0.033-3.634 | 0.378 |  | 0.564 | 0.525-4.287 | 0.476 |  | 0.509 | 0.107-2.412 | 0.395 |
| MRD at day 19 | 3.647 | | 1.584-8.393 | **0.002** |  | 2.656 | 1.624-4.343 | **0.000** |  | 2.447 | 1.546-3.873 | **0.000** |
| PHF6 | 2.103 | | 0.220-20.150 | 0.519 |  | 1.930 | 0.395-9.444 | 0.417 |  | 1.704 | 0.356-8.155 | 0.505 |
| MRD at day 46 | 2.934 | | 1.771-4.863 | **0.000** |  | 2.359 | 1.629-3.416 | **0.000** |  | 2.373 | 1.637-3.440 | **0.000** |
| PTEN | 0.607 | | 0.074-5.004 | 0.643 |  | 1.465 | 0.410-5.233 | 0.556 |  | 1.377 | 0.388-4.888 | 0.620 |
| MRD at day 19 | 3.425 | | 1.543-7.600 | **0.002** |  | 2.541 | 1.565-4.125 | **0.000** |  | 2.312 | 1.480-3.611 | **0.000** |
| PTEN | 3.804 | | 0.362-40.023 | 0.266 |  | 4.833 | 1.210-19.307 | **0.026** |  | 4.262 | 1.095-16.579 | **0.036** |
| MRD at day 46 | 3.044 | | 1.808-5.123 | **0.000** |  | 2.513 | 1.725-3.662 | **0.000** |  | 2.520 | 1.730-3.672 | **0.000** |
| JAK3 | 0.860 | | 0.100-7.422 | 0.891 |  | 0.836 | 0.182-3.842 | 0.818 |  | 0.837 | 0.184-3.812 | 0.818 |
| MRD at day 19 | 3.399 | | 1.510-7.654 | **0.003** |  | 2.598 | 1.594-4.235 | **0.000** |  | 2.366 | 1.507-3.715 | **0.000** |
| JAK3 | 1.331 | | 0.133-13.279 | 0.808 |  | 1.447 | 0.312-6.717 | 0.637 |  | 1.586 | 0.349-7.209 | 0.550 |
| MRD at day 46 | 2.798 | | 1.741-4.498 | **0.000** |  | 2.264 | 1.588-3.226 | **0.000** |  | 2.309 | 1.612-3.306 | **0.000** |
| DNM2 | 0.000 | | 0.000- | 0.986 |  | 0.963 | 0.212-4.373 | 0.961 |  | 0.984 | 0.216-4.477 | 0.984 |
| MRD at day 19 | 3.520 | | 1.607-7.712 | **0.002** |  | 2.574 | 1.590-4.167 | **0.000** |  | 2.343 | 1.502-3.655 | **0.000** |
| DNM2 | 0.000 | | 0.000- | 0.984 |  | 0.652 | 0.136-3.117 | 0.592 |  | 0.596 | 0.123-2.891 | 0.521 |
| MRD at day 46 | 3.398 | | 1.936-5.965 | **0.000** |  | 2.334 | 1.623-3.354 | **0.000** |  | 2.395 | 1.651-3.474 | **0.000** |
| KRAS | 0.000 | | 0.000- | 0.989 |  | 0.000 | 0.000- | 0.983 |  | 0.000 | 0.000- | 0.982 |
| MRD at day 19 | 3.153 | | 1.447-6.870 | **0.004** |  | 2.441 | 1.530-3.894 | **0.000** |  | 2.234 | 1.453-3.432 | **0.000** |
| KRAS | 0.000 | | 0.000- | 0.989 |  | 0.000 | 0.000- | 0.981 |  | 0.000 | 0.000- | 0.986 |
| MRD at day 46 | 2.689 | | 1.679-4.335 | **0.000** |  | 2.178 | 1.530-3.100 | **0.000** |  | 2.205 | 1.544-3.148 | **0.000** |
| ARID1A | 0.000 | | 0.292-4.690 | 0.990 |  | 0.000 | 0.000- | 0.985 |  | 0.000 | 0.000- | 0.985 |
| MRD at day 19 | 3.297 | | 1.476-7.368 | **0.004** |  | 2.518 | 1.556-4.076 | **0.000** |  | 2.287 | 1.467-3.565 | **0.000** |
| ARID1A | 0.000 | | 0.000- | 0.991 |  | 0.000 | 0.000- | 0.985 |  | 0.000 | 0.000- | 0.984 |
| MRD at day 46 | 2.735 | | 1.702-4.394 | **0.000** |  | 2.205 | 1.549-3.138 | **0.000** |  | 2.236 | 1.566-3.192 | **0.000** |
| JAK2 | 1.644 | | 0.160-16.918 | 0.676 |  | 0.841 | 0.102-6.919 | 0.872 |  | 0.975 | 0.122-7.775 | 0.981 |
| MRD at day 19 | 3.220 | | 1.466-7.074 | **0.004** |  | 2.591 | 1.580-4.249 | **0.000** |  | 2.343 | 1.502-3.657 | **0.000** |
| JAK2 | 0.346 | | 0.035-3.453 | 0.366 |  | 0.171 | 0.019-1.554 | 0.117 |  | 0.178 | 0.020-1.571 | 0.120 |
| MRD at day 46 | 3.074 | | 1.861-5.077 | **0.000** |  | 2.715 | 1.821-4.050 | **0.000** |  | 2.720 | 1.831-4.042 | **0.000** |
| TP53 | 0.820 | | 0.099-6.766 | 0.854 |  | 4.311 | 1.045-17.789 | **0.043** |  | 5.273 | 1.210-22.976 | **0.027** |
| MRD at day 19 | 3.396 | | 1.523-7.573 | **0.003** |  | 2.527 | 1.538-4.153 | **0.000** |  | 2.285 | 1.450-3.600 | **0.000** |
| TP53 | 7.090 | | 0.680-73.938 | 0.102 |  | 17.047 | 3.551-81.833 | **0.000** |  | 20.657 | 4.166-102.439 | **0.000** |
| MRD at day 46 | 3.097 | | 1.839-5.218 | **0.000** |  | 2.704 | 1.808-4.044 | **0.000** |  | 2.763 | 1.842-4.144 | **0.000** |
| EP300 | 12.228 | | 1.263-118.431 | **0.031** |  | 3.518 | 0.752-16.453 | 0.110 |  | 4.947 | 1.068-22.926 | **0.041** |
| MRD at day 19 | 2.397 | | 1.229-4.677 | **0.010** |  | 2.264 | 1.420-3.610 | **0.001** |  | 2.084 | 1.366-3.197 | **0.001** |
| EP300 | 5.436 | | 0.430-68.710 | 0.191 |  | 1.589 | 0.247-10.229 | 0.626 |  | 2.217 | 0.372-13.225 | 0.382 |
| MRD at day 46 | 2.229 | | 1.227-4.052 | **0.009** |  | 2.113 | 1.341-3.327 | **0.001** |  | 2.073 | 1.347-3.189 | **0.001** |
| EZH2 | 0.812 | | 0.099-6.688 | 0.847 |  | 0.357 | 0.046-2.799 | 0.327 |  | 0.367 | 0.047-2.859 | 0.338 |
| MRD at day 19 | 3.395 | | 1.523-7.565 | **0.003** |  | 2.702 | 1.661-4.396 | **0.000** |  | 2.461 | 1.567-3.865 | **0.000** |
| EZH2 | 1.723 | | 0.204-14.590 | 0.617 |  | 0.801 | 0.104-6.144 | 0.831 |  | 0.821 | 0.107-6.286 | 0.850 |
| MRD at day 46 | 2.821 | | 1.747-4.558 | **0.000** |  | 2.265 | 1.597-3.214 | **0.000** |  | 2.297 | 1.613-3.272 | **0.000** |
| PRDM1 | 0.733 | | 0.292-4.690 | 0.774 |  | 3.333 | 0.866-12.833 | 0.080 |  | 3.624 | 0.928-14.147 | 0.064 |
| MRD at day 19 | 3.420 | | 1.534-7.625 | **0.003** |  | 2.456 | 1.491-4.046 | **0.000** |  | 2.215 | 1.399-3.506 | **0.001** |
| PRDM1 | 2.909 | | 0.337-25.112 | 0.331 |  | 9.399 | 2.367-37.318 | **0.001** |  | 9.428 | 2.390-37.187 | **0.001** |
| MRD at day 46 | 1.624 | | 1.763-4.772 | **0.000** |  | 2.419 | 1.643-3.561 | **0.000** |  | 2.442 | 1.655-3.603 | **0.000** |
| JAK1 | 9.206 | | 1.783-47.531 | **0.008** |  | 18.443 | 3.832-88.768 | **0.000** |  | 7.499 | 2.209-25.462 | **0.001** |
| MRD at day 19 | 3.388 | | 1.424-8.063 | **0.006** |  | 2.538 | 1.519-4.240 | **0.000** |  | 2.336 | 1.441-3.788 | **0.001** |
| JAK1 | 15.144 | | 2.272-100.841 | **0.005** |  | 31.899 | 5.572-182.615 | **0.000** |  | 15.690 | 4.067-60.528 | **0.000** |
| MRD at day 46 | 2.844 | | 1.660-4.872 | **0.000** |  | 2.304 | 1.546-3.432 | **0.000** |  | 2.488 | 1.675-3.969 | **0.000** |
| USP7 | 0.000 | | 0.000- | 0.990 |  | 0.606 | 0.078-4.685 | 0.632 |  | 0.569 | 0.074-4.365 | 0.587 |
| MRD at day 19 | 3.328 | | 1.531-7.233 | **0.002** |  | 2.570 | 1.598-4.133 | **0.000** |  | 2.349 | 1.516-3.639 | **0.000** |
| USP7 | 0.000 | | 0.000- | 0.991 |  | 0.368 | 0.045-3.006 | 0.351 |  | 0.331 | 0.040-2.742 | 0.306 |
| MRD at day 46 | 3.454 | | 1.956-6.102 | **0.000** |  | 2.391 | 1.662-3.441 | **0.000** |  | 2.460 | 1.693-3.575 | **0.000** |
| DNMT3A | 2.270 | | 0.244-21.135 | 0.471 |  | 0.814 | 0.088-7.542 | 0.856 |  | 0.749 | 0.083-6.719 | 0.796 |
| MRD at day 19 | 3.541 | | 1.488-8.425 | **0.004** |  | 2.582 | 1.594-4.183 | **0.000** |  | 2.356 | 1.511-3.673 | **0.000** |
| DNMT3A | 1.462 | | 0.148-14.478 | 0.746 |  | 0.999 | 0.122-8.213 | 1.000 |  | 0.713 | 0.087-5.872 | 0.753 |
| MRD at day 46 | 2.786 | | 1.731-4.483 | **0.000** |  | 2.266 | 1.593-3.223 | **0.000** |  | 2.318 | 1.622-3.311 | **0.000** |
| NRAS | 0.000 | | 0.000- | 0.992 |  | 1.162 | 0.162-9.628 | 0.831 |  | 1.188 | 0.155-9.095 | 0.868 |
| MRD at day 19 | 3.274 | | 1.495-7.169 | **0.003** |  | 2.578 | 1.590-4.180 | **0.000** |  | 2.345 | 1.504-3.656 | **0.000** |
| NRAS | 0.000 | | 0.000- | 0.989 |  | 3.078 | 0.371-25.522 | 0.298 |  | 2.840 | 0.349-23.119 | 0.329 |
| MRD at day 46 | 2.755 | | 1.716-4.426 | **0.000** |  | 2.342 | 1.630-3.365 | **0.000** |  | 2.367 | 1.645-3.407 | **0.000** |
| CUX1 | 0.604 | | 0.045-8.089 | 0.703 |  | 1.170 | 0.254-5.403 | 0.840 |  | 1.254 | 0.274-5.740 | 0.771 |
| MRD at day 19 | 3.486 | | 1.524-7.971 | **0.003** |  | 2.560 | 1.581-4.144 | **0.000** |  | 2.325 | 1.491-3.624 | **0.000** |
| CUX1 | 3.264 | | 0.298-35.799 | 0.333 |  | 2.411 | 0.493-11.785 | 0.277 |  | 2.863 | 0.602-13.612 | 0.186 |
| MRD at day 46 | 2.930 | | 1.792-4.790 | **0.000** |  | 2.333 | 1.631-3.337 | **0.000** |  | 2.393 | 1.662-3.445 | **0.000** |
| WHSC1 | 0.000 | | 0.000- | 0.991 |  | 2.539 | 0.518-12.450 | 0.251 |  | 4.254 | 0.896-20.195 | 0.068 |
| MRD at day 19 | 3.295 | | 1.476-7.357 | **0.004** |  | 2.663 | 1.629-4.355 | **0.000** |  | 2.456 | 1.560-3.865 | **0.000** |
| WHSC1 | 0.000 | | 0.000- | 0.990 |  | 2.288 | 0.456-11.473 | 0.314 |  | 4.267 | 0.885-20.571 | 0.071 |
| MRD at day 46 | 2.749 | | 1.712-4.413 | **0.000** |  | 2.326 | 1.627-3.323 | **0.000** |  | 2.425 | 1.683-3.496 | **0.000** |
| ASXL2 | 1.101 | | 0.135-8.958 | 0.928 |  | 0.872 | 0.193-3.937 | 0.858 |  | 0.885 | 0.196-3.990 | 0.873 |
| MRD at day 19 | 3.376 | | 1.511-7.542 | **0.003** |  | 2.579 | 1.595-4.170 | **0.000** |  | 2.351 | 1.508-3.666 | **0.000** |
| ASXL2 | 2.584 | | 0.288-23.195 | 0.397 |  | 1.925 | 0.407-9.102 | 0.409 |  | 2.158 | 0.456-10.008 | 0.326 |
| MRD at day 46 | 2.918 | | 1.776-4.793 | **0.000** |  | 2.315 | 1.618-3.312 | **0.000** |  | 2.365 | 1.643-3.404 | **0.000** |
| IL-7R | 0.000 | | 0.000- | 0.989 |  | 0.000 | 0.000- | 0.983 |  | 0.000 | 0.000- | 0.982 |
| MRD at day 19 | 3.430 | | 1.540-7.642 | **0.003** |  | 2.683 | 1.646-4.373 | **0.000** |  | 2.454 | 1.560-3.861 | **0.000** |
| IL-7R | 0.000 | | 0.000- | 0.992 |  | 0.000 | 0.000- | 0.985 |  | 0.000 | 0.000- | 0.984 |
| MRD at day 46 | 2.750 | | 1.712-4.417 | **0.000** |  | 2.218 | 1.559-3.154 | **0.000** |  | 2.247 | 1.574-3.207 | **0.000** |
| TET2 | 0.000 | | 0.000- | 0.992 |  | 1.106 | 0.132-7.820 | 0.988 |  | 0.916 | 0.120-6.998 | 0.933 |
| MRD at day 19 | 3.274 | | 1.495-7.169 | **0.003** |  | 2.572 | 1.590-4.158 | **0.000** |  | 2.342 | 1.506-3.642 | **0.000** |
| TET2 | 0.000 | | 0.000- | 0.989 |  | 2.464 | 0.300-20.273 | 0.402 |  | 2.234 | 0.276-18.097 | 0.451 |
| MRD at day 46 | 2.755 | | 1.716-4.426 | **0.000** |  | 2.329 | 1.622-3.344 | **0.000** |  | 2.354 | 1.636-3.387 | **0.000** |
| BCORL1 | 0.000 | | 0.000- | 0.990 |  | 0.837 | 0.108-6.493 | 0.865 |  | 0.818 | 0.106-6.326 | 0.848 |
| MRD at day 19 | 3.399 | | 1.539-7.506 | **0.002** |  | 2.575 | 1.594-4.160 | **0.000** |  | 2.347 | 1.508-3.652 | **0.000** |
| BCORL1 | 0.000 | | 0.000- | 0.989 |  | 0.397 | 0.048-3.289 | 0.392 |  | 0.356 | 0.042-2.987 | 0.341 |
| MRD at day 46 | 3.478 | | 1.969-6.144 | **0.000** |  | 2.394 | 1.661-3.452 | **0.000** |  | 2.464 | 1.692-3.587 | **0.000** |

T-ALL, T-cell acute lymphoblastic leukemia; WBC, white blood cells; BM, bone marrow; PB, peripheral blood; CNS, central nervous system; MRD, minimal residual disease; Cox regression analysis was used to assess the relationship between various gene mutations and survival; Bold values indicate statistical significance at p<0.05.
