# Supplementary material for: A Novel Risk Defining System for Pediatric T-Cell Acute Lymphoblastic Leukemia From CCCG-ALL-2015 Group
Source: Front Oncol. 2022 Feb 28;12:841179. doi: 10.3389/fonc.2022.841179 (PMC8920043; doi:10.3389/fonc.2022.841179)
Supplement: Supplementary file 13 [file Table_13.docx]

Supplementary Table 13. Comparison of basic data between Targeted-NGS group and Non-Targeted-NGS group.

| Characteristics | Targeted-NGS group  N (%) | Non-Targeted-NGS group  N (%) | *P* value |
| --- | --- | --- | --- |
| **Total** | 55 (100.0) | 50 (100.0) |  |
| **Gender** |  |  | 0.514 |
| Male | 38 (69.1) | 38 (76.0) |  |
| Female | 17 (30.9) | 12 (24.0) |  |
| **Age (years)** |  |  | 0.561 |
| <3 | 5 (9.1) | 4 (8.0) |  |
| ≥3 | 50 (90.9) | 46 (92.0) |  |
| Median (range) | 8.8 (1.0-15.0) | 8.9 (1.4-15.0) |  |
| **Initial WBC (×10^9^/L)** |  |  | 0.538 |
| <50 | 20 (36.4) | 18 (36.0) |  |
| ≥50 | 34 (61.8) | 32 (64.0) |  |
| Unknown | 1 (1.8) | 0 (0.0) |  |
| Median (range) | 140.6 (0.3-631.05) | 140.6 (1.7-800.67) |  |
| **Initial Hemoglobin (g/L)** |  |  | 0.695 |
| <100 | 25 (45.5) | 26 (52.0) |  |
| ≥100 | 29 (52.7) | 24 (48.0) |  |
| Unknown | 1 (1.8) | 0 (0.0) |  |
| Median (range) | 104.1 (42.0-152.0) | 92.6 (39.0-138.0) |  |
| **Initial Platelet (×10^9^/L)** |  |  | 0.521 |
| <20 | 4 (7.3) | 2 (4.0) |  |
| ≥20 and <100 | 36 (65.5) | 34 (68.0) |  |
| ≥100 | 14 (25.5) | 14 (28.0) |  |
| Unknown | 1 (1.8) | 0 (0.0) |  |
| Median (range) | 92.7 (15.0-357.0) | 81.1 (4.0-324.0) |  |
| **Initial blasts in BM (%)** |  |  | 0.330 |
| <50 | 7 (12.7) | 3 (6.0) |  |
| ≥50 and <80 | 13 (23.6) | 9 (18.0) |  |
| ≥80 | 35 (63.6) | 38 (76.0) |  |
| Median (range) | 78.3 (30.0-99.0) | 82.9 (30.0-99.5) |  |
| **Initial blasts in PB (%)** |  |  | 0.252 |
| <20 | 16 (29.1) | 8 (16.0) |  |
| ≥20 and <80 | 18 (32.7) | 19 (38.0) |  |
| ≥80 | 20 (36.4) | 23 (26.0) |  |
| Unknown | 1 (1.8) | 0 (0.0) |  |
| Median (range) | 52.5 (0.0-98.5) | 63.1 (0.0-99.0) |  |
| **CNS involvement** |  |  | 0.539 |
| CNS1 | 49 (89.1) | 40 (80.0) |  |
| CNS2/3 | 5 (9.1) | 7 (14.0) |  |
| Unknown | 1 (1.8) | 3 (6.0) |  |
| **Mediastinal mass** |  |  | 0.077 |
| Present | 31 (56.4) | 20 (40.0) |  |
| Absent | 22 (40.0) | 30 (60.0) |  |
| Unknown | 2 (3.6) | 0 (0.0) |  |
| **Hepatomegalia** |  |  | 0.187 |
| Absent | 28 (50.9) | 25 (50.0) |  |
| Mild (less than 5cm under the ribs) | 26 (47.3) | 22 (44.0) |  |
| Severe (more than or equal to 5cm under the ribs) | 0 (0.0) | 3 (6.0) |  |
| Unknown | 1 (1.8) | 0 (0.0) |  |
| **Splenomegalia** |  |  | 0.499 |
| Absent | 26 (47.3) | 19 (38.0) |  |
| Mild (less than 5cm under the ribs) | 10 (18.2) | 9 (18.0) |  |
| Severe (more than or equal to 5cm under the ribs) | 18 (32.7) | 22 (44.0) |  |
| Unknown | 1 (1.8) | 0 (0.0) |  |
| **Immunophenotype** |  |  | 0.968 |
| ETP-ALL | 12 (21.8) | 14 (28.0) |  |
| Early non-ETP-ALL | 18 (32.7) | 15 (30.0) |  |
| Cortex T-ALL | 10 (18.2) | 8 (16.0) |  |
| Medullary T-ALL | 13 (23.6) | 13 (26.0) |  |
| Unknown | 2 (3.6) | 0 (0.0) |  |
| **SIL-TAL1 translocation** |  |  | 0.483 |
| Present | 10 (18.2) | 12 (24.0) |  |
| Absent | 45 (81.8) | 38 (76.0) |  |
| **Myc positive** |  |  | 0.619 |
| Present | 3 (5.5) | 1 (2.0) |  |
| Absent | 52 (94.5) | 49 (98.0) |  |
| **MLL rearrangement** |  |  | 0.667 |
| Present | 2 (3.6) | 3 (6.0) |  |
| Absent | 53 (96.4) | 47 (94.0) |  |
| **CDKN2A/CEP9** |  |  | 0.214 |
| Present | 21 (38.2) | 13 (26.0) |  |
| Absent | 34 (61.8) | 37 (74.0) |  |
| **karyotype** |  |  | 0.485 |
| Normal | 32 (58.2) | 28 (56.0) |  |
| Structure abnormal | 4 (7.3) | 7 (14.0) |  |
| Numerical abnormal | 14 (25.5) | 11 (22.0) |  |
| Failure or Missing | 5 (9.1) | 4 (8.0) |  |

Targeted-NGS group, patients who underwent targeted next-generation sequencing for 112 gene mutations; Non-Targeted-NGS group, patients who did not undergo targeted next-generation sequencing for 112 gene mutations; WBC, white blood cells; BM, bone marrow; PB, peripheral blood; CNS, central nervous system; ETP, early T-cell precursor; T-ALL, T-cell acute lymphoblastic leukemia; Continuous and categorical variables were compared using the Mann-Whitney U test and chi-square test, respectively.
