# Supplementary material for: A Novel Risk Defining System for Pediatric T-Cell Acute Lymphoblastic Leukemia From CCCG-ALL-2015 Group
Source: Front Oncol. 2022 Feb 28;12:841179. doi: 10.3389/fonc.2022.841179 (PMC8920043; doi:10.3389/fonc.2022.841179)
Supplement: Supplementary file 14 [file Table_14.docx]

**Supplementary Table 14. 112 gene mutations are divided into 10 groups according to the different signaling pathways involved**

| **Pathways** | **Gene** |
| --- | --- |
| NOTCH signaling pathway | NOTCH2, NOTCH1, FBXW7 |
| Ras/Protein phosphatase/MARK/PI3K signaling pathway | NRAS, KRAS, NF1, CBL, TP53, DNM2, PTEN, AKT, ETV6, PLCG1, PLCG2, CCND3, ARID1A, BRAF, USP7, DDX3X, BCOR, TRAF3, ANKRD26, ATM, KIT, CXCR4, TEL2 |
| Transcription factor/regulation | CREBBP, RUNX1, CEBPA, PHF6, WT1, PRDM1, GATA2, GATA3, SETBP1, BCORL1, TERT, KMT2A |
| Epigenetic modulators | TET2, EP300, EZH2, DNMT3A, DNMT3B, ASXL1, KMT2D, WHSC1, CUX1, RELN, SETD2 |
| Jak-Stat Signaling Pathway | JAK1, JAK2, JAK3, IL7R, STAT3, STAT5B, CRLF2, SUZ12, RPL10, CALR, FOXO1 |
| Splicing and mRNA processing regulation | DIS3, SF3A1, NT5C2, PALB2, ECT2L, ACD, SMC3, ATG2B, DDX41 |
| NF-KB pathway | IKZF1, MUM1, RBBP6, CARD11, TRAF3 |
| Wnt/β-Catenin pathway | FAT1, TCF3, MAX, KDM6A, PIK3CD |
| Receptor/Nonreceptor tryosine kinase signaling pathway | FGFR3, SH2B3, CSF3R, EPHA7 |
| Cyclins and Cell Cycle Regulation | CDKN1B, RAD21, MPL, BRINP3, SMC1A |
