# Supplementary material for: A Novel Risk Defining System for Pediatric T-Cell Acute Lymphoblastic Leukemia From CCCG-ALL-2015 Group
Source: Front Oncol. 2022 Feb 28;12:841179. doi: 10.3389/fonc.2022.841179 (PMC8920043; doi:10.3389/fonc.2022.841179)
Supplement: Supplementary file 15 [file Table_15.docx]

Supplementary Table 15. The main signaling pathways of 55 children with T-ALL and the comparisons of their survival.

| The main signaling pathways | N (%) | 2-year OS (SE) | *P^*^* value | 2-year EFS (SE) | *P^#^* value | 2-year DFS (SE) | *P^&^* value |
| --- | --- | --- | --- | --- | --- | --- | --- |
| **Total** | 55 (100.0) | 83.4 (5.4) |  | 66.9 (7.7) |  | 64.0 (7.9) |  |
| **NOTCH signaling pathway** |  |  | 0.824 |  | 0.442 |  | 0.431 |
| Normal | 18 (32.7) | 82.4 (9.2) |  | 71.3 (10.9) |  | 71.3 (10.9) |  |
| Abnormal | 37 (67.3) | 84.1 (6.7) |  | 63.5 (11.0) |  | 57.7 (11.7) |  |
| **Ras/Protein phosphatase/MARK/PI3K signaling**  **Pathway** |  |  | **0.032** |  | 0.510 |  | 0.408 |
| Normal | 22 (40.0) | 70.0 (10.72) |  | 64.6 (11.2) |  | 58.5 (11.7) |  |
| Abnormal | 33 (60.0) | 92.3 (5.2) |  | 70.8 (9.0) |  | 70.0 (9.1) |  |
| **Transcription factor/regulation** |  |  | **0.027** |  | **0.009** |  | **0.007** |
| Normal | 27 (49.1) | 96.3 (3.6) |  | 85.5 (8.3) |  | 85.5 (8.3) |  |
| Abnormal | 28 (50.9) | 73.6 (8.6) |  | 52.2 (10.8) |  | 47.2 (11.1) |  |
| **Epigenetic modulators** |  |  | 0.131 |  | 0.159 |  | 0.107 |
| Normal | 31 (56.4) | 88.1 (6.5) |  | 76.1 (7.9) |  | 74.8 (8.4) |  |
| Abnormal | 24 (43.6) | 77.1 (9.1) |  | 54.5 (13.6) |  | 48.3 (14.2) |  |
| **Jak-Stat Signaling Pathway** |  |  | 0.636 |  | 0.346 |  | 0.277 |
| Normal | 32 (58.2) | 78.7 (7.8) |  | 64.9 (9.2) |  | 59.7 (9.8) |  |
| Abnormal | 23 (41.8) | 60.4 (25.0) |  | 67.5 (14.3) |  | 67.5 (14.3) |  |
| **Splicing and mRNA processing regulation** |  |  | 0.747 |  | 0.570 |  | 0.594 |
| Normal | 46 (83.6) | 82.9 (6.0) |  | 63.1 (8.7) |  | 59.5 (9.0) |  |
| Abnormal | 9 (16.4) | 87.5 (11.7) |  | 87.5 (11.7) |  | 87.5 (11.7) |  |
| **NF-KB pathway** |  |  | 0.674 |  | 0.741 |  | 0.780 |
| Normal | 48 (87.3) | 80.7 (6.2) |  | 69.6 (7.2) |  | 66.3 (7.6) |  |
| Abnormal | 7 (12.7) | 100.0 (0.0) |  | 42.9 (31.0) |  | 42.9 (31.0) |  |
| **Wnt/β-Catenin pathway** |  |  | 0.335 |  | 0.736 |  | 0.688 |
| Normal | 49 (89.1) | 81.6 (5.9) |  | 66.8 (7.9) |  | 63.6 (8.2) |  |
| Abnormal | 6 (10.9) | 100.0 (0.0) |  | 75.0 (21.7) |  | 75.0 (21.7) |  |
| **Receptor/Nonreceptor tryosine kinase signaling pathway** |  |  | 0.899 |  | 0.648 |  | 0.583 |
| Normal | 50 (90.9) | 84.3 (5.5) |  | 65.2 (8.5) |  | 61.7 (8.9) |  |
| Abnormal | 5 (9.1) | 75.0 (21.7) |  | 75.0 (21.7) |  | 75.0 (21.7) |  |
| **Cyclins and Cell Cycle Regulation** |  |  | 0.348 |  | 0.188 |  | 0.172 |
| Normal | 50 (90.9) | 81.7 (5.9) |  | 64.0 (8.1) |  | 60.8 (8.4) |  |
| Abnormal | 5 (9.1) | 100.0 (0.0) |  | 100.0 (0.0) |  | 100.0 (0.0) |  |

T-ALL, T-cell acute lymphoblastic leukemia; ^*^significant differences about 2-year OS; ^#^ significant differences about 2-year EFS; ^&^ significant differences about 2-year DFS; Kaplan-Meier method was used to analyze the survival of each group and the differences between subgroups were evaluated using the log-rank test. Bold values indicate statistical significance at p<0.05.

Genes included in “NOTCH signaling pathway”: NOTCH2, NOTCH1, FBXW7;

Genes included in “Ras/Protein phosphatase/MARK/PI3K signaling Pathway”: NRAS, KRAS, NF1, CBL, TP53, DNM2, PTEN, AKT, ETV6, PLCG1, PLCG2, CCND3, ARID1A, BRAF, USP7, DDX3X, BCOR, TRAF3, ANKRD26, ATM, KIT, CXCR4, TEL2;

Genes included in “Transcription factor/regulation”: CREBBP, RUNX1, CEBPA, PHF6, WT1, PRDM1, GATA2, GATA3, SETBP1, BCORL1, TERT, KMT2A;

Genes included in “Epigenetic modulators”: TET2, EP300, EZH2, DNMT3A, DNMT3B, ASXL1, KMT2D, WHSC1, CUX1, RELN, SETD2;

Genes included in “Jak-Stat Signaling Pathway”: JAK1, JAK2, JAK3, IL7R, STAT3, STAT5B, CRLF2, SUZ12, RPL10, CALR, FOXO1;

Genes included in “Splicing and mRNA processing regulation”: DIS3, SF3A1, NT5C2, PALB2, ECT2L, ACD, SMC3, ATG2B;

Genes included in “NF-KB pathway”: IKZF1, MUM1, RBBP6, PLCG2, CARD11, TRAF3;

Genes included in “Wnt/β-Catenin pathway”: FAT1, TCF3, MAX, DDX41, KDM6A, PIK3CD;

Genes included in “Receptor/Nonreceptor tryosine kinase signaling pathway”: FGFR3, SH2B3, CSF3R, EPHA7;

Genes included in “Cyclins and Cell Cycle Regulation”: CDKN1B, RAD21, MPL, BRINP3, SMC1A.

**Supplementary Table 16. Univariate analysis of the relationship between signaling pathways and survival of 55 children with T-ALL**

| Variables | Overall survival (OS) | | | |  | Event-free survival (EFS) | | |  | Relapse-free survival (RFS) | | |
| --- | --- | --- | --- | --- | --- | --- | --- | --- | --- | --- | --- | --- |
|  | OR | 95%CI (OR) | | *p*-value |  | OR | 95%CI (OR) | *p*-value |  | OR | 95%CI (OR) | p-value |
| NOTCH signaling pathway | 1.170 | | 0.292-4.690 | 0.825 |  | 1.500 | 0.525-4.287 | 0.449 |  | 1.518 | 0.530-4.354 | 0.437 |
| Ras/Protein phosphatase/MARK/PI3K signaling  Pathway | 0.206 | | 0.042-1.014 | 0.052 |  | 0.723 | 0.271-1.927 | 0.516 |  | 0.669 | 0.254-1.759 | 0.415 |
| Transcription factor/regulation | 7.356 | | 0.917-58.987 | 0.060 |  | 4.492 | 1.290-15.636 | **0.018** |  | 4.675 | 1.342-16.281 | **0.015** |
| Epigenetic modulators | 2.789 | | 0.693-11.228 | 0.149 |  | 1.966 | 0.747-5.173 | 0.171 |  | 2.158 | 0.821-5.677 | 0.119 |
| Jak-Stat Signaling Pathway | 0.717 | | 0.179-2.870 | 0.639 |  | 0.610 | 0.214-1.742 | 0.356 |  | 0.567 | 0.199-1.613 | 0.287 |
| Splicing and mRNA processing regulation | 0.712 | | 0.089-5.694 | 0.749 |  | 0.657 | 0.150-2.879 | 0.577 |  | 0.673 | 0.154-2.947 | 0.599 |
| NF-KB pathway | 0.558 | | 0.069-4.499 | 0.584 |  | 0.687 | 0.157-3.012 | 0.618 |  | 0.699 | 0.160-3.057 | 0.634 |
| Wnt/β-Catenin pathway | 0.042 | | 0.000-916.252 | 0.534 |  | 0.709 | 0.093-5.430 | 0.741 |  | 0.664 | 0.087-5.059 | 0.693 |
| Receptor/Nonreceptor tryosine kinase signaling pathway | 1.144 | | 0.143-9.178 | 0.899 |  | 0.628 | 0.082-4.795 | 0.654 |  | 0.573 | 0.075-4.359 | 0.590 |
| Cyclins and Cell Cycle Regulation | 0.042 | | 0.000-1158.295 | 0.544 |  | 0.042 | 0.000-67.826 | 0.401 |  | 0.042 | 0.000-50.433 | 0.381 |

T-ALL, T-cell acute lymphoblastic leukemia; Cox regression analysis was used to assess the relationship between various gene mutations and survival; Bold values indicate statistical significance at p<0.05.

Genes included in “NOTCH signaling pathway”: NOTCH2, NOTCH1, FBXW7;

Genes included in “Ras/Protein phosphatase/MARK/PI3K signaling Pathway”: NRAS, KRAS, NF1, CBL, TP53, DNM2, PTEN, AKT, ETV6, PLCG1, CCND3, ARID1A, BRAF, USP7, DDX3X, BCOR, TRAF3, ANKRD26, ATM, KIT, CXCR4, TEL2;

Genes included in “Transcription factor/regulation”: CREBBP, RUNX1, CEBPA, PHF6, WT1, PRDM1, GATA2, GATA3, SETBP1, BCORL1, TERT, KMT2A;

Genes included in “Epigenetic modulators”: TET2, EP300, EZH2, DNMT3A, DNMT3B, ASXL1, KMT2D, WHSC1, CUX1, RELN, SETD2;

Genes included in “Jak-Stat Signaling Pathway”: JAK1, JAK2, JAK3, IL7R, STAT3, STAT5B, CRLF2, SUZ12, RPL10, CALR, FOXO1;

Genes included in “Splicing and mRNA processing regulation”: DIS3, SF3A1, NT5C2, PALB2, ECT2L, ACD, SMC3, ATG2B;

Genes included in “NF-KB pathway”: IKZF1, MUM1, RBBP6, PLCG2, CARD11, TRAF3;

Genes included in “Wnt/β-Catenin pathway”: FAT1, TCF3, MAX, DDX41, KDM6A, PIK3CD;

Genes included in “Receptor/Nonreceptor tryosine kinase signaling pathway”: FGFR3, SH2B3, CSF3R, EPHA7;

Genes included in “Cyclins and Cell Cycle Regulation”: CDKN1B, RAD21, MPL, BRINP3, SMC1A.

**Supplementary Table 17. Multivariate analysis of the relationship between various signaling pathways and survival of 55 children with T-ALL**

| Variables | Overall survival (OS) | | | |  | Event-free survival (EFS) | | |  | Relapse-free survival (RFS) | | |
| --- | --- | --- | --- | --- | --- | --- | --- | --- | --- | --- | --- | --- |
|  | OR | 95%CI (OR) | | *p*-value |  | OR | 95%CI (OR) | *p*-value |  | OR | 95%CI (OR) | p-value |
| NOTCH signaling pathway | 1.360 | | 0.334-5.532 | 0.668 |  | 1.773 | 0.607-5.177 | 0.295 |  | 1.703 | 0.584-4.969 | 0.330 |
| MRD at day 19 | 3.389 | | 1.511-7.599 | **0.003** |  | 2.620 | 1.607-4.274 | **0.000** |  | 2.368 | 1.516-3.699 | **0.000** |
| NOTCH signaling pathway | 1.048 | | 0.247-4.451 | 0.949 |  | 1.475 | 0.506-4.299 | 0.477 |  | 1.414 | 0.484-4.133 | 0.527 |
| MRD at day 46 | 2.803 | | 1.746-4.498 | **0.000** |  | 2.292 | 1.600-3.284 | **0.000** |  | 2.308 | 1.611-3.307 | **0.000** |
| Ras/Protein phosphatase/MARK/PI3K signaling  Pathway | 0.255 | | 0.050-1.296 | 0.069 |  | 1.057 | 0.388-2.882 | 0.914 |  | 0.762 | 0.283-2.054 | 0.591 |
| MRD at day 19 | 3.101 | | 1.498-6.420 | **0.000** |  | 2.585 | 1.580-4.227 | **0.000** |  | 2.297 | 1.481-3.563 | **0.000** |
| Ras/Protein phosphatase/MARK/PI3K signaling  Pathway | 0.386 | | 0.074-2.012 | 0.190 |  | 1.399 | 0.510-3.839 | 0.514 |  | 0.985 | 0.360-2.697 | 0.976 |
| MRD at day 46 | 2.722 | | 1.650-4.491 | **0.000** |  | 2.334 | 1.627-3.348 | **0.000** |  | 2.296 | 1.593-3.309 | **0.000** |
| Transcription factor/regulation | 3.146 | | 0.359-27.602 | 0.301 |  | 3.004 | 0.833-10.842 | 0.093 |  | 3.255 | 0.901-11.759 | 0.072 |
| MRD at day 19 | 2.833 | | 1.293-6.207 | **0.009** |  | 2.344 | 1.456-3.773 | **0.000** |  | 2.138 | 1.378-3.317 | **0.001** |
| Transcription factor/regulation | 4.968 | | 0.581-42.457 | **0.143** |  | 3.564 | 0.997-12.734 | 0.050 |  | 3.994 | 1.123-14.199 | **0.032** |
| MRD at day 46 | 2.504 | | 1.565-4.008 | **0.000** |  | 2.332 | 1.459-2.928 | **0.000** |  | 2.132 | 1.502-3.026 | **0.000** |
| Epigenetic modulators | 1.503 | | 0.346-6.525 | 0.587 |  | 1.103 | 0.400-3.042 | 0.849 |  | 1.255 | 0.455-3.456 | 0.661 |
| MRD at day 19 | 3.198 | | 1.430-7.154 | **0.005** |  | 2.536 | 1.541-4.173 | **0.000** |  | 2.273 | 1.439-3.590 | **0.000** |
| Epigenetic modulators | 2.117 | | 0.485-9.232 | 0.318 |  | 1.609 | 0.597-4.342 | 0.347 |  | 1.847 | 0.684-4.987 | 0.226 |
| MRD at day 46 | 2.739 | | 1.680-4.467 | **0.000** |  | 2.206 | 1.548-3.145 | **0.000** |  | 2.243 | 1.567-3.211 | **0.000** |
| Jak-Stat Signaling Pathway | 0.440 | | 0.087-2.237 | 0.322 |  | 0.451 | 0.149-1.369 | 0.160 |  | 0.459 | 0.154-1.365 | 0.161 |
| MRD at day 19 | 3.851 | | 1.520-9.752 | **0.004** |  | 2.839 | 1.645-4.900 | **0.000** |  | 2.521 | 1.546-4.113 | **0.000** |
| Jak-Stat Signaling Pathway | 0.110 | | 0.015-0.800 | **0.029** |  | 0.167 | 0.040-0.676 | **0.012** |  | 0.184 | 0.050-0.677 | **0.011** |
| MRD at day 46 | 4.507 | | 2.217-9.163 | **0.000** |  | 3.230 | 1.596-5.193 | **0.000** |  | 3.165 | 1.999-5.012 | **0.000** |
| Splicing and mRNA processing regulation | 1.329 | | 0.146-12.094 | 0.801 |  | 1.085 | 0.241-4.879 | 0.915 |  | 1.290 | 0.287-5.796 | 0.740 |
| MRD at day 19 | 3.325 | | 1.524-7.255 | **0.003** |  | 2.572 | 1.596-4.144 | **0.000** |  | 2.360 | 1.522-3.660 | **0.000** |
| Splicing and mRNA processing regulation | 0.422 | | 0.044-4.034 | 0.454 |  | 0.442 | 0.092-2.118 | 0.307 |  | 0.503 | 0.107-2.372 | 0.385 |
| MRD at day 46 | 2.950 | | 1.790-4.862 | **0.000** |  | 2.410 | 1.646-3.528 | **0.000** |  | 2.395 | 1.647-3.484 | **0.000** |
| NF-KB pathway | 0.338 | | 0.032-3.509 | 0.363 |  | 0.745 | 0.168-3.303 | 0.698 |  | 0.766 | 0.173-3.380 | 0.725 |
| MRD at day 19 | 3.603 | | 1.565-8.297 | **0.003** |  | 2.569 | 1.589-4.154 | **0.000** |  | 2.342 | 1.503-3.648 | **0.000** |
| NF-KB pathway | 1.389 | | 0.144-13.399 | 0.776 |  | 1.201 | 0.255-5.664 | 0.817 |  | 1.258 | 0.267-5.918 | 0.772 |
| MRD at day 46 | 2.863 | | 1.735-4.725 | **0.000** |  | 2.291 | 1.591-3.297 | **0.000** |  | 2.332 | 1.611-3.375 | **0.000** |
| Wnt/β-Catenin pathway^8^ | 0.000 | | 0.000-. | 0.986 |  | 0.763 | 0.099-5.896 | 0.795 |  | 0.729 | 0.095-5.612 | 0.762 |
| MRD at day 19 | 3.365 | | 1.518-7.456 | **0.003** |  | 2.571 | 1.590-4.156 | **0.000** |  | 2.342 | 1.504-3.646 | **0.000** |
| Wnt/β-Catenin pathway | 0.000 | | 0.000-. | 0.983 |  | 0.226 | 0.028-1.859 | 0.167 |  | 0.185 | 0.022-1.547 | 0.119 |
| MRD at day 46 | 3.826 | | 2.023-7.235 | **0.000** |  | 2.500 | 1.736-3.599 | **0.000** |  | 2.638 | 1.797-3.873 | **0.000** |
| Receptor/Nonreceptor tryosine kinase signaling pathway | 1.512 | | 0.183-12.465 | 0.701 |  | 1.024 | 0.130-8.057 | 0.982 |  | 0.941 | 0.120 -7.390 | 0.954 |
| MRD at day 19 | 3.406 | | 1.520-7.634 | **0.003** |  | 2.573 | 1.583-4.184 | **0.000** |  | 2.337 | 1.493-3.657 | **0.000** |
| Receptor/Nonreceptor tryosine kinase signaling pathway | 2.948 | | 0.307-28.291 | 0.349 |  | 1.053 | 0.131-8.497 | 0.961 |  | 0.926 | 0.116-7.402 | 0.942 |
| MRD at day 46 | 2.961 | | 1.795-4.886 | **0.000** |  | 2.270 | 1.588-3.244 | **0.000** |  | 2.293 | 1.600-3.287 | **0.000** |
| Cyclins and Cell Cycle Regulation | 0.000 | | 0.000-. | 0.991 |  | 0.000 | 0.000-. | 0.986 |  | 0.000 | 0.000-. | 0.984 |
| MRD at day 19 | 3.209 | | 1.441-7.146 | **0.004** |  | 2.439 | 1.515-3.925 | **0.000** |  | 2.221 | 1.434-3.440 | **0.000** |
| Cyclins and Cell Cycle Regulation | 0.000 | | 0.000-. | 0.990 |  | 0.000 | 0.000-. | 0.982 |  | 0.000 | 0.000-. | 0.987 |
| MRD at day 46 | 2.711 | | 1.686-4.358 | **0.000** |  | 2.183 | 1.534-3.108 | **0.000** |  | 2.211 | 1.548-3.159 | **0.000** |

T-ALL, T-cell acute lymphoblastic leukemia; MRD, minimal residual disease; Cox regression analysis was used to assess the relationship between various gene mutations and survival; Bold values indicate statistical significance at p<0.05.

Genes included in “NOTCH signaling pathway”: NOTCH2, NOTCH1, FBXW7;

Genes included in “Ras/Protein phosphatase/MARK/PI3K signaling Pathway”: NRAS, KRAS, NF1, CBL, TP53, DNM2, PTEN, AKT, ETV6, PLCG1, CCND3, ARID1A, BRAF, USP7, DDX3X, BCOR, TRAF3, ANKRD26, ATM, KIT, CXCR4, TEL2;

Genes included in “Transcription factor/regulation”: CREBBP, RUNX1, CEBPA, PHF6, WT1, PRDM1, GATA2, GATA3, SETBP1, BCORL1, TERT, KMT2A;

Genes included in “Epigenetic modulators”: TET2, EP300, EZH2, DNMT3A, DNMT3B, ASXL1, KMT2D, WHSC1, CUX1, RELN, SETD2;

Genes included in “Jak-Stat Signaling Pathway”: JAK1, JAK2, JAK3, IL7R, STAT3, STAT5B, CRLF2, SUZ12, RPL10, CALR, FOXO1;

Genes included in “Splicing and mRNA processing regulation”: DIS3, SF3A1, NT5C2, PALB2, ECT2L, ACD, SMC3, ATG2B;

Genes included in “NF-KB pathway”: IKZF1, MUM1, RBBP6, PLCG2, CARD11, TRAF3;

Genes included in “Wnt/β-Catenin pathway”: FAT1, TCF3, MAX, DDX41, KDM6A, PIK3CD;

Genes included in “Receptor/Nonreceptor tryosine kinase signaling pathway”: FGFR3, SH2B3, CSF3R, EPHA7;

Genes included in “Cyclins and Cell Cycle Regulation”: CDKN1B, RAD21, MPL, BRINP3, SMC1A.

Supplementary Table 18. Double signaling pathways of 55 children with T-ALL and the comparisons of their survival.

| The main signaling pathways | N (%) | 2-year OS (SE) | *P^*^* value | 2-year EFS (SE) | *P^#^* value | 2-year DFS (SE) | *P^&^* value |
| --- | --- | --- | --- | --- | --- | --- | --- |
| **Total** | 55 (100.0) | 83.4 (5.4) |  | 66.9 (7.7) |  | 64.0 (7.9) |  |
| **Group 1** |  |  | **0.024** |  | 0.305 |  | 0.290 |
| No | 33 (60.0) | 75.7 (7.5) |  | 64.2 (9.3) |  | 60.3 (9.6) |  |
| Yes | 22 (40.0) | 100.0 (0.0) |  | 75.4 (10.9) |  | 75.0 (11.0) |  |
| **Group 2** |  |  | 0.139 |  | **0.010** |  | **0.007** |
| No | 38 (69.1) | 83.7 (6.0) |  | 79.1 (7.2) |  | 79.1 (7.2) |  |
| Yes | 17 (30.9) | 75.1 (10.9) |  | 46.2 (14.2) |  | 37.1 (14.5) |  |
| **Group 4** |  |  | 0.668 |  | 0.648 |  | 0.611 |
| No | 38 (69.1) | 79.6 (6.9) |  | 68.5 (8.0) |  | 64.5 (8.4) |  |
| Yes | 17 (30.9) | 94.1 (5.7) |  | 59.6 (19.0) |  | 59.6 (19.0) |  |
| **Group 3** |  |  | **0.037** |  | **0.032** |  | **0.014** |
| No | 39 (70.9) | 87.8 (5.7) |  | 74.9 (7.3) |  | 74.1 (7.6) |  |
| Yes | 16 (29.1) | 72.2 (12.2) |  | 48.5 (17.1) |  | 38.0 (18.0) |  |
| **Group 10** |  |  | 0.957 |  | 0.060 |  | 0.082 |
| No | 43 (78.2) | 84.1 (6.1) |  | 73.3 (8.3) |  | 69.5 (8.8) |  |
| Yes | 12 (21.8) | 82.5 (11.3) |  | 48.6 (14.8) |  | 48.6 (14.8) |  |
| **Group 18** |  |  | **0.012** |  | **0.010** |  | **0.003** |
| No | 43 (78.2) | 88.7 (5.4) |  | 75.9 (7.2) |  | 74.7 (7.6) |  |
| Yes | 12 (21.8) | 64.8 (14.3) |  | 42.2 (16.5) |  | 31.7 (16.4) |  |
| **Group 25** |  |  | 0.371 |  | 0.880 |  | 0.770 |
| No | 43 (78.2) | 83.8 (6.1) |  | 69.1 (7.6) |  | 65.5 (8.0) |  |
| Yes | 12 (21.8) | 82.5 (11.3) |  | 55.0 (23.7) |  | 55.0 (23.7) |  |
| **Group 12** |  |  | 0.141 |  | 0.392 |  | 0.384 |
| No | 45 (81.8) | 79.6 (6.5) |  | 64.2 (8.8) |  | 60.3 (9.2) |  |
| Yes | 10 (18.2) | 100.0 (0.0) |  | 78.8 (13.4) |  | 78.8 (13.4) |  |
| **Group 19** |  |  | 0.182 |  | 0.343 |  | 0.446 |
| No | 46 (83.6) | 84.2 (6.0) |  | 72.7 (7.3) |  | 68.9 (7.8) |  |
| Yes | 9 (16.4) | 77.8 (13.9) |  | 44.4 (21.0) |  | 44.4 (21.0) |  |
| **Group 11** |  |  | 0.249 |  | 0.954 |  | 0.978 |
| No | 48 (87.3) | 80.8 (6.2) |  | 68.3 (7.8) |  | 65.1 (8.2) |  |
| Yes | 7 (12.7) | 100.0 (0.0) |  | 55.6 (24.8) |  | 55.6 (24.8) |  |
| **Group 6** |  |  | 0.674 |  | 0.274 |  | 0.279 |
| No | 48 (87.3) | 80.7 (6.2) |  | 67.4 (7.3) |  | 64.1 (7.7) |  |
| Yes | 7 (12.7) | 100.0 (0.0) |  | 50.0 (35.4) |  | 50.0 (35.4) |  |
| **Group 14** |  |  | 0.252 |  | 0.416 |  | 0.461 |
| No | 49 (89.1) | 81.2 (6.1) |  | 65.3 (8.2) |  | 62.0 (8.5) |  |
| Yes | 6 (10.9) | 100.0 (0.0) |  | 83.3 (15.2) |  | 83.3 (15.2) |  |
| **Group 7** |  |  | 0.399 |  | 0.963 |  | 0.927 |
| No | 50 (90.9) | 82.0 (5.8) |  | 67.4 (7.8) |  | 64.3 (8.1) |  |
| Yes | 5 (9.1) | 100.0 (0.0) |  | 66.7 (27.2) |  | 66.7 (27.2) |  |
| **Group 13** |  |  | 0.356 |  | 0.182 |  | 0.170 |
| No | 50 (90.9) | 81.8 (5.9) |  | 63.9 (8.1) |  | 60.7 (8.4) |  |
| Yes | 5 (9.1) | 100.0 (0.0) |  | 100.0 (0.0) |  | 100.0 (0.0) |  |
| **Group 20** |  |  | 0.919 |  | 0.952 |  | 0.877 |
| No | 50 (90.9) | 83.8 (5.7) |  | 65.2 (8.3) |  | 61.9 (8.6) |  |
| Yes | 5 (9.1) | 80.0 (17.9) |  | 80.0 (17.9) |  | 80.0 (17.9) |  |
| **Group 21** |  |  | 0.965 |  | 0.606 |  | 0.688 |
| No | 50 (90.9) | 81.6 (5.9) |  | 70.8 (7.0) |  | 67.7 (7.3) |  |
| Yes | 5 (9.1) | 100.0 (0.0) |  | 80.0 (17.9) |  | 80.0 (17.9) |  |
| **Group 9** |  |  | 0.415 |  | 0.262 |  | 0.247 |
| No | 51 (92.7) | 82.1 (5.8) |  | 72.5 (6.6) |  | 61.9 (8.1) |  |
| Yes | 4 (7.3) | 100.0 (0.0) |  | 100.0 (0.0) |  | 100.0 (0.0) |  |
| **Group 5** |  |  | 0.370 |  | 0.610 |  | 0.702 |
| No | 51 (92.7) | 82.2 (5.8) |  | 64.6 (8.0) |  | 61.5 (8.3) |  |
| Yes | 4 (7.3) | 100.0 (0.0) |  | 100.0 (0.0) |  | 100.0 (0.0) |  |
| **Group 15** |  |  | 0.422 |  | 0.975 |  | 0.987 |
| No | 51 (92.7) | 82.1 (5.8) |  | 67.5 (7.8) |  | 64.4 (8.1) |  |
| Yes | 4 (7.3) | 100.0 (0.0) |  | 66.7 (27.2) |  | 66.7 (27.2) |  |
| **Group 17** |  |  | 0.415 |  | 0.242 |  | 0.227 |
| No | 51 (92.7) | 82.1 (5.8) |  | 64.6 (8.0) |  | 61.4 (8.3) |  |
| Yes | 4 (7.3) | 100.0 (0.0) |  | 100.0 (0.0) |  | 100.0 (0.0) |  |
| **Group 31** |  |  | 0.608 |  | 0.850 |  | 0.780 |
| No | 51 (92.7) | 84.1 (5.6) |  | 66.1 (8.1) |  | 62.8 (8.4) |  |
| Yes | 4 (7.3) | 75.0 (21.7) |  | 75.0 (21.7) |  | 75.0 (21.7) |  |
| **Group 32** |  |  | 0.998 |  | 0.535 |  | 0.565 |
| No | 51 (92.7) | 81.8 (5.9) |  | 69.2 (7.0) |  | 66.0 (7.4) |  |
| Yes | 4 (7.3) | 100.0 (0.0) |  | 50.0 (35.4) |  | 50.0 (35.4) |  |
| **Group 33** |  |  | 0.422 |  | 0.993 |  | 0.926 |
| No | 51 (92.7) | 82.1 (5.8) |  | 67.4 (7.8) |  | 64.2 (8.1) |  |
| Yes | 4 (7.3) | 100.0 (0.0) |  | 66.7 (27.2) |  | 66.7 (27.2) |  |
| **Group 8** |  |  | 0.437 |  | 0.299 |  | 0.274 |
| No | 52 (94.5) | 82.5 (5.7) |  | 64.9 (8.2) |  | 61.7 (8.5) |  |
| Yes | 3 (5.5) | 100.0 (0.0) |  | 100.0 (0.0) |  | 100.0 (0.0) |  |
| **Group 16** |  |  | 0.476 |  | 0.947 |  | 0.992 |
| No | 52 (94.5) | 84.7 (5.54) |  | 66.4 (8.2) |  | 63.1 (8.6) |  |
| Yes | 3 (5.5) | 66.7 (27.2) |  | 66.7 (27.2) |  | 66.7 (27.2) |  |
| **Group 26** |  |  | 0.563 |  | 0.472 |  | 0.356 |
| No | 52 (94.5) | 84.5 (5.5) |  | 66.8 (8.0) |  | 63.5 (8.3) |  |
| Yes | 3 (5.5) | 66.7 (27.2) |  | 66.7 (27.2) |  | 66.7 (27.2) |  |
| **Group 27** |  |  | 0.684 |  | 0.968 |  | 0.960 |
| No | 52 (94.5) | 82.2 (5.8) |  | 70.1 (6.9) |  | 67.0 (7.2) |  |
| Yes | 3 (5.5) | 100.0 (0.0) |  | 100.0 (0.0) |  | 100.0 (0.0) |  |
| **Group 28** |  |  | 0.507 |  | 0.390 |  | 0.378 |
| No | 52 (94.5) | 82.5 (5.7) |  | 65.8 (7.8) |  | 62.7 (8.1) |  |
| Yes | 3 (5.5) | 100.0 (0.0) |  | 100.0 (0.0) |  | 100.0 (0.0) |  |
| **Group 34** |  |  | 0.507 |  | 0.334 |  | 0.310 |
| No | 52 (94.5) | 82.5 (5.7) |  | 64.9 (8.2) |  | 61.7 (8.5) |  |
| Yes | 3 (5.5) | 100.0 (0.0) |  | 100.0 (0.0) |  | 100.0 (0.0) |  |
| **Group 35** |  |  | 0.446 |  | 0.294 |  | 0.266 |
| No | 52 (94.5) | 82.2 (5.8) |  | 64.9 (8.0) |  | 61.7 (8.3) |  |
| Yes | 3 (5.5) | 100.0 (0.0) |  | 100.0 (0.0) |  | 100.0 (0.0) |  |

T-ALL, T-cell acute lymphoblastic leukemia; No: single positive or negative; Yes: double positive; ^*^significant differences about 2-year OS; ^#^ significant differences about 2-year EFS; ^&^ significant differences about 2-year DFS; Kaplan-Meier method was used to analyze the survival of each group and the differences between subgroups were evaluated using the log-rank test. Bold values indicate statistical significance at p<0.05.

Group 1: combination of “NOTCH signaling pathway” and “Ras/Protein phosphatase/MARK/PI3K signaling pathway”;

Group 2: combination of “NOTCH signaling pathway” and “Transcription factor/regulation”;

Group 4: combination of “NOTCH signaling pathway” and “Jak-Stat Signaling Pathway”;

Group 3: combination of “NOTCH signaling pathway” and “Epigenetic modulators”;

Group 10: combination of “Ras/Protein phosphatase/MARK/PI3K signaling pathway” and “Transcription factor/regulation”;

Group 18: combination of “Transcription factor/regulation” and “Epigenetic modulators”;

Group 25: combination of “Epigenetic modulators” and “Jak-Stat Signaling Pathway”;

Group 12: combination of “Ras/Protein phosphatase/MARK/PI3K signaling pathway” and “Jak-Stat Signaling Pathway”;

Group 19: combination of “Transcription factor/regulation” and “Jak-Stat Signaling Pathway”;

Group 6: combination of “NOTCH signaling pathway” and “NF-KB pathway”;

Group 14: combination of “Ras/Protein phosphatase/MARK/PI3K signaling pathway” and “NF-KB pathway”;

Group 7: combination of “NOTCH signaling pathway” and “Wnt/β-Catenin pathway”;

Group 13: combination of “Ras/Protein phosphatase/MARK/PI3K signaling pathway” and “Splicing and mRNA processing regulation”;

Group 20: combination of “Transcription factor/regulation” and “Splicing and mRNA processing regulation”;

Group 21: combination of “Transcription factor/regulation” and “NF-KB pathway”;

Group 9: combination of “NOTCH signaling pathway” and “Cyclins and Cell Cycle Regulation”;

Group 5: combination of “NOTCH signaling pathway” and “Splicing and mRNA processing regulation”;

Group 15: combination of “Ras/Protein phosphatase/MARK/PI3K signaling pathway” and “Wnt/β-Catenin pathway”;

Group 17: combination of “Ras/Protein phosphatase/MARK/PI3K signaling pathway” and “Cyclins and Cell Cycle Regulation”;

Group 31: combination of “Jak-Stat Signaling Pathway” and “Splicing and mRNA processing regulation”;

Group 32: combination of “Jak-Stat Signaling Pathway” and “NF-KB pathway”;

Group 33: combination of “Jak-Stat Signaling Pathway” and “Wnt/β-Catenin pathway”;

Group 8: combination of “NOTCH signaling pathway” and “Receptor/Nonreceptor tryosine kinase signaling pathway”;

Group 16: combination of “Ras/Protein phosphatase/MARK/PI3K signaling pathway” and “Receptor/Nonreceptor tryosine kinase signaling pathway”;

Group 26: combination of “Epigenetic modulators” and “Splicing and mRNA processing regulation”;

Group 27: combination of “Epigenetic modulators” and “NF-KB pathway”;

Group 28: combination of “Epigenetic modulators” and “Wnt/β-Catenin pathway”;

Group 34: combination of “Jak-Stat Signaling Pathway” and “Receptor/Nonreceptor tryosine kinase signaling pathway”;

Group 35: combination of “Jak-Stat Signaling Pathway” and “Cyclins and Cell Cycle Regulation”.

**Supplementary Table 19. Univariate analysis of the relationship between double signaling pathways and survival of 55 children with T-ALL**

| Variables | Overall survival (OS) | | | |  | Event-free survival (EFS) | | |  | Relapse-free survival (RFS) | | |
| --- | --- | --- | --- | --- | --- | --- | --- | --- | --- | --- | --- | --- |
|  | OR | 95%CI (OR) | | *p*-value |  | OR | 95%CI (OR) | *p*-value |  | OR | 95%CI (OR) | p-value |
| Group 1 | 0.026 | | 0.000-9.579 | 0.226 |  | 0.656 | 0.212-2.028 | 0.464 |  | 0.650 | 0.210-2.006 | 0.453 |
| Group 2 | 2.597 | | 0.696-9.689 | 0.155 |  | 3.300 | 1.254-8.684 | **0.016** |  | 3.485 | 1.316-9.227 | 0.012 |
| Group 4 | 0.710 | | 0.147-3.424 | 0.670 |  | 0.772 | 0.251-2.378 | 0.652 |  | 0.750 | 0.244-2.307 | 0.615 |
| Group 3 | 3.678 | | 0.985-13.732 | 0.053 |  | 2.699 | 1.038-7.018 | **0.042** |  | 3.129 | 1.195-8.196 | **0.020** |
| Group 10 | 1.045 | | 0.210-5.185 | 0.957 |  | 2.599 | 0.913-7.398 | 0.073 |  | 2.396 | 0.861-6.665 | 0.094 |
| Group 18 | 4.664 | | 1.247-17.435 | **0.022** |  | 3.235 | 1.246-8.397 | **0.016** |  | 3.731 | 1.436-9.690 | **0.007** |
| Group 25 | 1.864 | | 0.464-7.490 | 0.380 |  | 0.909 | 0.259-3.195 | 0.882 |  | 0.832 | 0.239-2.898 | 0.772 |
| Group 12 | 0.036 | | 0.000-45.150 | 0.360 |  | 0.533 | 0.122-2.338 | 0.404 |  | 0.527 | 0.120-2.309 | 0.395 |
| Group 19 | 2.484 | | 0.620-9.949 | 0.199 |  | 1.719 | 0.546-5.414 | 0.355 |  | 1.544 | 0.496-4.808 | 0.453 |
| Group 11 | 0.039 | | 0.000-228.126 | 0.464 |  | 1.044 | 0.234-4.667 | 0.955 |  | 0.980 | 0.221-4.345 | 0.979 |
| Group 6 | 0.642 | | 0.079-5.193 | 0.677 |  | 0.344 | 0.045-2.601 | 0.301 |  | 0.347 | 0.046-2.623 | 0.305 |
| Group 14 | 0.040 | | 0.000-216.621 | 0.464 |  | 0.444 | 0.059-3.373 | 0.433 |  | 0.479 | 0.063-3.612 | 0.475 |
| Group 7 | 0.044 | | 0.000-3191.343 | 0.584 |  | 0.953 | 0.124-7.316 | 0.963 |  | 0.910 | 0.119-6.957 | 0.928 |
| Group 13 | 0.043 | | 0.000-1338.540 | 0.550 |  | 0.042 | 0.000-61.976 | 0.394 |  | 0.042 | 0.000-48.921 | 0.379 |
| Group 20 | 1.113 | | 0.139-8.931 | 0.920 |  | 1.046 | 0.237-4.620 | 0.953 |  | 1.123 | 0.256-4.922 | 0.878 |
| Group 21 | 1.048 | | 0.131-8.399 | 0.965 |  | 1.469 | 0.331-6.519 | 0.613 |  | 1.351 | 0.307-5.950 | 0.691 |
| Group 9 | 0.044 | | 0.000-4487.000 | 0.596 |  | 0.044 | 0.000-217.254 | 0.471 |  | 0.044 | 0.000-160.666 | 0.455 |
| Group 5 | 0.043 | | 0.000-1694.810 | 0.561 |  | 0.595 | 0.078-4.554 | 0.617 |  | 0.677 | 0.090-5.118 | 0.706 |
| Group 15 | 0.044 | | 0.000-5236.807 | 0.601 |  | 1.033 | 0.135-7.915 | 0.975 |  | 0.983 | 0.129-7.501 | 0.987 |
| Group 17 | 0.044 | | 0.000-4487.000 | 0.596 |  | 0.044 | 0.000-149.709 | 0.451 |  | 0.044 | 0.000-112.750 | 0.435 |
| Group 31 | 1.717 | | 0.211-13.967 | 0.613 |  | 0.824 | 0.108-6.279 | 0.852 |  | 0.752 | 0.099-5.703 | 0.782 |
| Group 32 | 0.998 | | 0.120-8.326 | 0.998 |  | 0.534 | 0.070-4.075 | 0.545 |  | 0.558 | 0.074-4.230 | 0.573 |
| Group 33 | 0.044 | | 0.000-5236.807 | 0.601 |  | 0.991 | 0.129-7.581 | 0.993 |  | 0.909 | 0.119-6.918 | 0.926 |
| Group 8 | 0.044 | | 0.000-7610.073 | 0.612 |  | 0.044 | 0.000-386.769 | 0.501 |  | 0.044 | 0.000-244.088 | 0.477 |
| Group 16 | 2.103 | | 0.258-17.137 | 0.487 |  | 1.071 | 0.140-8.208 | 0.947 |  | 0.990 | 0.130-7.563 | 0.993 |
| Group 26 | 1.839 | | 0.225-15.014 | 0.570 |  | 1.721 | 0.381-7.777 | 0.481 |  | 1.979 | 0.448-8.736 | 0.368 |
| Group 27 | 1.536 | | 0.190-12.451 | 0.687 |  | 1.042 | 0.137-7.946 | 0.968 |  | 0.949 | 0.125-7.205 | 0.960 |
| Group 28 | 0.046 | | 0.000-48964.790 | 0.664 |  | 0.046 | 0.000-2219.182 | 0.576 |  | 0.046 | 0.000-1602.266 | 0.564 |
| Group 34 | 0.046 | | 0.000-48964.790 | 0.664 |  | 0.045 | 0.000-724.935 | 0.530 |  | 0.045 | 0.000-451.044 | 0.509 |
| Group 35 | 0.045 | | 0.000-9308.016 | 0.619 |  | 0.045 | 0.000-352.458 | 0.497 |  | 0.044 | 0.000-211.545 | 0.471 |

T-ALL, T-cell acute lymphoblastic leukemia; Cox regression analysis was used to assess the relationship between various gene mutations and survival; Bold values indicate statistical significance at p<0.05.

Group 1: combination of “NOTCH signaling pathway” and “Ras/Protein phosphatase/MARK/PI3K signaling pathway”;

Group 2: combination of “NOTCH signaling pathway” and “Transcription factor/regulation”;

Group 4: combination of “NOTCH signaling pathway” and “Jak-Stat Signaling Pathway”;

Group 3: combination of “NOTCH signaling pathway” and “Epigenetic modulators”;

Group 10: combination of “Ras/Protein phosphatase/MARK/PI3K signaling pathway” and “Transcription factor/regulation”;

Group 18: combination of “Transcription factor/regulation” and “Epigenetic modulators”;

Group 25: combination of “Epigenetic modulators” and “Jak-Stat Signaling Pathway”;

Group 12: combination of “Ras/Protein phosphatase/MARK/PI3K signaling pathway” and “Jak-Stat Signaling Pathway”;

Group 19: combination of “Transcription factor/regulation” and “Jak-Stat Signaling Pathway”;

Group 6: combination of “NOTCH signaling pathway” and “NF-KB pathway”;

Group 14: combination of “Ras/Protein phosphatase/MARK/PI3K signaling pathway” and “NF-KB pathway”;

Group 7: combination of “NOTCH signaling pathway” and “Wnt/β-Catenin pathway”;

Group 13: combination of “Ras/Protein phosphatase/MARK/PI3K signaling pathway” and “Splicing and mRNA processing regulation”;

Group 20: combination of “Transcription factor/regulation” and “Splicing and mRNA processing regulation”;

Group 21: combination of “Transcription factor/regulation” and “NF-KB pathway”;

Group 9: combination of “NOTCH signaling pathway” and “Cyclins and Cell Cycle Regulation”;

Group 5: combination of “NOTCH signaling pathway” and “Splicing and mRNA processing regulation”;

Group 15: combination of “Ras/Protein phosphatase/MARK/PI3K signaling pathway” and “Wnt/β-Catenin pathway”;

Group 17: combination of “Ras/Protein phosphatase/MARK/PI3K signaling pathway” and “Cyclins and Cell Cycle Regulation”;

Group 31: combination of “Jak-Stat Signaling Pathway” and “Splicing and mRNA processing regulation”;

Group 32: combination of “Jak-Stat Signaling Pathway” and “NF-KB pathway”;

Group 33: combination of “Jak-Stat Signaling Pathway” and “Wnt/β-Catenin pathway”;

Group 8: combination of “NOTCH signaling pathway” and “Receptor/Nonreceptor tryosine kinase signaling pathway”;

Group 16: combination of “Ras/Protein phosphatase/MARK/PI3K signaling pathway” and “Receptor/Nonreceptor tryosine kinase signaling pathway”;

Group 26: combination of “Epigenetic modulators” and “Splicing and mRNA processing regulation”;

Group 27: combination of “Epigenetic modulators” and “NF-KB pathway”;

Group 28: combination of “Epigenetic modulators” and “Wnt/β-Catenin pathway”;

Group 34: combination of “Jak-Stat Signaling Pathway” and “Receptor/Nonreceptor tryosine kinase signaling pathway”;

Group 35: combination of “Jak-Stat Signaling Pathway” and “Cyclins and Cell Cycle Regulation”.

**Supplementary Table 20. Multivariate analysis of the relationship between double signaling pathways and survival of 55 children with T-ALL**

| Variables | Overall survival (OS) | | | |  | Event-free survival (EFS) | | |  | Relapse-free survival (RFS) | | |
| --- | --- | --- | --- | --- | --- | --- | --- | --- | --- | --- | --- | --- |
|  | OR | 95%CI (OR) | | *p*-value |  | OR | 95%CI (OR) | *p*-value |  | OR | 95%CI (OR) | p-value |
| Group 1 | 0.000 | | 0.000-3.359E+232 | 0.965 |  | 0.935 | 0.296-2.957 | 0.909 |  | 0.907 | 0.288-2.863 | 0.868 |
| MRD at day 19 | 3.023 | | 1.406-6.497 | **0.005** |  | 2.558 | 1.572-4.162 | **0.000** |  | 2.326 | 1.487-3.640 | **0.000** |
| Group 1 | 0.000 | | 0.000-1.414E+158 | 0.949 |  | 0.730 | 0.234-2.283 | 0.589 |  | 0.704 | 0.225-2.204 | 0.547 |
| MRD at day 46 | 3.084 | | 1.757-5.412 | **0.000** |  | 2.255 | 1.584-3.209 | **0.000** |  | 2.293 | 1.604-3.278 | **0.000** |
| Group 2 | 1.409 | | 0.357-5.552 | 0.625 |  | 2.410 | 0.885-6.567 | 0.085 |  | 2.443 | 0.886-6.731 | 0.084 |
| MRD at day 19 | 3.236 | | 1.432-7.314 | **0.005** |  | 2.404 | 1.482-3.900 | **0.000** |  | 2.166 | 1.389-3.379 | **0.001** |
| Group 2 | 1.693 | | 0.429-6.685 | 0.452 |  | 2.732 | 1.020-7.313 | **0.045** |  | 3.007 | 1.111-8.137 | **0.030** |
| MRD at day 46 | 2.709 | | 1.675-4.383 | **0.000** |  | 2.189 | 1.521-3.151 | **0.000** |  | 2.232 | 1.548-3.217 | **0.000** |
| Group 4 | 0.458 | | 0.085-2.476 | 0.346 |  | 0.630 | 0.197-2.012 | 0.436 |  | 0.627 | 0.197-1.993 | 0.429 |
| MRD at day 19 | 3.664 | | 1.573-8.532 | **0.003** |  | 2.649 | 1.617-4.340 | **0.000** |  | 2.412 | 1.526-3.812 | **0.001** |
| Group 4 | 0.354 | | 0.066-1.889 | 0.224 |  | 0.526 | 0.166-1.669 | 0.276 |  | 0.497 | 0.156-1.590 | 0.239 |
| MRD at day 46 | 3.073 | | 1.841-5.130 | **0.000** |  | 2.348 | 1.650-3.339 | **0.000** |  | 2.406 | 1.675-3.455 | **0.000** |
| Group 3 | 1.935 | | 0.458-7.728 | 0.350 |  | 1.798 | 0.668-4.839 | 0.246 |  | 2.027 | 0.745-5.517 | 0.167 |
| MRD at day 19 | 3.068 | | 1.361-6.914 | **0.007** |  | 2.429 | 1.495-3.947 | **0.000** |  | 2.171 | 1.391-3.390 | **0.000** |
| Group 3 | 4.274 | | 1.031-17.716 | **0.045** |  | 3.164 | 1.168-8.571 | **0.024** |  | 3.777 | 1.370-10.409 | **0.010** |
| MRD at day 46 | 3.054 | | 1.783-5.230 | **0.000** |  | 2.416 | 1.654-3.528 | **0.000** |  | 2.448 | 1.686-3.671 | **0.000** |
| Group 10 | 0.587 | | 0.115-2.985 | 0.520 |  | 0.451 | 0.689-5.787 | 0.202 |  | 1.892 | 0.668-5.362 | 0.230 |
| MRD at day 19 | 3.416 | | 1.559-7.483 | **0.002** |  | 2.582 | 1.566-4.257 | **0.000** |  | 2.236 | 1.482-3.681 | **0.000** |
| Group 10 | 0.730 | | 0.134-3.986 | 0.716 |  | 2.287 | 0.792-6.607 | 0.126 |  | 2.056 | 0.723-5.844 | 0.176 |
| MRD at day 46 | 2.854 | | 1.749-4.657 | **0.000** |  | 2.219 | 1.561-3.155 | **0.000** |  | 2.240 | 1.572-3.193 | **0.000** |
| Group 18 | 1.455 | | 0.334-6.334 | 0.618 |  | 1.342 | 0.469-3.837 | 0.584 |  | 1.579 | 0.542-4.603 | 0.402 |
| MRD at day 19 | 3.075 | | 1.314-7.196 | **0.010** |  | 2.433 | 1.458-4.061 | **0.000** |  | 2.153 | 1.337-3.466 | **0.002** |
| Group 18 | 2.698 | | 0.648-11.240 | 0.173 |  | 2.148 | 0.776-5.942 | 0.141 |  | 2.719 | 0.991-7.462 | 0.052 |
| MRD at day 46 | 2.599 | | 1.570-4.303 | **0.000** |  | 2.087 | 1.445-3.013 | **0.000** |  | 2.122 | 1.469-3.064 | **0.000** |
| Group 25 | 0.776 | | 0.148-4.073 | 0.765 |  | 0.393 | 0.097-1.590 | 0.190 |  | 0.406 | 0.104-1.584 | 0.194 |
| MRD at day 19 | 3.535 | | 1.464-8.534 | **0.005** |  | 2.972 | 1.679-5.261 | **0.000** |  | 2.634 | 1.587-4.371 | **0.000** |
| Group 25 | 1.058 | | 0.219-5.107 | 0.944 |  | 0.532 | 0.136-2.290 | 0.366 |  | 0.578 | 0.156-2.146 | 0.413 |
| MRD at day 46 | 2.788 | | 1.704-4.563 | **0.000** |  | 2.291 | 1.643-3.537 | **0.000** |  | 2.391 | 1.648-3.467 | **0.000** |
| Group 12 | 0.000 | | 0.000-. | 0.982 |  | 1.008 | 0.222-4.581 | 0.992 |  | 1.024 | 0.225-4.663 | 0.975 |
| MRD at day 19 | 3.208 | | 1.424-7.224 | **0.005** |  | 2.572 | 1.580-4.188 | **0.000** |  | 2.345 | 1.493-3.685 | **0.000** |
| Group 12 | 0.000 | | 0.000-. | 0.979 |  | 0.452 | 0.101-2.016 | 0.298 |  | 0.434 | 0.097-1.946 | 0.275 |
| MRD at day 46 | 3.277 | | 1.864-5.760 | **0.000** |  | 2.315 | 1.621-3.305 | **0.000** |  | 2.365 | 1.644-3.401 | **0.000** |
| Group 19 | 0.741 | | 0.150-3.656 | 0.712 |  | 0.667 | 0.191-2.325 | 0.525 |  | 0.687 | 0.202 -2.335 | 0.547 |
| MRD at day 19 | 3.635 | | 1.434-9.213 | **0.007** |  | 2.745 | 1.608-4.686 | **0.000** |  | 2.469 | 1.521-4.007 | **0.000** |
| Group 19 | 0.413 | | 0.056-3.040 | 0.385 |  | 0.479 | 0.104-2.215 | 0.346 |  | 0.556 | 0.138-2.240 | 0.409 |
| MRD at day 46 | 3.423 | | 1.744-6.717 | **0.000** |  | 2.623 | 1.632-4.217 | **0.000** |  | 2.551 | 1.645-3.955 | **0.000** |
| Group 11 | 0.000 | | 0.000- | 0.985 |  | 1.013 | 0.225-4.560 | 0.987 |  | 0.925 | 0.208-4.113 | 0.918 |
| MRD at day 19 | 3.234 | | 1.502-6.963 | **0.000** |  | 2.572 | 1.589-4.165 | **0.000** |  | 2.339 | 1.504-3.638 | **0.000** |
| Group 11 | 0.000 | | 0.000- | 0.987 |  | 1.862 | 0.382-9.067 | 0.442 |  | 1.669 | 0.350-7.949 | 0.520 |
| MRD at day 46 | 2.668 | | 1.667-4.268 | **0.000** |  | 2.350 | 1.623-3.404 | **0.000** |  | 2.366 | 1.633-3.430 | **0.000** |
| Group 6 | 0.533 | | 0.044-6.425 | 0.621 |  | 0.412 | 0.054-3.176 | 0.395 |  | 0.415 | 0.054-3.176 | 0.397 |
| MRD at day 19 | 3.455 | | 1.520-7.853 | **0.003** |  | 2.560 | 1.579-4.150 | **0.000** |  | 2.334 | 1.493-3.649 | **0.000** |
| Group 6 | 1.575 | | 0.162-15.296 | 0.695 |  | 0.561 | 0.071-4.440 | 0.584 |  | 0.585 | 0.074-4.615 | 0.611 |
| MRD at day 46 | 2.880 | | 1.748-4.747 | **0.000** |  | 2.214 | 1.548-3.167 | **0.000** |  | 2.246 | 1.563-3.227 | **0.000** |
| Group 14 | 0.000 | | 0.000- | 0.984 |  | 0.813 | 0.101-6.508 | 0.845 |  | 0.969 | 0.122-7.699 | 0.976 |
| MRD at day 19 | 3.294 | | 1.476-7.354 | **0.004** |  | 2.550 | 1.569-4.144 | **0.000** |  | 2.339 | 1.489-3.674 | **0.000** |
| Group 14 | 0.000 | | 0.000- | 0.987 |  | 0.727 | 0.091-5.804 | 0.763 |  | 0.827 | 0.105-6.538 | 0.857 |
| MRD at day 46 | 2.679 | | 1.667-4.306 | **0.000** |  | 2.241 | 1.567-3.204 | **0.000** |  | 2.282 | 1.588-3.279 | **0.000** |
| Group 7 | 0.000 | | 0.000- | 0.987 |  | 0.805 | 0.104-6.234 | 0.836 |  | 0.786 | 0.102-6.076 | 0.818 |
| MRD at day 19 | 3.390 | | 1.534-7.491 | **0.003** |  | 2.575 | 1.593-4.163 | **0.000** |  | 2.347 | 1.507-3.654 | **0.000** |
| Group 7 | 0.000 | | 0.000- | 0.987 |  | 0.214 | 0.029-2.000 | 0.188 |  | 0.197 | 0.023-1.669 | 0.136 |
| MRD at day 46 | 3.856 | | 2.037-7.300 | **0.000** |  | 2.507 | 1.739-3.614 | **0.000** |  | 2.646 | 1.800-3.890 | **0.000** |
| Group 13 | 0.000 | | 0.000- | 0.992 |  | 0.000 | 0.000- | 0.986 |  | 0.000 | 0.000- | 0.985 |
| MRD at day 19 | 3.241 | | 1.442-7.283 | **0.004** |  | 2.441 | 1.506-3.957 | **0.000** |  | 2.218 | 1.424-3.456 | **0.000** |
| Group 13 | 0.000 | | 0.000- | 0.990 |  | 0.000 | 0.000- | 0.981 |  | 0.000 | 0.000- | 0.987 |
| MRD at day 46 | 2.714 | | 1.688-4.363 | **0.000** |  | 2.182 | 1.533-3.107 | **0.000** |  | 2.212 | 1.548-3.159 | **0.000** |
| Group 20 | 1.418 | | 0.151-13.316 | 0.760 |  | 1.221 | 0.268-5.568 | 0.797 |  | 1.548 | 0.345-6.948 | 0.569 |
| MRD at day 19 | 3.293 | | 1.504-7.209 | **0.003** |  | 2.588 | 1.591-4.114 | **0.000** |  | 2.343 | 1.520-3.612 | **0.000** |
| Group 20 | 0.468 | | 0.046-4.725 | 0.519 |  | 0.531 | 0.105-2.699 | 0.446 |  | 0.638 | 0.128-3.188 | 0.584 |
| MRD at day 46 | 2.963 | | 1.788-4.909 | **0.000** |  | 2.405 | 1.632-3.543 | **0.000** |  | 2.383 | 1.629-3.485 | **0.000** |
| Group 21 | 0.351 | | 0.033-3.701 | 0.384 |  | 0.963 | 0.214-4.342 | 0.961 |  | 0.907 | 0.202-4.059 | 0.898 |
| MRD at day 19 | 3.668 | | 1.596-8.430 | **0.002** |  | 2.574 | 1.590-4.167 | **0.000** |  | 2.349 | 1.506-3.665 | **0.000** |
| Group 21 | 2.690 | | 0.281-25.729 | 0.390 |  | 2.938 | 0.600-14.389 | 0.184 |  | 2.543 | 0.531-12.175 | 0.243 |
| MRD at day 46 | 3.826 | | 1.789-4.878 | **0.000** |  | 2.399 | 1.658-3.471 | **0.000** |  | 2.410 | 1.664-3.490 | **0.000** |
| Group 9 | 0.000 | | 0.000- | 0.991 |  | 0.000 | 0.000- | 0.987 |  | 0.000 | 0.000- | 0.986 |
| MRD at day 19 | 3.262 | | 1.462-7.275 | **0.004** |  | 2.483 | 1.583-4.010 | **0.000** |  | 2.260 | 1.454-3.515 | **0.000** |
| Group 9 | 0.000 | | 0.000- | 0.991 |  | 0.000 | 0.000- | 0.984 |  | 0.000 | 0.000- | 0.984 |
| MRD at day 46 | 2.732 | | 1.700-4.391 | **0.000** |  | 2.202 | 1.548-3.134 | **0.000** |  | 2.232 | 1.563-3.187 | **0.000** |
| Group 5 | 0.000 | | 0.000- | 0.992 |  | 2.179 | 0.210-22.579 | 0.514 |  | 3.184 | 0.312-32.453 | 0.328 |
| MRD at day 19 | 3.266 | | 1.449-7.365 | **0.004** |  | 2.680 | 1.619-4.437 | **0.000** |  | 2.530 | 1.545-4.144 | **0.000** |
| Group 5 | 0.000 | | 0.000- | 0.989 |  | 0.933 | 0.115-7.537 | 0.948 |  | 1.142 | 0.144-9.035 | 0.900 |
| MRD at day 46 | 2.729 | | 1.698-4.383 | **0.000** |  | 2.262 | 1.585-3.228 | **0.000** |  | 2.308 | 1.609-3.312 | **0.000** |
| Group 15 | 0.000 | | 0.000- | 0.988 |  | 0.951 | 0.123-7.379 | 0.962 |  | 0.925 | 0.120-7.152 | 0.941 |
| MRD at day 19 | 3.394 | | 1.526-7.548 | **0.003** |  | 2.572 | 1.591-4.158 | **0.000** |  | 2.343 | 1.505-3.648 | **0.000** |
| Group 15 | 0.000 | | 0.000- | 0.991 |  | 0.383 | 0.047 -3.156 | 0.373 |  | 0.345 | 0.041-2.874 | 0.325 |
| MRD at day 46 | 3.458 | | 1.959-6.106 | **0.000** |  | 2.393 | 1.661-3.447 | **0.000** |  | 2.462 | 1.692-3.582 | **0.000** |
| Group 17 | 0.000 | | 0.000- | 0.992 |  | 0.000 | 0.000- | 0.987 |  | 0.000 | 0.000- | 0.986 |
| MRD at day 19 | 3.234 | | 1.456-7.184 | **0.004** |  | 2.460 | 1.531-3.955 | **0.000** |  | 2.244 | 1.450-3.472 | **0.000** |
| Group 17 | 0.000 | | 0.000- | 0.991 |  | 0.000 | 0.000- | 0.983 |  | 0.000 | 0.000- | 0.988 |
| MRD at day 46 | 2.732 | | 1.700-4.391 | **0.000** |  | 2.201 | 1.547-3.131 | **0.000** |  | 2.230 | 1.562-3.184 | **0.000** |
| Group 31 | 1.515 | | 0.154-14.919 | 0.722 |  | 0.742 | 0.093-5.935 | 0.778 |  | 0.824 | 0.106 -6.430 | 0.854 |
| MRD at day 19 | 3.257 | | 1.480-7.170 | **0.003** |  | 2.599 | 1.585-4.261 | **0.000** |  | 2.349 | 1.502-3.672 | **0.000** |
| Group 31 | 0.529 | | 0.049-5.764 | 0.601 |  | 0.262 | 0.028-2.428 | 0.238 |  | 0.298 | 0.033-2.670 | 0.279 |
| MRD at day 46 | 2.950 | | 1.770-4.916 | **0.000** |  | 2.552 | 1.708-3.813 | **0.000** |  | 2.520 | 1.703-3.730 | **0.000** |
| Group 32 | 0.591 | | 0.045-7.726 | 0.688 |  | 0.502 | 0.064-3.930 | 0.511 |  | 0.525 | 0.068-4.083 | 0.538 |
| MRD at day 19 | 3.476 | | 1.522-7.938 | **0.003** |  | 2.596 | 1.600-4.213 | **0.000** |  | 2.365 | 1.512-3.699 | **0.000** |
| Group 32 | 2.299 | | 0.227-23.274 | 0.481 |  | 0.817 | 0.102-6.554 | 0.849 |  | 0.898 | 0.114-7.103 | 0.919 |
| MRD at day 46 | 2.913 | | 1.776-4.777 | **0.000** |  | 2.253 | 1.579-3.215 | **0.000** |  | 2.291 | 1.598-3.284 | **0.000** |
| Group 33 | 0.000 | | 0.000-. | 0.992 |  | 0.849 | 0.110-6.568 | 0.875 |  | 0.824 | 0.107-6.342 | 0.852 |
| MRD at day 19 | 3.414 | | 1.540-7.568 | **0.002** |  | 2.575 | 1.593-4.164 | **0.000** |  | 2.346 | 1.506-3.654 | **0.000** |
| Group 33 | 0.000 | | 0.000-. | 0.987 |  | 0.240 | 0.029-1.995 | 0.187 |  | 0.194 | 0.023-1.642 | 0.132 |
| MRD at day 46 | 3.864 | | 2.043-7.310 | **0.000** |  | 2.511 | 1.742-3.620 | **0.000** |  | 2.652 | 1.804-3.898 | **0.000** |
| Group 8 | 0.000 | | 0.000- | 0.991 |  | 0.000 | 0.000- | 0.987 |  | 0.000 | 0.000- | 0.986 |
| MRD at day 19 | 3.295 | | 1.476-7.357 | **0.004** |  | 2.506 | 1.550-4.052 | **0.000** |  | 2.277 | 1.463-3.546 | **0.000** |
| Group 8 | 0.000 | | 0.000- | 0.990 |  | 0.000 | 0.000- | 0.984 |  | 0.000 | 0.000- | 0.983 |
| MRD at day 46 | 2.749 | | 1.712-4.413 | **0.000** |  | 2.224 | 1.564-3.162 | **0.000** |  | 2.252 | 1.578-3.214 | **0.000** |
| Group 16 | 2.031 | | 0.242-17.045 | 0.514 |  | 1.578 | 0.168-12.408 | 0.665 |  | 1.498 | 0.191-11.759 | 0.700 |
| MRD at day 19 | 3.390 | | 1.512-7.599 | **0.003** |  | 2.604 | 1.599-4.240 | **0.000** |  | 2.369 | 1.511-3.713 | **0.000** |
| Group 16 | 6.570 | | 0.628-68.741 | 0.116 |  | 1.877 | 0.230-15.352 | 0.557 |  | 1.661 | 0.206-13.418 | 0.634 |
| MRD at day 46 | 3.078 | | 1.833-5.169 | **0.000** |  | 2.304 | 1.611-3.295 | **0.000** |  | 2.329 | 1.624-3.339 | **0.000** |
| Group 26 | 1.628 | | 0.160-16.544 | 0.681 |  | 1.502 | 0.314-7.198 | 0.611 |  | 2.153 | 0.460-10.069 | 0.330 |
| MRD at day 19 | 3.224 | | 1.467-7.086 | **0.004** |  | 2.516 | 1.566-4.042 | **0.000** |  | 2.292 | 1.498-3.507 | **0.000** |
| Group 26 | 0.511 | | 0.048-5.484 | 0.579 |  | 0.626 | 0.115-3.415 | 0.589 |  | 0.799 | 0.147-4.330 | 0.794 |
| MRD at day 46 | 2.965 | | 1.777-4.947 | **0.000** |  | 2.384 | 1.603-3.546 | **0.000** |  | 2.349 | 1.590-3.470 | **0.000** |
| Group 27 | 0.618 | | 0.047-8.084 | 0.714 |  | 0.669 | 0.084-5.345 | 0.705 |  | 0.607 | 0.077 -4.792 | 0.635 |
| MRD at day 19 | 3.488 | | 1.523-7.990 | **0.003** |  | 2.601 | 1.601-4.226 | **0.000** |  | 2.379 | 1.518-3.727 | **0.000** |
| Group 27 | 3.690 | | 0.381-35.727 | 0.260 |  | 1.909 | 0.236-15.441 | 0.544 |  | 1.657 | 0.208-13.229 | 0.634 |
| MRD at day 46 | 2.964 | | 1.802-4.876 | **0.000** |  | 2.310 | 1.612-3.309 | **0.000** |  | 2.332 | 1.624-3.349 | **0.000** |
| Group 28 | 0.000 | | 0.000- | 0.991 |  | 0.000 | 0.000- | 0.989 |  | 0.000 | 0.000- | 0.989 |
| MRD at day 19 | 3.322 | | 1.491-7.402 | **0.003** |  | 2.533 | 1.565-4.100 | **0.000** |  | 2.305 | 1.479-3.592 | **0.000** |
| Group 28 | 0.000 | | 0.000- | 0.989 |  | 0.000 | 0.000- | 0.988 |  | 0.000 | 0.000- | 0.987 |
| MRD at day 46 | 2.755 | | 1.716-4.426 | **0.000** |  | 2.228 | 1.567-3.169 | **0.000** |  | 2.259 | 1.583-3.224 | **0.000** |
| Group 34 | 0.000 | | 0.000-. | 0.992 |  | 0.000 | 0.000- | 0.989 |  | 0.000 | 0.000- | 0.988 |
| MRD at day 19 | 3.295 | | 1.485-7.312 | **0.003** |  | 2.504 | 1.553-4.035 | **0.000** |  | 2.279 | 1.469-3.536 | **0.000** |
| Group 34 | 0.000 | | 0.000-. | 0.989 |  | 0.000 | 0.000- | 0.985 |  | 0.000 | 0.000- | 0.985 |
| MRD at day 46 | 2.755 | | 1.716-4.426 | **0.000** |  | 2.226 | 1.565-3.165 | **0.000** |  | 2.255 | 1.580-3.218 | **0.000** |
| Group 35 | 0.000 | | 0.000- | 0.993 |  | 0.000 | 0.000- | 0.989 |  | 0.000 | 0.000- | 0.988 |
| MRD at day 19 | 3.271 | | 1.462-7.322 | **0.004** |  | 2.483 | 1.537-4.013 | **0.000** |  | 2.256 | 1.451-3.506 | **0.000** |
| Group 35 | 0.000 | | 0.000-. | 0.992 |  | 0.000 | 0.000-. | 0.985 |  | 0.000 | 0.000-. | 0.983 |
| MRD at day 46 | 2.741 | | 1.707-4.401 | **0.000** |  | 2.215 | 1.557-3.150 | **0.000** |  | 2.243 | 1.572-3.201 | **0.000** |

T-ALL, T-cell acute lymphoblastic leukemia; MRD, minimal residual disease; Cox regression analysis was used to assess the relationship between various gene mutations and survival; Bold values indicate statistical significance at p<0.05.

Group 1: combination of “NOTCH signaling pathway” and “Ras/Protein phosphatase/MARK/PI3K signaling pathway”;

Group 2: combination of “NOTCH signaling pathway” and “Transcription factor/regulation”;

Group 4: combination of “NOTCH signaling pathway” and “Jak-Stat Signaling Pathway”;

Group 3: combination of “NOTCH signaling pathway” and “Epigenetic modulators”;

Group 10: combination of “Ras/Protein phosphatase/MARK/PI3K signaling pathway” and “Transcription factor/regulation”;

Group 18: combination of “Transcription factor/regulation” and “Epigenetic modulators”;

Group 25: combination of “Epigenetic modulators” and “Jak-Stat Signaling Pathway”;

Group 12: combination of “Ras/Protein phosphatase/MARK/PI3K signaling pathway” and “Jak-Stat Signaling Pathway”;

Group 19: combination of “Transcription factor/regulation” and “Jak-Stat Signaling Pathway”;

Group 6: combination of “NOTCH signaling pathway” and “NF-KB pathway”;

Group 14: combination of “Ras/Protein phosphatase/MARK/PI3K signaling pathway” and “NF-KB pathway”;

Group 7: combination of “NOTCH signaling pathway” and “Wnt/β-Catenin pathway”;

Group 13: combination of “Ras/Protein phosphatase/MARK/PI3K signaling pathway” and “Splicing and mRNA processing regulation”;

Group 20: combination of “Transcription factor/regulation” and “Splicing and mRNA processing regulation”;

Group 21: combination of “Transcription factor/regulation” and “NF-KB pathway”;

Group 9: combination of “NOTCH signaling pathway” and “Cyclins and Cell Cycle Regulation”;

Group 5: combination of “NOTCH signaling pathway” and “Splicing and mRNA processing regulation”;

Group 15: combination of “Ras/Protein phosphatase/MARK/PI3K signaling pathway” and “Wnt/β-Catenin pathway”;

Group 17: combination of “Ras/Protein phosphatase/MARK/PI3K signaling pathway” and “Cyclins and Cell Cycle Regulation”;

Group 31: combination of “Jak-Stat Signaling Pathway” and “Splicing and mRNA processing regulation”;

Group 32: combination of “Jak-Stat Signaling Pathway” and “NF-KB pathway”;

Group 33: combination of “Jak-Stat Signaling Pathway” and “Wnt/β-Catenin pathway”;

Group 8: combination of “NOTCH signaling pathway” and “Receptor/Nonreceptor tryosine kinase signaling pathway”;

Group 16: combination of “Ras/Protein phosphatase/MARK/PI3K signaling pathway” and “Receptor/Nonreceptor tryosine kinase signaling pathway”;

Group 26: combination of “Epigenetic modulators” and “Splicing and mRNA processing regulation”;

Group 27: combination of “Epigenetic modulators” and “NF-KB pathway”;

Group 28: combination of “Epigenetic modulators” and “Wnt/β-Catenin pathway”;

Group 34: combination of “Jak-Stat Signaling Pathway” and “Receptor/Nonreceptor tryosine kinase signaling pathway”;

Group 35: combination of “Jak-Stat Signaling Pathway” and “Cyclins and Cell Cycle Regulation”.

Supplementary Table 21. "NOTCH signaling pathway", interaction with specific genes in "Transcription factor/regulation" for survival

| The main signaling pathways | N (%) | 2-year OS (SE) | *P^*^* value | 2-year EFS (SE) | *P^#^* value | 2-year DFS (SE) | *P^&^* value |
| --- | --- | --- | --- | --- | --- | --- | --- |
| **Total** | 55 (100.0) | 83.4 (5.4) |  | 66.9 (7.7) |  | 64.0 (7.9) |  |
| **NOTCH signaling pathway and CREBBP** |  |  | **0.016** |  | **0.008** |  | **0.001** |
| No | 50 (90.9) | 88.8 (4.8) |  | 69.5 (8.3) |  | 69.5 (8.3) |  |
| Yes | 5 (9.1) | 40.0 (21.9) |  | 40.0 (21.9) |  | 20.0 (17.9) |  |
| **NOTCH signaling pathway and PHF6** |  |  | 0.817 |  | 0.971 |  | 0.892 |
| No | 48 (87.3) | 81.0 (6.1) |  | 69.9 (7.1) |  | 66.8 (7.5) |  |
| Yes | 7 (12.7) | 100.0 (0.0) |  | 42.9 (31.0) |  | 42.9 (31.0) |  |
| **NOTCH signaling pathway and WT1** |  |  | 0.601 |  | 0.881 |  | 0.862 |
| No | 48 (87.3) | 83.4 (5.8) |  | 69.9 (7.2) |  | 66.4 (7.6) |  |
| Yes | 7 (12.7) | 83.3 (15.2) |  | 62.5 (21.3) |  | 62.5 (21.3) |  |

T-ALL, T-cell acute lymphoblastic leukemia; No: single positive or negative; Yes: double positive; ^*^significant differences about 2-year OS; ^#^ significant differences about 2-year EFS; ^&^ significant differences about 2-year DFS; Kaplan-Meier method was used to analyze the survival of each group and the differences between subgroups were evaluated using the log-rank test. Bold values indicate statistical significance at p<0.05.

Genes included in “NOTCH signaling pathway”: NOTCH2, NOTCH1, FBXW7;

Genes included in “Transcription factor/regulation”: CREBBP, RUNX1, CEBPA, PHF6, WT1, PRDM1, GATA2, GATA3, SETBP1, BCORL1, TERT, KMT2A; Among them, the number of positive cases of RUNX1, CEBPA, PRDM1, GATA2, GATA3, SETBP1, BCORL1, TERT, and KMT2A were all less than 3, which were not suitable for analysis.

**Supplementary Table 22.** **"NOTCH signaling pathway", interaction with specific genes in "Transcription factor/regulation" for survival (Univariate analysis)**

| Variables | Overall survival (OS) | | | |  | Event-free survival (EFS) | | |  | Relapse-free survival (RFS) | | |
| --- | --- | --- | --- | --- | --- | --- | --- | --- | --- | --- | --- | --- |
|  | OR | 95%CI (OR) | | *p*-value |  | OR | 95%CI (OR) | *p*-value |  | OR | 95%CI (OR) | p-value |
| NOTCH signaling pathway and CREBBP | 4.730 | | 1.176-19.024 | **0.029** |  | 3.826 | 1.311-11.162 | **0.014** |  | 4.772 | 1.669-13.639 | **0.004** |
| NOTCH signaling pathway and PHF6 | 0.783 | | 0.098-6.268 | 0.818 |  | 0.972 | 0.219-4.318 | 0.840 |  | 0.903 | 0.205-3.977 | 0.893 |
| NOTCH signaling pathway and WT1 | 1.520 | | 0.311-7.429 | 0.605 |  | 1.100 | 0.314-3.856 | 0.882 |  | 1.116 | 0.319-3.911 | 0.863 |

T-ALL, T-cell acute lymphoblastic leukemia; Cox regression analysis was used to assess the relationship between various gene mutations and survival; Bold values indicate statistical significance at p<0.05.

Genes included in “NOTCH signaling pathway”: NOTCH2, NOTCH1, FBXW7;

Genes included in “Transcription factor/regulation”: CREBBP, RUNX1, CEBPA, PHF6, WT1, PRDM1, GATA2, GATA3, SETBP1, BCORL1, TERT, KMT2A; Among them, the number of positive cases of RUNX1, CEBPA, PRDM1, GATA2, GATA3, SETBP1, BCORL1, TERT, and KMT2A were all less than 3, which were not suitable for analysis.

**Supplementary Table 23. "NOTCH signaling pathway", interaction with specific genes in "Transcription factor/regulation" for survival (Multivariate analysis)**

| Variables | Overall survival (OS) | | | |  | Event-free survival (EFS) | | |  | Relapse-free survival (RFS) | | |
| --- | --- | --- | --- | --- | --- | --- | --- | --- | --- | --- | --- | --- |
|  | OR | 95%CI (OR) | | *p*-value |  | OR | 95%CI (OR) | *p*-value |  | OR | 95%CI (OR) | p-value |
| NOTCH signaling pathway and CREBBP | 4.593 | | 1.074-19.632 | **0.040** |  | 3.816 | 1.296-11.229 | **0.015** |  | 4.623 | 1.577-13.553 | **0.005** |
| MRD at day 19 | 3.326 | | 1.522-7.270 | **0.003** |  | 2.573 | 1.590-4.162 | **0.000** |  | 2.373 | 1.502-3.749 | **0.000** |
| NOTCH signaling pathway and CREBBP | 3.323 | | 0.776-14.224 | 0.105 |  | 2.984 | 1.018-8.742 | **0.046** |  | 4.249 | 1.459-12.379 | **0.008** |
| MRD at day 46 | 2.757 | | 1.675-4.539 | **0.000** |  | 2.226 | 1.543-3.212 | **0.000** |  | 2.315 | 1.589-3.371 | **0.000** |
| NOTCH signaling pathway and PHF6 | 0.349 | | 0.033-3.634 | 0.378 |  | 0.564 | 0.117-2.720 | 0.476 |  | 0.509 | 0.107-2.412 | 0.395 |
| MRD at day 19 | 3.647 | | 1.584-8.393 | **0.002** |  | 2.656 | 1.624-4.343 | **0.000** |  | 2.447 | 1.546-3.873 | **0.000** |
| NOTCH signaling pathway and PHF6 | 2.103 | | 0.220-20.150 | 0.519 |  | 1.930 | 0.395-9.444 | 0.417 |  | 1.704 | 0.356-8.155 | 0.505 |
| MRD at day 46 | 2.934 | | 1.771-4.863 | **0.000** |  | 2.359 | 1.629-3.416 | **0.000** |  | 2.373 | 1.637-3.440 | **0.000** |
| NOTCH signaling pathway and WT1 | 0.706 | | 0.125-3.987 | 0.694 |  | 0.601 | 0.161-2.245 | 0.449 |  | 0.621 | 0.167-2.310 | 0.477 |
| MRD at day 19 | 3.471 | | 1.538-7.835 | **0.003** |  | 2.669 | 1.633-4.362 | **0.000** |  | 2.430 | 1.539-3.837 | **0.000** |
| NOTCH signaling pathway and WT1 | 2.213 | | 0.419-11.679 | 0.349 |  | 1.289 | 0.360-4.611 | 0.696 |  | 1.368 | 0.382-4.902 | 0.630 |
| MRD at day 46 | 2.919 | | 1.779-4.788 | **0.000** |  | 2.385 | 1.601-3.262 | **0.000** |  | 2.326 | 1.621-3.338 | **0.000** |

T-ALL, T-cell acute lymphoblastic leukemia; MRD, minimal residual disease; Cox regression analysis was used to assess the relationship between various gene mutations and survival; Bold values indicate statistical significance at p<0.05.

Genes included in “NOTCH signaling pathway”: NOTCH2, NOTCH1, FBXW7.

Genes included in “Transcription factor/regulation”: CREBBP, RUNX1, CEBPA, PHF6, WT1, PRDM1, GATA2, GATA3, SETBP1, BCORL1, TERT, KMT2A; Among them, the number of positive cases of RUNX1, CEBPA, PRDM1, GATA2, GATA3, SETBP1, BCORL1, TERT, and KMT2A were all less than 3, which were not suitable for analysis.

Supplementary Table 24. "NOTCH signaling pathway", interaction with specific genes in " Epigenetic modulators " for survival.

| The main signaling pathways | N (%) | 2-year OS (SE) | *P^*^* value | 2-year EFS (SE) | *P^#^* value | 2-year DFS (SE) | *P^&^* value |
| --- | --- | --- | --- | --- | --- | --- | --- |
| **Total** | 55 (100.0) | 83.4 (5.4) |  | 66.9 (7.7) |  | 64.0 (7.9) |  |
| **NOTCH signaling pathway and EZH2** |  |  | 0.547 |  | 0.970 |  | 0.954 |
| No | 51 (92.7) | 84.6 (5.4) |  | 67.2 (7.9) |  | 64.0 (8.3) |  |
| Yes | 4 (7.3) | 66.7 (27.2) |  | 66.7 (27.2) |  | 66.7 (27.2) |  |
| **NOTCH signaling pathway and ASXL2** |  |  | 0.536 |  | 0.445 |  | 0.347 |
| No | 52 (94.5) | 84.2 (5.5) |  | 66.3 (8.2) |  | 63.0 (8.6) |  |
| Yes | 3 (5.5) | 66.7 (27.2) |  | 66.7 (27.2) |  | 66.7 (27.2) |  |
| **NOTCH signaling pathway and KMT2D** |  |  | 0.758 |  | 0.896 |  | 0.680 |
| No | 47 (87.3) | 82.5 (6.1) |  | 63.4 (8.5) |  | 62.5 (8.6) |  |
| Yes | 8 (12.7) | 87.5 (11.7) |  | 87.5 (11.7) |  | 75.0 (15.3) |  |
| **NOTCH signaling pathway and WHSC1** |  |  | 0.437 |  | 0.612 |  | 0.239 |
| No | 52 (94.5) | 82.5 (5.7) |  | 64.8 (8.2) |  | 64.0 (8.3) |  |
| Yes | 3 (5.5) | 100.0 (0.0) |  | 100.0 (0.0) |  | 50.0 (35.4) |  |
| **NOTCH signaling pathway and** **CUX1** |  |  | 0.748 |  | 0.575 |  | 0.476 |
| No | 52 (94.5) | 82.4 (5.7) |  | 70.1 (6.9) |  | 67.1 (7.2) |  |
| Yes | 3 (5.5) | 100.0 (0.0) |  | 50.0 (35.4) |  | 50.0 (35.4) |  |
| **NOTCH signaling pathway and** **RELN** |  |  | **0.001** |  | **0.000** |  | **0.000** |
| No | 52 (94.5) | 85.9 (5.4) |  | 75.5 (6.6) |  | 72.4 (7.0) |  |
| Yes | 3 (5.5) | 50.0 (25.0) |  | 0.0 (0.0) |  | 0.0 (0.0) |  |

T-ALL, T-cell acute lymphoblastic leukemia; No: single positive or negative; Yes: double positive; ^*^significant differences about 2-year OS; ^#^ significant differences about 2-year EFS; ^&^ significant differences about 2-year DFS; Kaplan-Meier method was used to analyze the survival of each group and the differences between subgroups were evaluated using the log-rank test. Bold values indicate statistical significance at p<0.05.

Genes included in “NOTCH signaling pathway”: NOTCH2, NOTCH1, FBXW7.

Genes included in “Epigenetic modulators”: TET2, EP300, EZH2, DNMT3A, DNMT3B, ASXL1, KMT2D, WHSC1, CUX1, RELN, SETD2;

Among them, the number of positive cases of TET2, EP300, DNMT3A, DNMT3B, and SETD2 were all less than 3, which were not suitable for analysis.

**Supplementary Table 25. "NOTCH signaling pathway", interaction with specific genes in "** **Epigenetic modulators " for survival (Univariate analysis)**

| Variables | Overall survival (OS) | | | |  | Event-free survival (EFS) | | |  | Relapse-free survival (RFS) | | |
| --- | --- | --- | --- | --- | --- | --- | --- | --- | --- | --- | --- | --- |
|  | OR | 95%CI (OR) | | *p*-value |  | OR | 95%CI (OR) | *p*-value |  | OR | 95%CI (OR) | p-value |
| NOTCH signaling pathway and EZH2 | 1.881 | | 0.231-15.306 | 0.555 |  | 0.962 | 0.126-7.329 | 0.970 |  | 0.942 | 0.124-7.151 | 0.954 |
| NOTCH signaling pathway and ASXL2 | 1.919 | | 0.234-15.709 | 0.543 |  | 1.786 | 0.391-8.161 | 0.454 |  | 2.012 | 0.451-8.979 | 0.359 |
| NOTCH signaling pathway and KMT2D | 0.723 | | 0.090-5.784 | 0.760 |  | 1.986 | 0.310-3.801 | 0.897 |  | 1.297 | 0.372-4.525 | 0.683 |
| NOTCH signaling pathway and WHSC1 | 0.044 | | 0.000-7610.073 | 0.612 |  | 1.476 | 0.321-6.783 | 0.617 |  | 2.372 | 0.533-10.554 | 0.257 |
| NOTCH signaling pathway and CUX1 | 1.424 | | 0.163-12.454 | 0.749 |  | 1.527 | 0.339-6.874 | 0.581 |  | 1.700 | 0.384-7.515 | 0.484 |
| NOTCH signaling pathway and RELN | 8.075 | | 1.964-33.207 | **0.004** |  | 7.872 | 2.487-24.914 | **0.000** |  | 7.187 | 2.300-22.453 | **0.001** |

T-ALL, T-cell acute lymphoblastic leukemia; Cox regression analysis was used to assess the relationship between various gene mutations and survival; Bold values indicate statistical significance at p<0.05.

Genes included in “NOTCH signaling pathway”: NOTCH2, NOTCH1, FBXW7.

Genes included in “Epigenetic modulators”: TET2, EP300, EZH2, DNMT3A, DNMT3B, ASXL1, KMT2D, WHSC1, CUX1, RELN, SETD2;

Among them, the number of positive cases of TET2, EP300, DNMT3A, DNMT3B, and SETD2 were all less than 3, which were not suitable for analysis.

**Supplementary Table 26. "NOTCH signaling pathway", interaction with specific genes in "** **Epigenetic modulators " for survival (Multivariate analysis)**

| Variables | Overall survival (OS) | | | |  | Event-free survival (EFS) | | |  | Relapse-free survival (RFS) | | |
| --- | --- | --- | --- | --- | --- | --- | --- | --- | --- | --- | --- | --- |
|  | OR | 95%CI (OR) | | *p*-value |  | OR | 95%CI (OR) | *p*-value |  | OR | 95%CI (OR) | p-value |
| NOTCH signaling pathway and EZH2 | 0.812 | | 0.099-6.688 | 0.847 |  | 0.357 | 0.046-2.799 | 0.327 |  | 0.376 | 0.047-2.859 | 0.338 |
| MRD at day 19 | 3.395 | | 1.523-7.565 | **0.003** |  | 2.702 | 1.661-4.396 | **0.000** |  | 2.461 | 1.567-3.865 | **0.000** |
| NOTCH signaling pathway and EZH2 | 1.723 | | 0.204-14.590 | 0.617 |  | 0.801 | 0.104-6.144 | 0.831 |  | 0.821 | 0.107-6.286 | 0.850 |
| MRD at day 46 | 2.821 | | 1.747-4.588 | **0.000** |  | 2.265 | 1.597-3.214 | **0.000** |  | 2.297 | 1.613-3.272 | **0.000** |
| NOTCH signaling pathway and ASXL2 | 1.390 | | 0.171-11.278 | 0.758 |  | 1.104 | 0.240-5.076 | 0.899 |  | 1.189 | 0.260-5.444 | 0.824 |
| MRD at day 19 | 3.359 | | 1.503-7.507 | **0.003** |  | 2.562 | 1.580-4.154 | **0.000** |  | 2.325 | 1.488-3.634 | **0.003** |
| NOTCH signaling pathway and ASXL2 | 3.170 | | 0.351-28.665 | 0.304 |  | 2.392 | 0.496-11.544 | 0.277 |  | 2. 836 | 0.606-13.268 | 0.186 |
| MRD at day 46 | 2.919 | | 1.780-4.788 | **0.000** |  | 2.316 | 1.620-3.311 | **0.000** |  | 2.371 | 1.648-3.411 | **0.000** |
| NOTCH signaling pathway and KMT2D | 2.569 | | 0.268-24.600 | 0.413 |  | 3.315 | 0.799-13.764 | 0.099 |  | 4.962 | 1.132-21.755 | **0.034** |
| MRD at day 19 | 3.479 | | 1.618-7.482 | **0.001** |  | 2.876 | 1.732-4.775 | **0.000** |  | 2.803 | 1.692-4.643 | **0.000** |
| NOTCH signaling pathway and KMT2D | 0.587 | | 0.071-4.834 | 0.620 |  | 1.035 | 0.292-3.665 | 0.957 |  | 1.217 | 0.343-4.314 | 0.761 |
| MRD at day 46 | 2.808 | | 1.759-4.482 | **0.000** |  | 2.266 | 1.595-3.220 | **0.000** |  | 2.299 | 1.611-3.281 | **0.000** |
| NOTCH signaling pathway and WHSC1 | 0.000 | | 0.000- | 0.991 |  | 2.539 | 0.518-12.450 | 0.251 |  | 4.354 | 0.896-20.195 | 0.068 |
| MRD at day 19 | 3.295 | | 1.476-7.357 | **0.004** |  | 2.663 | 1.629-4.355 | **0.000** |  | 2.456 | 1.560-3.865 | **0.000** |
| NOTCH signaling pathway and WHSC1 | 0.000 | | 0.000- | 0.990 |  | 2.288 | 0.456-11.473 | 0.314 |  | 4.267 | 0.885-20.571 | 0.071 |
| MRD at day 46 | 2.749 | | 1.712-4.413 | **0.000** |  | 2.326 | 1.627-3.323 | **0.000** |  | 2.425 | 1.683-3.496 | **0.000** |
| NOTCH signaling pathway and CUX1 | 0.604 | | 0.045-8.089 | 0.703 |  | 1.170 | 0.254-5.403 | 0.840 |  | 1.254 | 0.274-5.740 | 0.771 |
| MRD at day 19 | 3.486 | | 1.524-7.971 | **0.003** |  | 2.560 | 1.581-4.144 | **0.000** |  | 2.325 | 1.491-3.624 | **0.000** |
| NOTCH signaling pathway and CUX1 | 3.264 | | 0.298-35.799 | 0.333 |  | 2.411 | 0.493-11.785 | 0.277 |  | 2.863 | 0.602-13.612 | 0.186 |
| MRD at day 46 | 2.930 | | 1.792-4.790 | **0.000** |  | 2.333 | 1.631-3.337 | **0.000** |  | 2.393 | 1.662-3.445 | **0.000** |
| NOTCH signaling pathway and RELN | 1.849 | | 0.381-8.973 | 0.446 |  | 2.359 | 0.693-8.028 | 0.170 |  | 2.383 | 0.704-8.063 | 0.163 |
| MRD at day 19 | 3.048 | | 1.324-7.017 | **0.009** |  | 2.354 | 1.427-3.885 | **0.001** |  | 2.144 | 1.348-3.408 | **0.001** |
| NOTCH signaling pathway and RELN | 5.287 | | 1.079-25.915 | **0.040** |  | 6.690 | 1.895-23.622 | **0.003** |  | 6.051 | 1.746-20.970 | **0.005** |
| MRD at day 46 | 2.735 | | 1.635-4.576 | **0.000** |  | 2.246 | 1.538-3.281 | **0.000** |  | 2.274 | 1.554-3.328 | **0.000** |

T-ALL, T-cell acute lymphoblastic leukemia; MRD, minimal residual disease; Cox regression analysis was used to assess the relationship between various gene mutations and survival; Bold values indicate statistical significance at p<0.05.

Genes included in “NOTCH signaling pathway”: NOTCH2, NOTCH1, FBXW7.

Genes included in “Epigenetic modulators”: TET2, EP300, EZH2, DNMT3A, DNMT3B, ASXL1, KMT2D, WHSC1, CUX1, RELN, SETD2;

Among them, the number of positive cases of TET2, EP300, DNMT3A, DNMT3B, and SETD2 were all less than 3, which were not suitable for analysis.

Supplementary Table 27. "NOTCH signaling pathway", interaction with specific genes in " Ras/Protein phosphatase/MARK/PI3K signaling pathway " for survival.

| The main signaling pathways | N (%) | 2-year OS (SE) | *P^*^* value | 2-year EFS (SE) | *P^#^* value | 2-year DFS (SE) | *P^&^* value |
| --- | --- | --- | --- | --- | --- | --- | --- |
| **Total** | 55 (100.0) | 83.4 (5.4) |  | 66.9 (7.7) |  | 64.0 (7.9) |  |
| **NOTCH signaling pathway and NRAS** |  |  | 0.507 |  | 0.689 |  | 0.715 |
| No | 52 (94.5) | 82.5 (5.7) |  | 67.5 (7.8) |  | 64.4 (8.1) |  |
| Yes | 3 (5.5) | 100.0 (0.0) |  | 50.0 (35.4) |  | 50.0 (35.4) |  |
| **NOTCH signaling pathway and DNM2** |  |  | 0.399 |  | 0.866 |  | 0.834 |
| No | 50 (90.9) | 82.0 (5.8) |  | 67.2 (7.8) |  | 64.1 (8.1) |  |
| Yes | 5 (9.1) | 100.0 (0.0) |  | 66.7 (27.2) |  | 66.7 (27.2) |  |
| **NOTCH signaling pathway and ARID1A** |  |  | 0.507 |  | 0.361 |  | 0.357 |
| No | 52 (94.5) | 82.5 (5.7) |  | 65.7 (7.8) |  | 62.6 (8.1) |  |
| Yes | 3 (5.5) | 100.0 (0.0) |  | 100.0 (0.0) |  | 100.0 (0.0) |  |
| **NOTCH signaling pathway and USP7** |  |  | 0.498 |  | 0.813 |  | 0.820 |
| No | 52 (94.5) | 82.5 (5.7) |  | 67.8 (7.7) |  | 64.8 (8.0) |  |
| Yes | 3 (5.5) | 100.0 (0.0) |  | 50.0 (35.4) |  | 50.0 (35.4) |  |

T-ALL, T-cell acute lymphoblastic leukemia; No: single positive or negative; Yes: double positive; ^*^significant differences about 2-year OS; ^#^ significant differences about 2-year EFS; ^&^ significant differences about 2-year DFS; Kaplan-Meier method was used to analyze the survival of each group and the differences between subgroups were evaluated using the log-rank test. Bold values indicate statistical significance at p<0.05.

Genes included in “NOTCH signaling pathway”: NOTCH2, NOTCH1, FBXW7.

Genes included in “Ras/Protein phosphatase/MARK/PI3K signaling Pathway”: NRAS, KRAS, NF1, CBL, TP53, DNM2, PTEN, AKT, ETV6, PLCG1, CCND3, ARID1A, BRAF, USP7, DDX3X, BCOR, TRAF3, ANKRD26, ATM, KIT, CXCR4, TEL2;

Among them, the number of positive cases of KRAS, NF1, CBL, TP53, PTEN, AKT, ETV6, PLCG1, CCND3, BRAF, DDX3X, BCOR, TRAF3, ANKRD26, ATM, KIT, CXCR4 and TEL2 were all less than 3, which were not suitable for analysis.

**Supplementary Table 28. "NOTCH signaling pathway", interaction with specific genes in "** **Ras/Protein phosphatase/MARK/PI3K signaling Pathway " for survival (Univariate analysis)**

| Variables | Overall survival (OS) | | | |  | Event-free survival (EFS) | | |  | Relapse-free survival (RFS) | | |
| --- | --- | --- | --- | --- | --- | --- | --- | --- | --- | --- | --- | --- |
|  | OR | 95%CI (OR) | | *p*-value |  | OR | 95%CI (OR) | *p*-value |  | OR | 95%CI (OR) | p-value |
| NOTCH signaling pathway and NRAS | 0.046 | | 0.000-48964.790 | 0.664 |  | 1.504 | 0.196-11.554 | 0.695 |  | 1.453 | 0.19011.100 | 0.719 |
| NOTCH signaling pathway and DNM2 | 0.044 | | 0.000-3191.343 | 0.584 |  | 0.841 | 0.110-6.446 | 0.868 |  | 0.807 | 0.106-6.159 | 0.836 |
| NOTCH signaling pathway and ARID1A | 0.046 | | 0.000-48964.790 | 0.664 |  | 0.046 | 0.000-1241.179 | 0.554 |  | 0.046 | 0.000-1066.390 | 0.548 |
| NOTCH signaling pathway and USP7 | 0.046 | | 0.000-37440.895 | 0.657 |  | 1.274 | 0.166-9.804 | 0.816 |  | 1.264 | 0.164-9.725 | 0.822 |

T-ALL, T-cell acute lymphoblastic leukemia; Cox regression analysis was used to assess the relationship between various gene mutations and survival; Bold values indicate statistical significance at p<0.05.

Genes included in “NOTCH signaling pathway”: NOTCH2, NOTCH1, FBXW7.

Genes included in “Ras/Protein phosphatase/MARK/PI3K signaling Pathway”: NRAS, KRAS, NF1, CBL, TP53, DNM2, PTEN, AKT, ETV6, PLCG1, CCND3, ARID1A, BRAF, USP7, DDX3X, BCOR, TRAF3, ANKRD26, ATM, KIT, CXCR4, TEL2;

Among them, the number of positive cases of KRAS, NF1, CBL, TP53, PTEN, AKT, ETV6, PLCG1, CCND3, BRAF, DDX3X, BCOR, TRAF3, ANKRD26, ATM, KIT, CXCR4 and TEL2 were all less than 3, which were not suitable for analysis.

**Supplementary Table 29. "NOTCH signaling pathway", interaction with specific genes in " Ras/Protein phosphatase/MARK/PI3K signaling Pathway " for survival (Multivariate analysis)**

| Variables | Overall survival (OS) | | | |  | Event-free survival (EFS) | | |  | Relapse-free survival (RFS) | | |
| --- | --- | --- | --- | --- | --- | --- | --- | --- | --- | --- | --- | --- |
|  | OR | 95%CI (OR) | | *p*-value |  | OR | 95%CI (OR) | *p*-value |  | OR | 95%CI (OR) | p-value |
| NOTCH signaling pathway and NRAS | 0.000 | | 0.000- | 0.992 |  | 1.249 | 0.162-9.628 | 0.831 |  | 2.840 | 0.349-23.119 | 0.329 |
| MRD at day 19 | 3.274 | | 1.495-7.169 | **0.003** |  | 2.578 | 1.590-4.180 | **0.000** |  | 2.367 | 1.645-3.407 | **0.000** |
| NOTCH signaling pathway and NRAS | 0.000 | | 0.000- | 0.989 |  | 3.078 | 0.371-25.522 | 0.298 |  | 1.188 | 0.155-9.095 | 0.868 |
| MRD at day 46 | 2.755 | | 1.716-4.426 | **0.000** |  | 2.342 | 1.630-3.365 | **0.000** |  | 2.345 | 1.504-3.656 | **0.000** |
| NOTCH signaling pathway and DNM2 | 0.000 | | 0.000- | 0.987 |  | 0.558 | 0.072-4.313 | 0.576 |  | 0.551 | 0.071-4.256 | 0.576 |
| MRD at day 19 | 3.411 | | 1.549-7.510 | **0.002** |  | 2.562 | 1.608-4.198 | **0.000** |  | 2.370 | 1.521-3.694 | **0.003** |
| NOTCH signaling pathway and DNM2 | 0.000 | | 0.000- | 0.987 |  | 0.292 | 0.036-2.363 | 0.248 |  | 0. 261 | 0.032-2.144 | 0.211 |
| MRD at day 46 | 3.425 | | 1.950-6.017 | **0.000** |  | 2.430 | 1.697-3.480 | **0.000** |  | 2.511 | 1.734-3.636 | **0.000** |
| NOTCH signaling pathway and ARID1A | 0.000 | | 0.000- | 0.991 |  | 0.000 | 0.000- | 0.987 |  | 0.000 | 0.000- | 0.987 |
| MRD at day 19 | 3.322 | | 1.491-7.402 | **0.003** |  | 2.542 | 1.573-4.108 | **0.000** |  | 2.312 | 1.485-3.600 | **0.000** |
| NOTCH signaling pathway and ARID1A | 0.000 | | 0.000- | 0.989 |  | 0.000 | 0.000- | 0.987 |  | 0.000 | 0.000- | 0.987 |
| MRD at day 46 | 2.755 | | 1.716-4.426 | **0.000** |  | 2.223 | 1.563-3.162 | **0.000** |  | 2.255 | 1.579-3.219 | **0.000** |
| NOTCH signaling pathway and USP7 | 0.000 | | 0.000- | 0.991 |  | 0.845 | 0.109-6.562 | 0.872 |  | 0.835 | 0.108-6.484 | 0.863 |
| MRD at day 19 | 3.403 | | 1.542-7.514 | **0.000** |  | 2.575 | 1.594-4.160 | **0.000** |  | 2.347 | 1.508-3.653 | **0.000** |
| NOTCH signaling pathway and USP7 | 0.000 | | 0.000- | 0.990 |  | 0.403 | 0.048-3.352 | 0.400 |  | 0.365 | 0.043-3.093 | 0.355 |
| MRD at day 46 | 3.481 | | 1.970-6.153 | **0.000** |  | 2.393 | 1.659-3.452 | **0.000** |  | 2.462 | 1.690-3.588 | **0.000** |

T-ALL, T-cell acute lymphoblastic leukemia; MRD, minimal residual disease; Cox regression analysis was used to assess the relationship between various gene mutations and survival; Bold values indicate statistical significance at p<0.05.

Genes included in “NOTCH signaling pathway”: NOTCH2, NOTCH1, FBXW7.

Genes included in “Ras/Protein phosphatase/MARK/PI3K signaling Pathway”: NRAS, KRAS, NF1, CBL, TP53, DNM2, PTEN, AKT, ETV6, PLCG1, CCND3, ARID1A, BRAF, USP7, DDX3X, BCOR, TRAF3, ANKRD26, ATM, KIT, CXCR4, TEL2;

Among them, the number of positive cases of KRAS, NF1, CBL, TP53, PTEN, AKT, ETV6, PLCG1, CCND3, BRAF, DDX3X, BCOR, TRAF3, ANKRD26, ATM, KIT, CXCR4 and TEL2 were all less than 3, which were not suitable for analysis.

Supplementary Table 30. " Transcription factor/regulation ", interaction with specific genes in " Epigenetic modulators " for survival.

| The main signaling pathways | N (%) | 2-year OS (SE) | *P^*^* value | 2-year EFS (SE) | *P^#^* value | 2-year DFS (SE) | *P^&^* value |
| --- | --- | --- | --- | --- | --- | --- | --- |
| **Total** | 55 (100.0) | 83.4 (5.4) |  | 66.9 (7.7) |  | 64.0 (7.9) |  |
| **CREBBP and KMT2D** |  |  | 0.726 |  | 0.283 |  | 0.096 |
| No | 51 (92.7) | 83.8 (5.7) |  | 65.2 (8.5) |  | 64.3 (8.6) |  |
| Yes | 4 (7.3) | 75.0 (21.7) |  | 75.0 (21.7) |  | 50.0 (35.4) |  |
| **KMT2D and WT1** |  |  | 0.298 |  | 0.369 |  | 0.381 |
| No | 50 (90.9) | 81.6 (5.9) |  | 62.9 (8.5) |  | 59.5 (8.5) |  |
| Yes | 5 (9.1) | 100.0 (0.0) |  | 66.7 (27.2) |  | 100.0 (0.0) |  |

T-ALL, T-cell acute lymphoblastic leukemia; No: single positive or negative; Yes: double positive; ^*^significant differences about 2-year OS; ^#^ significant differences about 2-year EFS; ^&^ significant differences about 2-year DFS; Kaplan-Meier method was used to analyze the survival of each group and the differences between subgroups were evaluated using the log-rank test. Bold values indicate statistical significance at p<0.05.

Genes included in “Transcription factor/regulation”: CREBBP, RUNX1, CEBPA, PHF6, WT1, PRDM1, GATA2, GATA3, SETBP1, BCORL1, TERT, KMT2A;

Genes included in “Epigenetic modulators”: TET2, EP300, EZH2, DNMT3A, DNMT3B, ASXL1, KMT2D, WHSC1, CUX1, RELN, SETD2;

The number of positive cases was less than 3 in the other pairings of the two groups (“Transcription factor/regulation” and “Epigenetic modulators”) and was not suitable for analysis.

**Supplementary Table 31. "** **Transcription factor/regulation ", interaction with specific genes in "** **Epigenetic modulators " for survival (Univariate analysis)**

| Variables | Overall survival (OS) | | | |  | Event-free survival (EFS) | | |  | Relapse-free survival (RFS) | | |
| --- | --- | --- | --- | --- | --- | --- | --- | --- | --- | --- | --- | --- |
|  | OR | 95%CI (OR) | | *p*-value |  | OR | 95%CI (OR) | *p*-value |  | OR | 95%CI (OR) | p-value |
| CREBBP and KMT2D | 1.449 | | 0.179-11.705 | 0.728 |  | 1.911 | 0.549-7.225 | 0.295 |  | 2.773 | 0.787-9.770 | 0.112 |
| KMT2D and WT1 | 0.042 | | 0.000-452.816 | 0.503 |  | 0.409 | 0.054-3.122 | 0.389 |  | 0.417 | 0.055-3.182 | 0.399 |

T-ALL, T-cell acute lymphoblastic leukemia; Cox regression analysis was used to assess the relationship between various gene mutations and survival; Bold values indicate statistical significance at p<0.05.

Genes included in “Transcription factor/regulation”: CREBBP, RUNX1, CEBPA, PHF6, WT1, PRDM1, GATA2, GATA3, SETBP1, BCORL1, TERT, KMT2A;

Genes included in “Epigenetic modulators”: TET2, EP300, EZH2, DNMT3A, DNMT3B, ASXL1, KMT2D, WHSC1, CUX1, RELN, SETD2;

The number of positive cases was less than 3 in the other pairings of the two groups (“Transcription factor/regulation” and “Epigenetic modulators”) and was not suitable for analysis.

**Supplementary Table 32. "** **Transcription factor/regulation ", interaction with specific genes in " Epigenetic modulators " for survival (Multivariate analysis)**

| Variables | Overall survival (OS) | | | |  | Event-free survival (EFS) | | |  | Relapse-free survival (RFS) | | |
| --- | --- | --- | --- | --- | --- | --- | --- | --- | --- | --- | --- | --- |
|  | OR | 95%CI (OR) | | *p*-value |  | OR | 95%CI (OR) | *p*-value |  | OR | 95%CI (OR) | p-value |
| CREBBP and KMT2D | 3.366 | | 0.339-33.478 | 0.300 |  | 4.730 | 1.090-20.534 | **0.038** |  | 8.506 | 1.858-38.946 | **0.006** |
| MRD at day 19 | 3.394 | | 1.6025-7.189 | **0.001** |  | 2.848 | 1.724-4.705 | **0.000** |  | 2.826 | 1.688-4.731 | **0.000** |
| CREBBP and KMT2D | 1.291 | | 1.141-11.844 | 0.821 |  | 2.299 | 0.603-8.765 | 0.223 |  | 4.001 | 1.050-15.253 | **0.042** |
| MRD at day 46 | 2.755 | | 1.741-4.492 | **0.000** |  | 2.287 | 1.606-3.257 | **0.000** |  | 2.403 | 1.669-3.460 | **0.000** |
| KMT2D and WT1 | 0.000 | | 0.000- | 0.990 |  | 0.935 | 0.109-8.010 | 0.951 |  | 0.995 | 0.117-8.473 | 0.996 |
| MRD at day 19 | 3.175 | | 1.422-7.089 | **0.005** |  | 2.560 | 1.557-4.211 | **0.000** |  | 2.341 | 1.471-3.725 | **0.003** |
| KMT2D and WT1 | 0.000 | | 0.000- | 0.988 |  | 0.620 | 0.078-4.952 | 0.652 |  | 0. 658 | 0.082-5.256 | 0.693 |
| MRD at day 46 | 3.425 | | 1.681-4.340 | **0.000** |  | 2.233 | 1.565-3.186 | **0.000** |  | 2.266 | 1.581-3.248 | **0.000** |

T-ALL, T-cell acute lymphoblastic leukemia; MRD, minimal residual disease; Cox regression analysis was used to assess the relationship between various gene mutations and survival; Bold values indicate statistical significance at p<0.05.

Genes included in “Transcription factor/regulation”: CREBBP, RUNX1, CEBPA, PHF6, WT1, PRDM1, GATA2, GATA3, SETBP1, BCORL1, TERT, KMT2A;

Genes included in “Epigenetic modulators”: TET2, EP300, EZH2, DNMT3A, DNMT3B, ASXL1, KMT2D, WHSC1, CUX1, RELN, SETD2;

The number of positive cases was less than 3 in the other pairings of the two groups (“Transcription factor/regulation” and “Epigenetic modulators”) and was not suitable for analysis.
